# Small-molecule modulators of B56-PP2A restore 4E-BP function to suppress eIF4E-dependent translation in cancer cells 

**DOI:** 10.1172/JCI176093

**Published:** 2025-01-27

**Authors:** Michelle A. Lum, Kayla A. Jonas, Shreya Parmar, Adrian R. Black, Caitlin M. O’Connor, Stephanie Dobersch, Naomi Yamamoto, Tess M. Robertson, Aidan Schutter, Miranda Giambi, Rita A. Avelar, Analisa DiFeo, Nicholas T. Woods, Sita Kugel, Goutham Narla, Jennifer D. Black

**Affiliations:** 1 Eppley Institute for Research in Cancer and Allied Diseases, University of Nebraska Medical Center, Omaha, Nebraska, USA.; 2 Fred and Pamela Buffett Cancer Center, Omaha, Nebraska, USA.; 3 Division of Genetic Medicine, Department of Internal Medicine, and; 4 Rogel Cancer Center, University of Michigan, Ann Arbor, Michigan, USA.; 5 Human Biology Division, Fred Hutchinson Cancer Center, Seattle, Washington, USA.; 6 Department of Pathology and; 7 Department of Obstetrics and Gynecology, University of Michigan, Ann Arbor, Michigan, USA.

**Keywords:** Cell biology, Oncology, Phosphoprotein phosphatases, Signal transduction, Translation

## Abstract

Dysregulated eIF4E-dependent translation is a central driver of tumorigenesis and therapy resistance. eIF4E-binding proteins (4E-BP1/2/3) are major negative regulators of eIF4E-dependent translation that are inactivated in tumors through inhibitory phosphorylation or downregulation. Previous studies have linked PP2A phosphatase(s) to activation of 4E-BP1. Here, we leveraged biased small-molecule activators of PP2A (SMAPs) to explore the role of B56-PP2A(s) in 4E-BP regulation and the potential of B56-PP2A activation for restoring translational control in tumors. SMAP treatment promoted PP2A-dependent hypophosphorylation of 4E-BP1/2, supporting a role for B56-PP2As (e.g., B56α-PP2A) as 4E-BP phosphatases. Unexpectedly, SMAPs induced transcriptional upregulation of 4E-BP1 through a B56-PP2A→TFE3/TFEB→ATF4 axis. Cap-binding and coimmunoprecipitation assays showed that B56-PP2A(s) activation blocks assembly of the eIF4F translation initiation complex, and cap-dependent translation assays confirmed the translation-inhibitory effects of SMAPs. Thus, B56-PP2A(s) orchestrate a translation-repressive program involving transcriptional induction and activation of 4E-BP1. Notably, SMAPs promoted 4E-BP1–dependent apoptosis in tumor cells and potentiated 4E-BP1 function in the presence of ERK or mTOR inhibitors, agents that rely on inhibition of eIF4E-dependent translation for antitumor activity. These findings, combined with the ability of SMAPs to regulate 4E-BP1 in vivo, highlight the potential of PP2A activators for cancer therapy and overcoming therapy resistance.

## Abstract 2

First-in-class small molecule modulators of PP2A restore the function or 4E-BP translational repressors for inhibition of hyperactive protein synthesis, a key vulnerability in cancer cells

## Introduction

The activity of the translational machinery is under tight homeostatic control, and aberrant activation of translation initiation contributes to a variety of pathologies, including cancer (1, 2). Translational rewiring downstream of oncogenic signaling (e.g., RAS/RAF/MEK/ERK, PI3K/AKT/mTOR, Wnt/β-catenin) is a key adaptive mechanism in cancer cells that allows specific changes in the proteome to support tumor development, metastasis, and treatment resistance (1, 3). Several mechanisms of mRNA translation initiation involve recognition of the methyl-7-guanosine (m^7^G) cap at the 5′ end of RNAs, including canonical eIF4E-dependent translation and non-canonical eIF3d- or eIF4G2/DAP5–eIF3d–mediated translation (4, 5). Importantly, eIF4E activity is a key target of oncogenic signals, which promote addiction to hyperactive eIF4E-dependent translation in tumor cells. The rate-limiting step in eIF4E-dependent translation is the assembly of eIF4F, which is initiated by eIF4E binding to the 5′ m^7^GTP cap (1). eIF4E then engages the scaffold protein, eIF4G, and the RNA helicase, eIF4A, to form eIF4F, which in turn recruits the small ribosomal subunit and unwinds secondary structure within the 5′-UTR. eIF4F assembly is negatively controlled by members of the eIF4E-binding protein (4E-BP) family of translational repressors, gatekeeper proteins that act as a nexus or “funnel factors” to integrate effects of signaling pathways on translation (6, 7). 4E-BPs bind to eIF4E to block its interaction with eIF4G, thereby inhibiting eIF4F assembly. Three 4E-BP isoforms exist in mammalian cells, with 4E-BP1 being the most widely expressed and best characterized.

Hyperactive eIF4E-dependent translation plays a central role in reprogramming the proteome in cancer cells by supporting the translation of a subset of “eIF4E-sensitive” or “weak” mRNAs with low translation efficiency due to long, structured 5′-UTRs (1, 8). eIF4E-sensitive mRNAs typically encode potent oncogenic factors that are required for the transformed phenotype (1, 8), including growth factors (e.g., IGF, VEGF), cell cycle regulators (e.g., cyclins D and E), epithelial-to-mesenchymal transition inducers (e.g., Snail), anti-apoptotic proteins (e.g., BCL-xL, MCL1), ROS regulators (e.g., FTH1, GCLC), and transcription factors (e.g., Myc). Thus, tumor cells depend on aberrant eIF4F activity for selective upregulation of critical pro-tumorigenic genes. Mechanisms that prevent 4E-BPs, especially 4E-BP1, from blocking the assembly of eIF4F are, therefore, critical for tumor development and progression (1, 6, 8).

4E-BP1 activity is regulated by hierarchical phosphorylation at Thr37, Thr46, Ser65, and Thr70 (numbering for human 4E-BP1; ref. 9), and by the relative expression of 4E-BP1 and eIF4E (1, 10, 11). In the hypophosphorylated state, 4E-BP1 is active and binds eIF4E, with antiproliferative and tumor-suppressive effects (12, 13). Multisite phosphorylation prevents 4E-BP1 binding to eIF4E, thereby relieving translation repression (6, 7). In many cancers, 4E-BP1 is disabled by inhibitory phosphorylation downstream of oncogenic kinases such as mTOR (the major 4E-BP1 kinase), ERK, p38, CDK1, and CDK4 (7, 14). Tumors also overwhelm the repressive capacity of 4E-BP1 by altering the 4E-BP1/eIF4E ratio through upregulation of eIF4E and/or downregulation of 4E-BP1 (1, 10, 15). Importantly, loss of 4E-BP1 function is a major mechanism of resistance to clinically relevant targeted agents (16), including inhibitors of mTOR (3, 16–20) and the ERK pathway (16, 21–23), which rely on inhibition of eIF4E-dependent translation for their antitumor effects. Therefore, restoration of functional 4E-BP1 is a promising therapeutic strategy with potential for overcoming drug resistance. A better understanding of 4E-BP1 regulation is critical for exploitation of 4E-BP1 function for cancer management.

Studies by our group and others support a role for protein phosphatase 2A (PP2A) serine/threonine phosphatase(s) as 4E-BP1 activators (24, 25). PP2A is a family of heterotrimeric phosphatases consisting of a scaffolding A subunit (Aα, Aβ), a catalytic C subunit (Cα, Cβ), and one of 16 regulatory B subunits (26, 27). B subunits, which fall into 4 structurally distinct classes, B55 (B), B56 (B′), PR72 (B′′), and striatins (B′′′), are the major determinants of substrate selectivity, and their diversity allows PP2A to dephosphorylate a broad spectrum of proteins. Thus, PP2A comprises a family of more than 60 phosphatases that differ in substrate specificity and regulation (26, 27). PP2A activity is widely considered to be tumor suppressive (26), with B56 (α/β/γ/δ/ε) containing holoenzymes emerging as key negative regulators of tumorigenesis (28), and inactivation of PP2A is essential for malignant transformation (28, 29). Notably, unlike other tumor suppressors that are inactivated by genetic mutation, PP2A is primarily disabled by non-genetic mechanisms, including upregulation of endogenous inhibitors (e.g., SET, CIP2A) and posttranslational modification (e.g., L309 carboxymethylation) (26, 28, 29). PP2A is, therefore, available for reactivation in tumors. While evidence points to PP2A as a regulator of 4E-BP1 activity, the PP2A heterotrimers involved and the mechanisms underlying PP2A-mediated 4E-BP1 control are unknown.

In this study, we leveraged recently developed biased small-molecule activators of PP2A (SMAPs) (30–32) to explore the role of B56-containing PP2As in regulation of 4E-BPs and to evaluate the potential of PP2A reactivation for restoring translational control in tumor cells. While protein kinases have long been a major focus of drug development, protein phosphatases have received little attention as therapeutic targets. SMAPs activate PP2As by binding a pocket formed by the A, C, and select B56 subunits, resulting in reduced heterotrimer dissociation rate (31). Structural analysis indicates that SMAPs are selective for B56α-, B56β-, and B56ε-PP2A, and are unable to bind/stabilize heterotrimers containing B56γ, B56δ, or other B subunits. Thus, SMAPs act as a molecular “glue” to activate tumor-suppressive B56α/β/ε-PP2As for selective substrate dephosphorylation. Importantly, PP2A activation by SMAPs has emerged as a viable therapeutic strategy for multiple cancers (e.g., lung, prostate, endometrial, glioblastoma, breast) (26). SMAPs, such as DT-061, show potent anticancer properties in vitro and in vivo, with no detectable toxicity in preclinical studies (26, 32–35). Using SMAPs, we have identified a subset of B56-containing PP2A phosphatase(s) as activators of 4E-BP1 and 4E-BP2 in multiple cell types. Our studies also led to the unexpected finding that B56-PP2A activation promotes transcriptional upregulation of *4E-BP1* via a TFE3/TFEB→ATF4 axis. We further show that SMAPs promote 4E-BP1–dependent apoptosis in tumor cells. Together, our findings support the ability of B56-PP2A(s) to restore translational control by opposing all mechanisms used by tumor cells to disable 4E-BPs. Our demonstration that SMAPs upregulate and activate 4E-BP1 in cancer cells in vivo, and that they can increase 4E-BP1 function in the presence of ERK and mTOR inhibitors, highlights the potential of PP2A-based therapeutics for overcoming resistance to targeted agents that rely on suppression of eIF4F for antitumor activity.

## Results

### SMAPs induce hypophosphorylation of 4E-BP1 and 4E-BP2.

**Figure 1:**
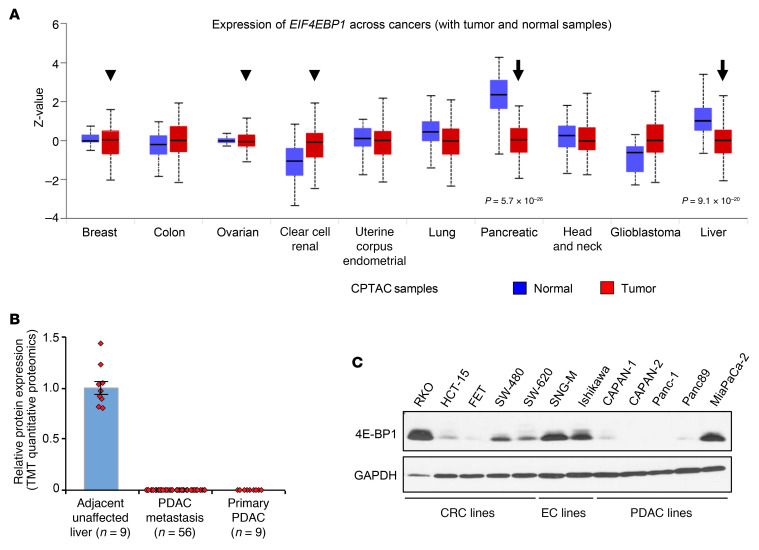
4E-BP1 expression in tumors. (**A**) CPTAC data on the expression of 4E-BP1 (EIF4EBP1) protein in the indicated tissues. Arrows and arrowheads indicate cancers where 4E-BP1 is downregulated or unchanged/upregulated, respectively, relative to normal tissue. (**B**) Liquid chromatography–tandem mass spectrometry (LC-MS/MS) analysis of 4E-BP1 protein expression in primary and metastatic PDACs, with adjacent normal liver as a positive control. (**C**) Immunoblot analysis of 4E-BP1 expression in the indicated cell lines. Data in **C** are representative of at least 3 independent experiments.

Tumor cells disable 4E-BP1 through several mechanisms (1, 7, 11, 15). Analysis of data from The Cancer Genome Atlas (TCGA) and the Clinical Proteomic Tumor Analysis Consortium (CPTAC) shows that many tumors retain or increase the expression of *4E-BP1* mRNA (Supplemental Figure 1; supplemental material available online with this article; https://doi.org/10.1172/JCI176093DS1) and protein (Figure 1A, arrowheads). Importantly, loss of 4E-BP1 function in these tumors is achieved by inhibitory hyperphosphorylation (36–38). Thus, expression of high levels of inactive, hyperphosphorylated 4E-BP1 is a characteristic of many cancers. Tumors also escape translational control by markedly suppressing 4E-BP1 expression (Figure 1A, arrows, and Supplemental Figure 1). 4E-BP1 deficiency is a characteristic of pancreatic ductal adenocarcinomas (PDACs) (15), as confirmed by our proteomic analysis of primary and metastatic tumors (Figure 1B) and by immunoblot analysis of PDAC cell lines (Figure 1C). A survey of colorectal cancer (CRC) cell lines indicated that deficiency of 4E-BP1 was also a characteristic of a subset of colorectal tumors (Figure 1C). In contrast, consistent with TCGA and CPTAC data (Figure 1A and Supplemental Figure 1), the endometrial cancer (EC) cells analyzed (SNG-M, Ishikawa) expressed relatively high levels of 4E-BP1 protein (38).

**Figure 2:**
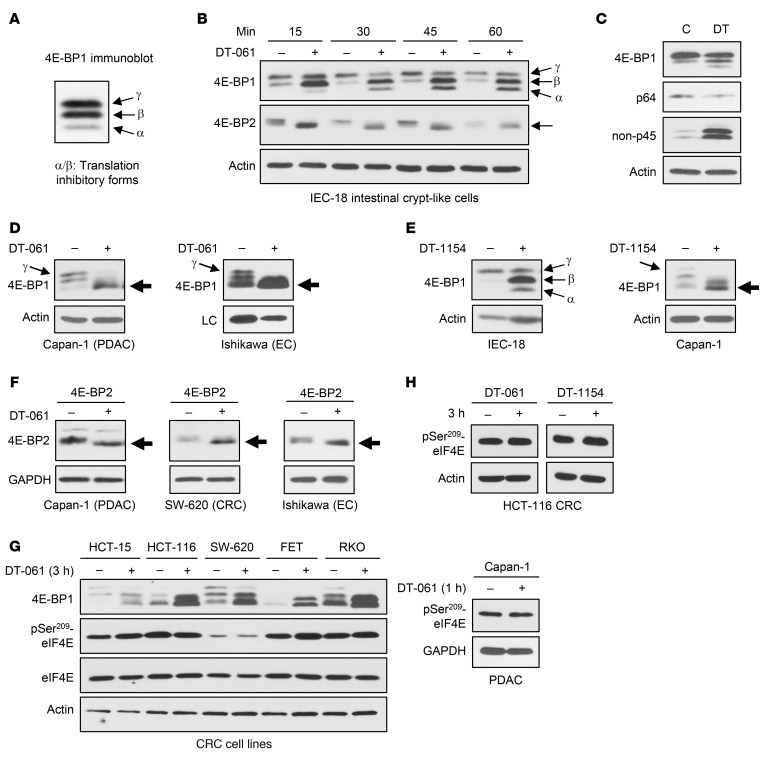
SMAP treatment leads to hypophosphorylation of 4E-BP1 and 4E-BP2. (**A**) Immunoblot of 4E-BP1 from IEC-18 cells showing α, β, and γ phosphoforms. (**B** and **C**) IEC-18 cells were treated with 20 μM DT-061 (+) or vehicle (–) for 15–60 minutes, and expression of the indicated proteins was determined by immunoblotting. Arrows indicate 4E-BP1 phosphoforms and hypophosphorylated 4E-BP2. p64: 4E-BP1 phosphorylated on Ser64; non-p45: 4E-BP1 lacking phosphorylation at Thr45. (**D**) Tumor cells were treated with vehicle or DT-061 before immunoblotting. Large arrows indicate hypophosphorylated 4E-BP1, which accumulated by 1 hour with corresponding loss of the hyperphosphorylated γ form. LC, nonspecific band used as loading control. (**E**) As in **B** and **D**, except that cells were treated with SMAP DT-1154. (**F**) Tumor cells were treated with vehicle or DT-061 for 3 hours before immunoblotting. Arrows indicate hypophosphorylated 4E-BP2. (**G** and **H**) As in **D** and **E** except that immunoblots were also probed for eIF4E and/or phospho-Ser209 eIF4E. All data are representative of 3 or more independent experiments.

Evidence from our group and others supports a role for PP2A as a 4E-BP1 phosphatase (24, 25), although the regulatory B subunits mediating 4E-BP1 activation were not identified. As discussed above, SMAPs only activate PP2A heterotrimers containing B56α, B56β, or B56ε subunits. Thus, we used the lead SMAP, DT-061 (Supplemental Figure 2), to examine the ability of B56α-, B56β-, and/or B56ε-PP2A to activate 4E-BP1 by dephosphorylation. Initial analysis was performed in IEC-18 rat intestinal crypt–like cells, which were used by our group to show that PP2A mediates hypophosphorylation/activation of 4E-BP1 induced by PKCα signaling (24). 4E-BP1 phosphoforms can be distinguished by immunoblotting (Figure 2A): the slower-migrating γ form represents inactive hyperphosphorylated 4E-BP1, and the β/α forms indicate translation-repressive hypophosphorylated species (39). DT-061 treatment led to rapid accumulation of faster-migrating species of 4E-BP1 in these cells (Figure 2B), with robust accumulation of the active β form observed by 15 minutes. By 15–30 minutes, the α form was also readily detectable. DT-061 promoted loss of phosphorylation at Ser64 and Thr45 (Ser65 and Thr46 in human 4E-BP1), as shown using antibodies specific for phosphorylated Ser64 and non-phosphorylated Thr45 (Figure 2C). Note that only fully phosphorylated 4E-BP1 cannot bind eIF4E and loss of Ser64/65 phosphorylation is sufficient for 4E-BP1 activation (6, 9). SMAP-induced accumulation of faster-migrating, hypophosphorylated species of 4E-BP1 was also seen in human cancer cells. Optimization of our immunoblotting technique for detection of human 4E-BP1 phosphoforms (which are more difficult to resolve than rat phosphoforms) revealed a robust mobility shift in low (Capan-1 PDAC) and high (Ishikawa EC) 4E-BP1 expressors by 1 hour of treatment (Figure 2D). Importantly, treatment with DT-1154, another well-characterized SMAP (40) (Supplemental Figure 2), recapitulated the effects of DT-061 on 4E-BP1 (Figure 2E).

DT-061 also promoted rapid accumulation of faster-migrating forms of 4E-BP2 in IEC-18 and tumor cells (Figure 2, B and F, arrows), indicating that both 4E-BP1 and 4E-BP2 can be targeted with SMAPs. As noted by others (41), 4E-BP3 was not detected in the models used in this study (e.g., Supplemental Figure 3); thus, we were unable to determine whether SMAP treatment affects 4E-BP3 phosphorylation. However, in contrast to 4E-BP1/2, 4E-BP3 appears to be predominantly regulated by transcriptional induction rather than phosphorylation (41). Together, the data indicate that SMAP targets, B56α-, B56β-, and/or B56ε-PP2A, modulate phosphorylation of 4E-BP1 and 4E-BP2.

PP2A has been shown to regulate phosphorylation and activity of other components of the translation machinery, including eIF4E and its kinases Mnk1 and Mnk2 (42). Mnk1 and Mnk2 phosphorylate eIF4E at Ser209 to enhance eIF4E cap binding, and PP2A has been shown to negatively regulate Ser209 phosphorylation through dephosphorylation of Mnk1 and eIF4E. However, the SMAPs DT-061 and DT-1154 failed to reduce eIF4E phosphorylation at Ser209 in multiple cell lines (Figure 2, G and H). Thus, these agents do not activate eIF4E- and Mnk-targeting PP2A heterotrimer(s), highlighting the selectivity of SMAPs.

### SMAPs enhance expression of 4E-BP1.

**Figure 3:**
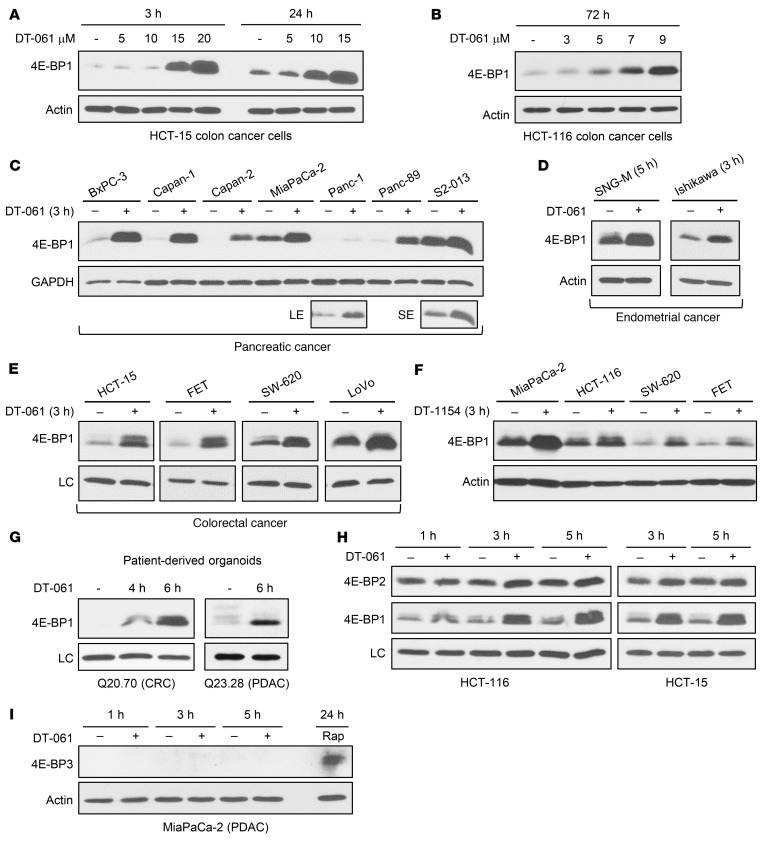
SMAP treatment upregulates 4E-BP1 but not 4E-BP2 or 4E-BP3. (**A**–**E**) CRC, PDAC, or EC cell lines were treated with vehicle or increasing concentrations of DT-061 (**A** and **B**) or 20 μM DT-061 (**C**–**E**) for the indicated times before immunoblot analysis. LE, longer exposure; SE, shorter exposure. (**F**) Cells were treated with 10 μM DT-1154 for 3 hours and subjected to immunoblotting. (**G** and **H**) Cells and PDTOs were treated with 20 μM DT-061 for the indicated times before immunoblot analysis. LC, loading control (actin for Q20.70, GAPDH for Q23.38 and HCT-116, nonspecific band for HCT-15). (**I**) Cells were treated with 20 μM DT-061 or 10 nM rapamycin (Rap) for the indicated times and analyzed by immunoblotting. All data are representative of 3 or more independent experiments.

**Figure 4:**
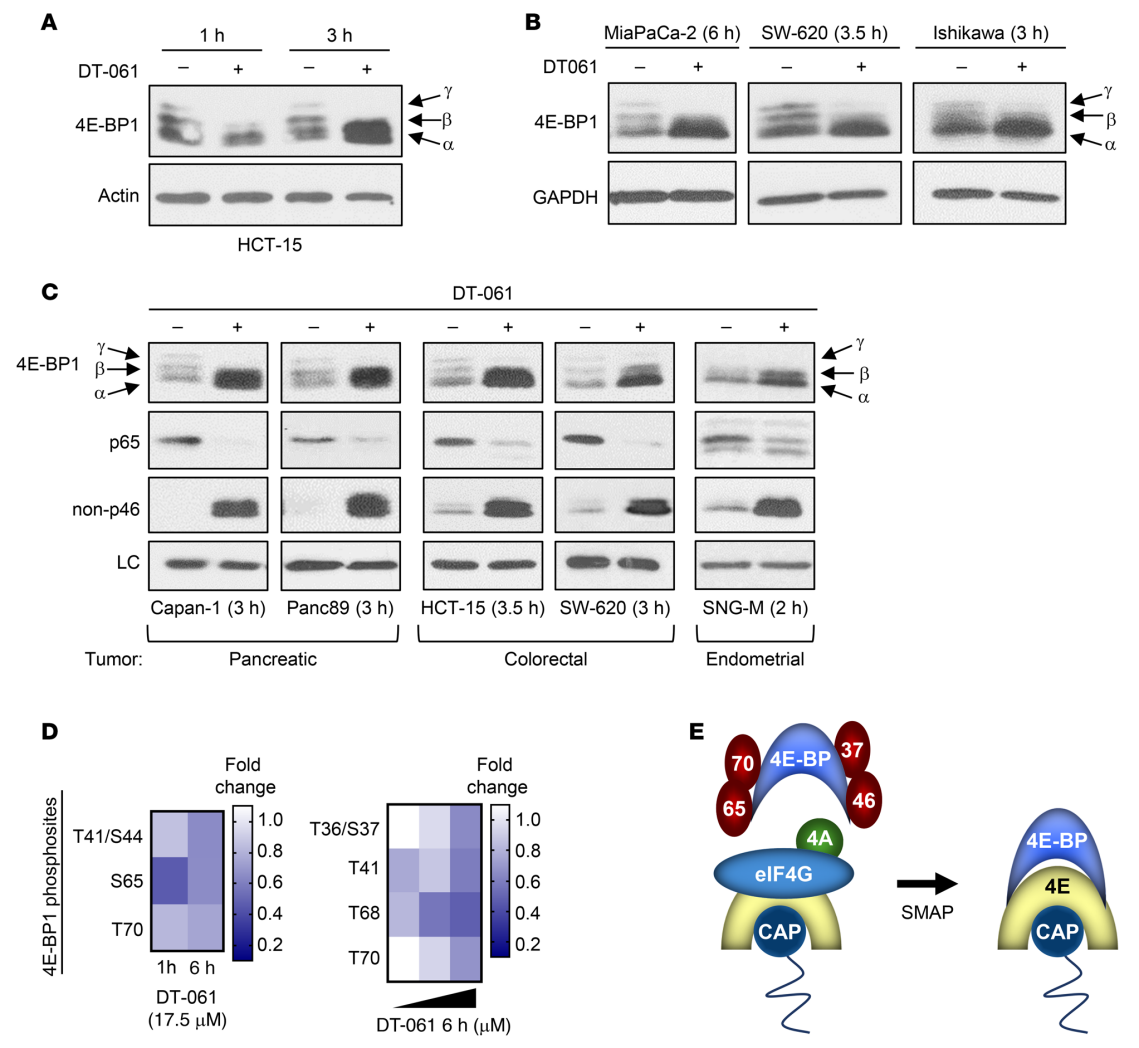
SMAPs promote the accumulation of hypophosphorylated 4E-BP1 in tumor cells. (**A**–**C**) Cells were treated with vehicle or 20 μM DT-061 as indicated before analysis by immunoblotting. LC, GAPDH (Capan-1, Panc89) or actin (HCT-15, SW-620, SNG-M). Data are representative of at least 3 independent experiments. (**D**) Phosphoproteomic analysis of the effects of DT-061 treatment on regulatory phosphorylation of 4E-BP1 in H358 cells. Cells were treated with 17.5 μM DT-061 for 1 hour or 6 hours or with increasing doses (17.5, 22.5, 30 μM DT-061) for 6 hours and analyzed by LC-MS/MS. Heatmaps show the average of 3 biological replicates. (**E**) Model showing effects of SMAP treatment on 4E-BP phosphorylation and eIF4E (4E) binding, with displacement of eIF4G and eIF4A (4A).

Longer DT-061 treatments (e.g., 3 hours) revealed an unexpected ability of SMAPs to promote robust upregulation of 4E-BP1. The effect was concentration dependent, with lower doses giving a slower effect that was sustained for at least 72 hours (Figure 3, A and B). DT-061 treatment induced 4E-BP1 expression in every cell line tested (Figure 3, C–E), and the effect was recapitulated with DT-1154 (Figure 3F). Notably, SMAPs restored 4E-BP1 expression in cells with profound repression of the protein (e.g., many PDAC and CRC cell lines; Figure 3, C, E, and F) and increased 4E-BP1 expression in cells with readily detectable basal levels of the protein (e.g., MiaPaCa-2, S2-013, SNG-M, LoVo; Figure 3, C–F). 4E-BP1 upregulation was also seen in SMAP-treated CRC and PDAC patient-derived tumor organoids (PDTOs) (Figure 3G), models that closely recapitulate the tumors from which they are derived (43). The effect appears to be specific for 4E-BP1 since levels of 4E-BP2 (Figure 3H) and 4E-BP3 (Figure 3I and Supplemental Figure 3) were not affected by prolonged SMAP treatment. (Induction of 4E-BP3 in MiaPaCa-2 cells with rapamycin [ref. 41] served as a positive control.) Since many cancers overwhelm the inhibitory capacity of 4E-BP1 by downregulating 4E-BP1 and/or upregulating eIF4E (1, 10, 11), the finding that SMAPs increase 4E-BP1 levels points to the promise of these agents for restoring the 4E-BP1/eIF4E ratio for translational control in a broad spectrum of cancer types.

Having discovered that SMAPs upregulate 4E-BP1 in tumor cells, we sought to determine whether the newly accumulated protein is also hypophosphorylated. As shown in Figure 4, A and B, 4E-BP1 was exclusively in the hypophosphorylated α/β forms following 3 hours of SMAP treatment (arrows). Loss of phosphorylation was confirmed in multiple cell types using phospho-Ser65–specific and non-phospho–Thr46–specific antibodies (Figure 4C). Phosphoproteomic analysis of DT-061–treated H358 lung cancer cells revealed that SMAPs also induced loss of phosphorylation at Thr70 and Thr36/37 (Figure 4D). Together, the data demonstrate that SMAPs promote upregulation of 4E-BP1 by 3 hours of treatment, accompanied by loss of phosphorylation at all of its major phosphosites (Figure 4E).

### SMAP-induced effects on 4E-BP1 are B56-PP2A dependent.

**Figure 5:**
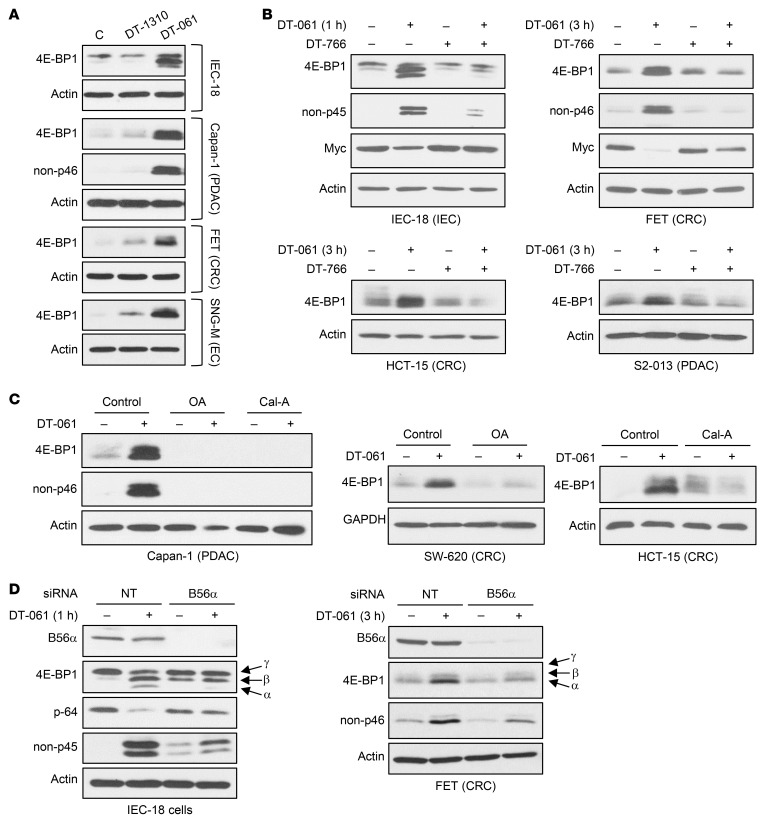
Effects of SMAPs on 4E-BP1 are mediated by PP2A. (**A**) Cells were treated with vehicle, 20 μM DT-1310, or 20 μM DT-061 and analyzed by immunoblotting. Non-p45, non-p46: rat and human 4E-BP1 that lacks phosphorylation at Thr45/46. Cells were treated for 1 hour (IEC-18), 3 hours (Capan-1, FET), or 5 hours (SNG-M). (**B**) As in **A** except that cells were treated with 20 μM DT-061 and 80 μM DT-766 alone or in combination. (**C**) Cell lines were pretreated with 120 nM OA or 6 nM Cal-A before addition of DT-061 as indicated for 3 hours and analysis by immunoblotting. (**D**) Cells were transfected with nontargeting siRNA (NT) or siRNA against B56α (*PPP2R5A*) for 48 hours (IEC-18) or 120 hours (with retransfection at 48 hours, FET) before treatment with vehicle or DT-061 and immunoblot analysis. p-64: 4E-BP1 phosphorylated on Ser64 (numbering for rat 4E-BP1). All data are representative of 3 or more independent experiments.

Several approaches were used to establish PP2A dependence of the effects of SMAPs on 4E-BP1. First, we used DT-1310 and DT-766 (32, 33), compounds that are structurally similar to DT-061 and DT-1154 (Supplemental Figure 2) but are unable to activate PP2A. While DT-1310 does not bind to PP2A, DT-766 binds to the A/C dimer but does not stabilize its association with B subunits. DT-766 thus acts as a competitive inhibitor of PP2A-dependent effects of DT-061. Neither DT-1310 nor DT-766 induced 4E-BP1 upregulation or hypophosphorylation (as indicated by a failure to induce a mobility shift in 4E-BP1 or accumulation of non-phosphorylated Thr46–4E-BP1) (Figure 5, A and B), and a 4-fold excess of DT-766 inhibited the effects of DT-061 (Figure 5B). Downregulation of c-Myc, a known target of SMAPs (31), was used as a positive control for the effects of DT-061 and DT-766 (Figure 5B). PP2A dependence was also confirmed by the ability of pharmacological inhibitors of PP2A, okadaic acid (OA) and calyculin A (Cal-A), to block the effects of SMAPs on 4E-BP1 (Figure 5C).

While the SMAP-binding pocket accommodates B56α, B56β, and B56ε, our previous studies indicate that B56α may be the major regulatory subunit targeted by SMAPs (31, 35); therefore, the role of this subunit was tested. B56α knockdown inhibited DT-061–induced 4E-BP1 hypophosphorylation in IEC-18 and FET cells, as confirmed using phospho-specific antibodies (Figure 5D). B56α knockdown also reduced the 4E-BP1 upregulation observed following 3-hour SMAP treatment in FET cells. These findings establish a role of B56α-PP2A in regulation of 4E-BP1 expression and phosphorylation. However, B56α knockdown did not completely block the effects of SMAP treatment, indicating that B56β-PP2A and/or B56ε-PP2A may also regulate 4E-BP1.

### SMAPs/B56-PP2A activate the translation-repressive functions of 4E-BP1.

**Figure 6:**
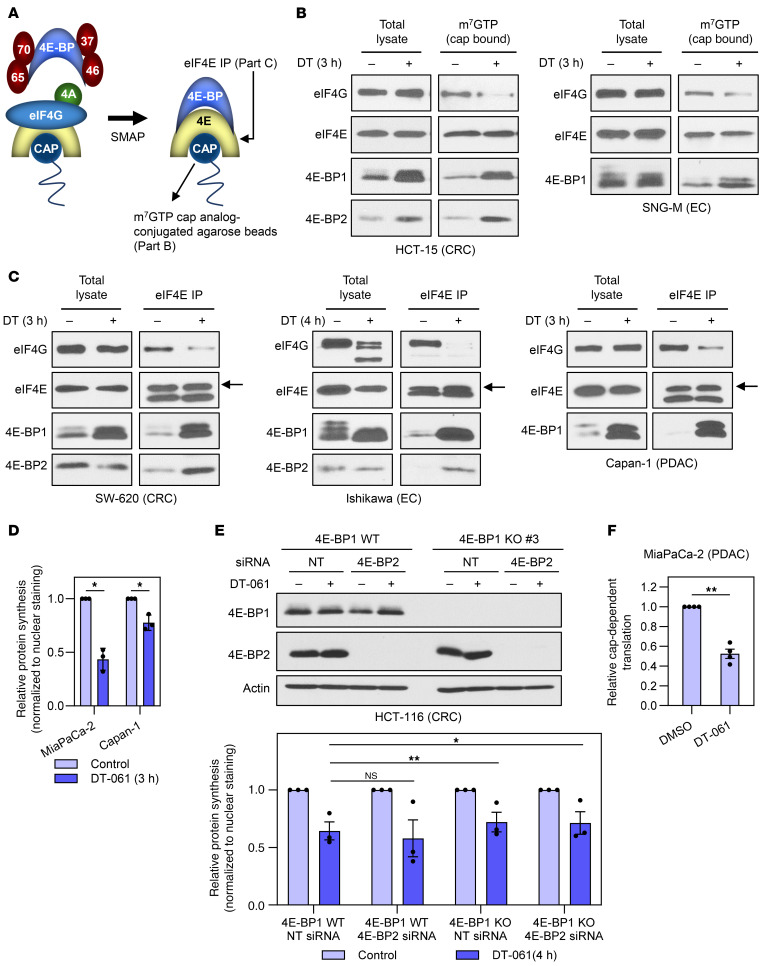
SMAPs/B56-PP2A functionally activate 4E-BPs. (**A**) Schematic showing eIF4E interactions in the presence and absence of active 4E-BPs. (**B**) Cells treated with vehicle or 20 μM DT-061 (DT) for 3 hours were lysed and cap-binding proteins were pulled down using m^7^GTP-agarose. Proteins in cap-associated complexes (Cap Bound) and original lysates (Total Lysate) were analyzed by immunoblotting. (**C**) As in **B** except that eIF4E was immunoprecipitated from lysates (eIF4E IP). Arrows indicate eIF4E migrating above immunoglobulin light chain. Samples in the middle panel were also used in Figure 2D (right panel), with the 4E-BP1 blot shown in both places. (**D**) Cells were treated with vehicle or DT-061 for 3 hours and labeled with l-homopropargylglycine during the last 30 minutes of treatment. l-Homopropargylglycine–containing proteins were then labeled with Alexa Fluor 488, and nuclei were stained with NuclearMask Blue. Alexa Fluor 488 fluorescence was normalized to nuclear staining and expressed relative to that in vehicle-treated cells. (**E**) WT and 4E-BP1–knockout (KO) HCT-116 cells, with or without siRNA-mediated knockdown of 4E-BP2, were treated with vehicle or DT-061 for 4 hours. Cells were then subjected to immunoblot analysis (top) or analyzed for new protein synthesis as in **D** (bottom). NT, nontargeting. (**F**) MiaPaCa-2 cells, stably transfected with the pcDNA3-RLUC-POLIRES-FLUC reporter plasmid, were treated with vehicle or DT-061. After 6 hours, firefly and Renilla luciferase activity was determined, and the ratio of cap-dependent Renilla luciferase and IRES-driven firefly luciferase expression was calculated and expressed as relative to control. Data for each cell line in **B** and **C** are representative of at least 2 independent experiments. Immunoblot data in **E** are representative of 3 independent experiments. Graphical data in **D**, **E**, and **F** are the average (± SEM) of 3, 3, and 4 independent experiments, respectively. *P < 0.05, **P < 0.005 for difference from control (2-sided Student’s *t* test) (**D**), increase over siRNA + DT-061 (Holm-Bonferroni–adjusted 1-sided Student’s *t* test) (**E**), or decrease relative to vehicle control (1-sided Student’s *t* test) (**F**).

Four approaches were used to evaluate the functional consequences of SMAP/B56-PP2A–induced upregulation and dephosphorylation of 4E-BP1: cap affinity assays, eIF4E coimmunoprecipitation (co-IP) assays, pulse labeling with methionine analog l-homopropargylglycine, and cap-dependent translation activity assays (44). While cap affinity assays use m^7^GTP cap analog–conjugated beads to capture eIF4E and eIF4E-associated proteins, co-IP assays use anti-eIF4E antibodies to isolate eIF4E-containing complexes (Figure 6A). Thus, these assays reveal changes in the interaction of 4E-BP1 with eIF4E and in eIF4F assembly. Immunoblot analysis of cap analog–bound proteins revealed that DT-061 promoted a striking increase in levels of cap-associated 4E-BP1 in multiple tumor cell types (Figure 6B and data not shown). Importantly, the increased association of 4E-BP1 with cap analog was accompanied by displacement of eIF4G (Figure 6B), indicating that SMAP-induced 4E-BP1 activation disrupts eIF4F assembly. Consistent with its ability to induce dephosphorylation of 4E-BP2, DT-061 also led to an increase in cap-associated 4E-BP2 (HCT-15 cells; Figure 6B). eIF4E co-IP assays confirmed these findings, with DT-061 inducing a marked increase in 4E-BP1–eIF4E and 4E-BP2–eIF4E interaction, accompanied by a decrease in eIF4E-bound eIF4G in CRC, EC, and PDAC cell lines (Figure 6C and data not shown). Together, these findings confirm that B56-PP2A activation by SMAPs inhibits the formation of the eIF4F translation initiation complex in multiple cancer cell types.

l-Homopropargylglycine labeling revealed that DT-061 inhibited global protein synthesis in cancer cells, with the degree of inhibition varying from 30% to 70% depending on the cell line (Figure 6, D and E). l-Homopropargylglycine labeling of 4E-BP1–knockout/4E-BP2–knockdown HCT-116 cells (confirmed by immunoblotting) was performed to determine whether 4E-PBs contributed to the decrease in total protein synthesis induced by DT-061. This analysis indicated that approximately 25% of the effect required 4E-BP1 in this cell line (Figure 6E). Knockdown of 4E-BP2 did not alter the ability of DT-061 to inhibit total protein synthesis, indicating that 4E-BP1 is the major regulator of eIF4E-dependent translation in these cells. The protein synthesis that persists following SMAP-induced upregulation and activation of 4E-BP1 is likely mediated, in large part, by non-canonical mechanisms of translation initiation (4). As shown in Figure 6F, the ability of DT-061 to suppress eIF4E activity was further supported by suppression of cap-dependent translation, as assessed using a bicistronic dual luciferase reporter that measures the ratio between cap-dependent and cap-independent translation (44). Together, the data show that B56-PP2A activation can counter all mechanisms used by tumor cells to disable 4E-BP1 (i.e., hyperphosphorylation of 4E-BP1, downregulation of 4E-BP1, and increased eIF4E/4E-BP1 ratio) and thereby promote inhibition of eIF4E-dependent translation in multiple cancer cell types.

### SMAPs/B56-PP2A activate transcription of the 4E-BP1 gene (EIF4EBP1).

**Figure 7:**
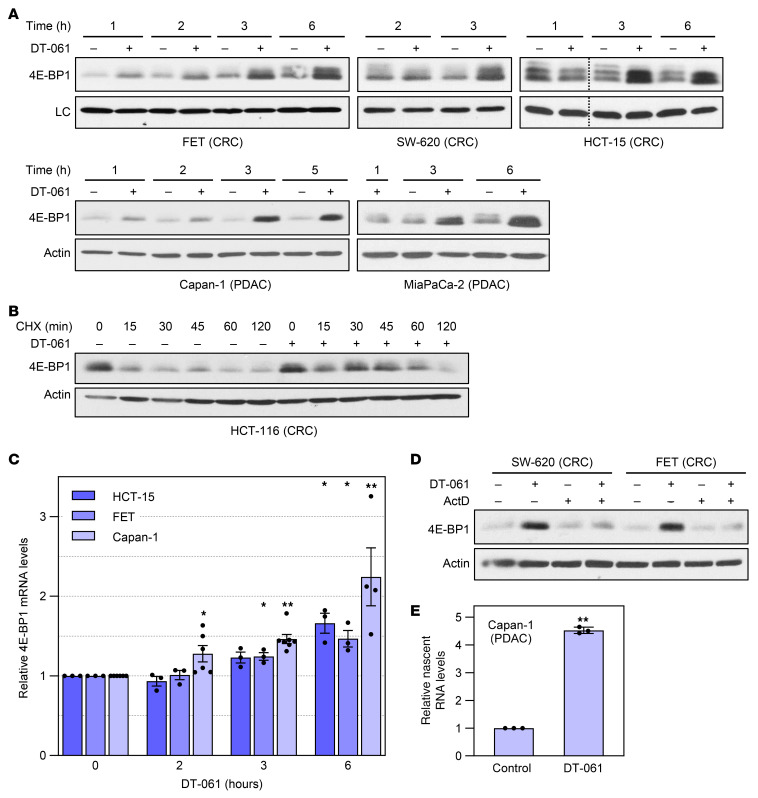
SMAP/B56-PP2A induces 4E-BP1 upregulation at the level of transcription. (**A**) Cells were treated with 20 μM DT-061 as indicated and analyzed by immunoblotting. LC: actin (SW-620, HCT-15, Capan-1, MiaPaCa-2) or GAPDH (FET). Dashed lines indicate rearrangement of lanes from a single blot for clarity. (**B**) HCT-116 cells were treated with DT-061 or vehicle for 1 hour before addition of CHX for the indicated times and analysis by immunoblotting. (**C**) As in **A** except that *4E-BP1* mRNA expression was determined by RT-qPCR, normalized to 18S rRNA, and expressed as relative to control. (**D**) Cells were pretreated with 1 μg/mL ActD for 1 hour as indicated, followed by addition of vehicle or DT-061 for 4 hours before analysis by immunoblotting. (**E**) Cells were treated with vehicle or DT-061 for 6 hours, with 5-ethynyl uridine (EU) added during the last hour. EU-labeled RNA was isolated, and *4E-BP1* mRNA was quantified by RT-qPCR, normalized to 18S rRNA, and expressed relative to control. Data in **A**, **B**, and **D** are representative of at least 3 independent experiments. Data in **C** and **E** are the average (± SEM) of at least 3 independent experiments. *P < 0.05, **P < 0.02 for increase over control (1-sided Student’s *t* test, Holm-Bonferroni adjusted for **C**).

Next, we explored the mechanisms downstream of B56-PP2A that mediate 4E-BP1 upregulation. A modest increase in the levels of 4E-BP1 was sometimes observed following short exposure (1–2 hours) to DT-061 (e.g., Figure 7A). Inhibition of de novo protein synthesis with cycloheximide (CHX) pointed to stabilization of 4E-BP1 following DT-061 treatment (Figure 7B), which may result from increased association of the dephosphorylated protein with eIF4E (45). However, a more robust upregulation of 4E-BP1 was detected beginning at 2–3 hours of treatment (Figure 7A). Reverse transcription and quantitative PCR (RT-qPCR) analysis showed that SMAP treatment upregulated *4E-BP1* mRNA with a similar time course (Figure 7C). Upregulation of 4E-BP1 protein was blocked by the transcription inhibitor actinomycin D (ActD), pointing to a transcriptional mechanism (Figure 7D). The ability of DT-061 to enhance *4E-BP1* mRNA synthesis was further confirmed by 5-ethynyl uridine labeling, which determined that B56-PP2A activation promoted an approximately 4-fold increase in *4E-BP1* mRNA synthesis by 6 hours in Capan-1 cells (Figure 7E). Thus, B56-PP2A activation induces 4E-BP1 expression at the transcriptional level in multiple cancer types.

### Induction of 4E-BP1 transcription by B56-PP2A requires ATF4.

**Figure 8:**
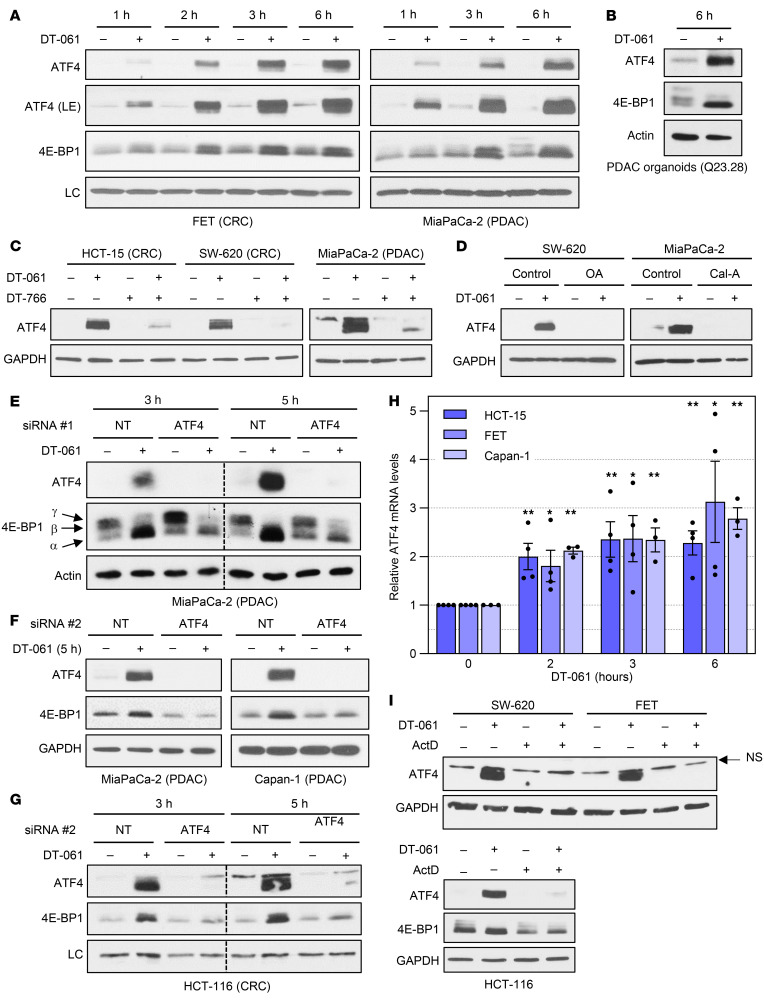
SMAP/B56-PP2A–induced upregulation of 4E-BP1 is dependent on ATF4. (**A** and **B**) Immunoblot analysis of indicated cell lines and PDTOs treated with vehicle or 20 μM DT-061 for 1–6 hours. LE, longer exposure. (**C**) As in **A** except that the cells were treated with vehicle, 20 μM DT-061, and 80 μM DT-766 alone or in combination. (**D**) The indicated cell lines were pretreated with 120 nM OA or 6 nM Cal-A before addition of DT-061 for 3 hours. (**E**–**G**) MiaPaCa-2, Capan-1, or HCT-116 cells were transfected with nontargeting (NT) or ATF4-targeting siRNA (#1 or #2) for 24 hours, before treatment with vehicle or DT-061 as indicated and analysis by immunoblotting. LC, nonspecific band used as loading control. (**H**) The indicated cells were treated with vehicle or DT-061 for various times before RT-qPCR analysis. Levels of *ATF4* mRNA were normalized to 18S rRNA and are expressed as relative to control. (**I**) Cells were pretreated with 1 μg/mL ActD for 1 hour as indicated, followed by addition of vehicle or DT-061 for 4 hours and analysis by immunoblotting. Data in **A**–**D** and **I** are representative of 3 or more independent experiments. Data from each cell line in **E**–**G** are representative of at least 2 independent experiments. Data in **H** are averages (± SEM) of 3 or more independent experiments. *P < 0.05, **P < 0.02 for increase over control (Holm-Bonferroni–adjusted 1-sided Student’s *t* test).

Understanding of the transcriptional regulation of *4E-BP1* is limited (46–49). Previous studies support a role for c-Myc (46), ATF4 (47, 50), HIF1α (48), and SMAD4 (48) as activators of *4E-BP1* transcription. c-Myc can be excluded because it is downregulated by SMAPs (31) (Figure 5B). SMAD4 can also be excluded since SMAPs/PP2A promote 4E-BP1 expression in cells that lack SMAD4 (e.g., BxPC-3, SW-620 [refs. 51, 52]; see Figure 3, C and E). Thus, we examined the role of ATF4. SMAP treatment increased ATF4 levels by 1 hour (Figure 8A), preceding the induction of *4E-BP1* mRNA and protein that generally occurred after 2 hours (Figure 7 and Figure 8A). ATF4 levels continued to increase over 6 hours of treatment (Figure 8A). SMAP-induced upregulation of ATF4 was observed in all cell lines and PDTOs tested (e.g., Figure 8, A–C, F, and G, and data not shown). Consistent with the effects of SMAPs on 4E-BP1, DT-061–induced upregulation of ATF4 was PP2A dependent, as confirmed in competition experiments with a 4-fold excess of DT-766 (Figure 8C) and using the pharmacological inhibitors OA and Cal-A (Figure 8D).

The requirement for ATF4 in SMAP/B56-PP2A upregulation of 4E-BP1 was tested by silencing of ATF4 with 2 siRNAs. As expected, knockdown of ATF4 in MiaPaCa-2 cells did not prevent DT-061–induced 4E-BP1 hypophosphorylation (as indicated by mobility shifts in immunoblots; Figure 8E). In contrast, SMAP-induced upregulation of 4E-BP1 was abrogated when ATF4 was knocked down using either siRNA#1 (Figure 8E) or siRNA#2 (Figure 8F). A role for ATF4 in DT-061–induced 4E-BP1 upregulation was confirmed using siRNA#2 in Capan-1 PDAC cells (Figure 8F) and HCT-116 CRC cells (Figure 8G). Together, the data support a model in which activation of B56-PP2A induces the expression of ATF4, which in turn activates the *4E-BP1* promoter to increase 4E-BP1 expression. This model is consistent with ENCODE ChIP-Seq data showing strong association of ATF4 with a site in intron 1 of *4E-BP1* (Supplemental Figure 4) and with studies in *Drosophila* (50) and pancreatic β cells (47).

### SMAPs/B56-PP2A activate TFE3/TFEB to promote ATF4 and 4E-BP1 expression.

Follow-up experiments explored the mechanisms underlying SMAP/B56-PP2A induction of ATF4. RT-qPCR analysis showed that SMAP treatment upregulated *ATF4* mRNA (Figure 8H), and the ability of ActD to block upregulation of ATF4 pointed to a transcriptional mechanism (Figure 8I). Together, the data indicated that B56-PP2A activation promotes the transcription of *ATF4* in a broad range of cell types.

**Figure 9:**
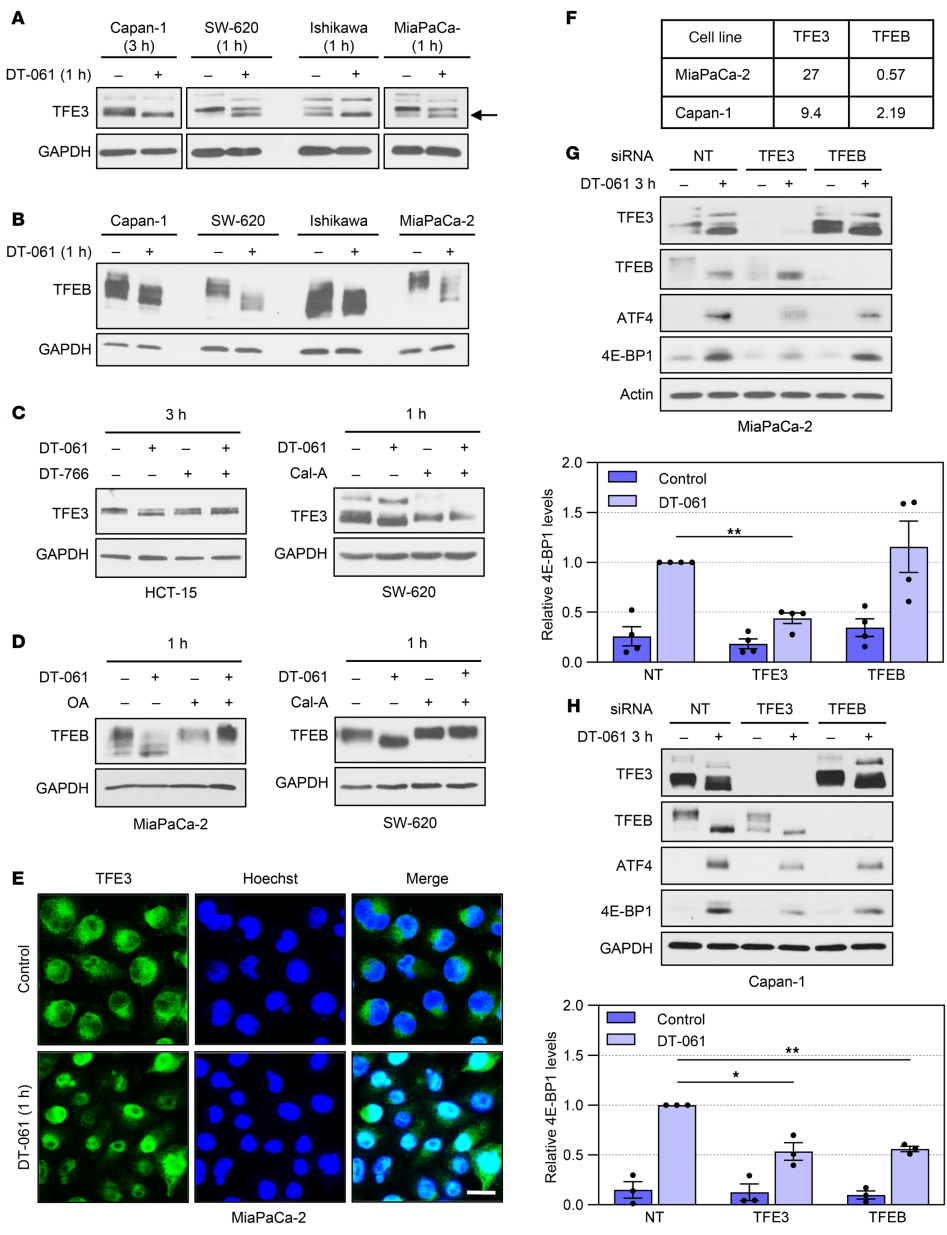
TFE3 and TFEB mediate the effects of SMAP/B56-PP2A on 4E-BP1 expression. (**A**–**D**) Cells were treated with vehicle, 20 μM DT-061, and/or 80 μM DT-766 for the indicated times, and analyzed by immunoblotting. Where specified, cells were pretreated with vehicle, 120 nM OA, or 6 nM Cal-A before the addition of DT-061. (**E**) MiaPaCa-2 cells were treated with vehicle or DT-061 for 1 hour before immunofluorescence staining for TFE3 (green). Nuclei were stained with Hoechst dye (blue). Scale bar: 20 μm. (**F**) Relative expression of *TFE3* and *TFEB* mRNA in the indicated cell lines (data from CCLE). (**G** and **H**) MiaPaCa-2 or Capan-1 cells were transfected with nontargeting (NT) siRNA or siRNA targeting TFE3 or TFEB as indicated for 48 hours before treatment with vehicle or DT-061 for 3 hours. Expression of the indicated proteins was then analyzed by immunoblotting, and relative signal intensity was quantified using ImageJ software. Immunoblot data are representative of at least 3 independent experiments. Graphical data in **G** and **H** are averages (± SEM) of 4 or 3 independent experiments, respectively. *P < 0.05, **P < 0.01 (Holm-Bonferroni–adjusted 1-sided Student’s *t* test).

TFE3 and TFEB are transcription factors that have been implicated in regulation of *ATF4* transcription (53). Nuclear accumulation and transcriptional activity of TFE3 and TFEB require dephosphorylation. SMAP treatment induced a mobility shift of TFE3 (Figure 9A, arrow) and TFEB (Figure 9B) in multiple cell types, an effect that is characteristic of dephosphorylation of these factors (54). The role of PP2A in these changes was confirmed by the ability of DT-766 and the PP2A inhibitors OA and Cal-A to block SMAP-induced shifts in TFE3/TFEB mobility (Figure 9, C and D). Immunofluorescence analysis further confirmed that SMAPs promoted rapid nuclear translocation of TFE3/TFEB (Figure 9E and data not shown). B55α-PP2A has been identified as a TFE3/TFEB phosphatase (55); however, B55α knockdown did not prevent SMAP-induced dephosphorylation of these factors (Supplemental Figure 5), excluding an indirect role of this regulatory subunit. These data provide the first indication that B56-PP2A(s) may be able to dephosphorylate and activate TFE3/TFEB to enhance *ATF4* transcription and promote ATF4-dependent transcriptional activation of *4E-BP1*.

The role of these factors in mediating B56-PP2A–induced 4E-BP1 upregulation was tested in MiaPaCa-2 and Capan-1 cells. Cancer Cell Line Encyclopedia (CCLE) data indicate that MiaPaCa-2 cells express approximately 50-fold more *TFE3* mRNA than *TFEB* mRNA (Figure 9F). In contrast, both factors are well represented in Capan-1 cells (Figure 9F). Knockdown of TFE3 reduced the ability of DT-061 to upregulate ATF4 in both MiaPaCa-2 and Capan-1 cells (Figure 9, G and H). The reduction in ATF4 accumulation was accompanied by decreased 4E-BP1 upregulation, supporting a role for TFE3 in the ATF4-dependent induction of *4E-BP1* promoted by SMAPs. Knockdown of TFEB did not affect SMAP-induced upregulation of ATF4/4E-BP1 in MiaPaCa-2 cells (Figure 9G), consistent with its low expression in these cells. In contrast, TFEB knockdown reduced SMAP-induced ATF4/4E-BP1 upregulation in Capan-1 cells (Figure 9H), indicating that TFEB can also mediate the effect. The same effects were observed using 2 different siRNAs for both TFE3 and TFEB (siRNAs #1 and #2 in Methods). Together, these data point to a pathway in which B56-PP2A activation leads to dephosphorylation and nuclear accumulation of TFE3 and TFEB for transcriptional induction of *ATF4* and subsequent ATF4-mediated upregulation of 4E-BP1.

### Interaction of SMAPs with the ERK and AKT/mTOR signaling pathways.

**Figure 10:**
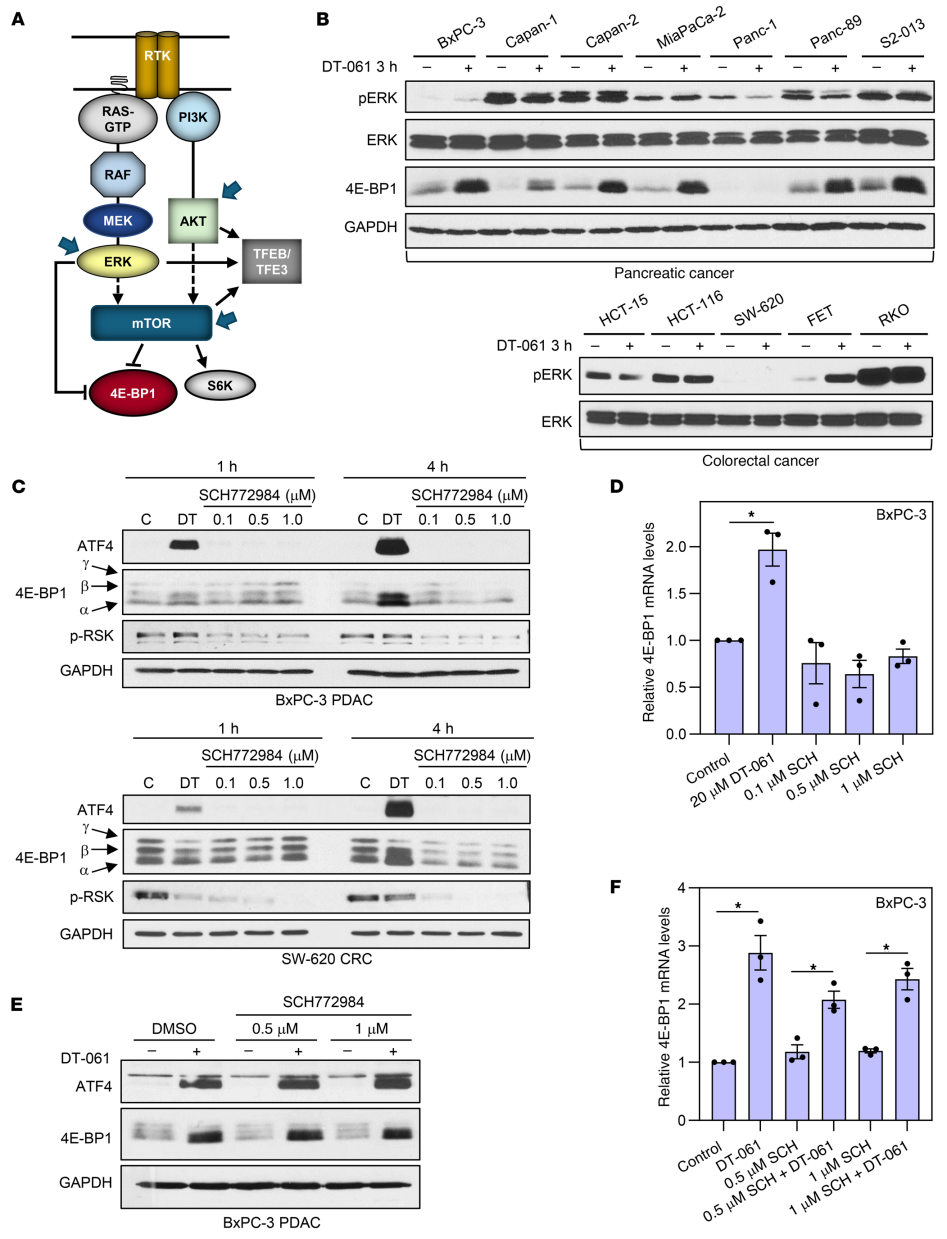
Inhibition of ERK does not account for effects of SMAP/B56-PP2A on 4E-BP1. (**A**) Model showing upstream kinases that regulate 4E-BP1 phosphorylation. mTOR is a major 4E-BP1 kinase, and ERK has also been implicated in phosphorylation of specific sites on the protein. AKT and/or ERK are also upstream regulators of mTOR and TFEB/TFE3. (**B** and **C**) PDAC or CRC cells were treated with vehicle, 20 μM DT-061, or the indicated concentrations of SCH772984 for 1–4 hours before immunoblot analysis. pERK, ERK1/2 phosphorylated on Thr202/Thr204; p-RSK, phospho-RSK. The CRC samples analyzed in **B** are the same as in Figure 2G, which confirms effects on 4E-BP1. (**D**) RT-qPCR analysis of *4E-BP1* mRNA expression (expressed relative to 18S rRNA and normalized to control) in BxPC-3 cells treated as in **C**. (**E**) As in **C** except that cells were preincubated (30 minutes) with SCH772984 before addition of DT-061 for 4 hours. (**F**) As in **D** except that BxPC-3 cells were treated as in **E**. Immunoblot data are representative of 3 or more independent experiments, and RT-qPCR data are averages (± SEM) of 3 independent experiments. *P < 0.05 for increase induced by DT-061 (Holm-Bonferroni–adjusted 1-sided Student’s *t* test).

PP2A has been implicated in negative regulation of ERK and AKT/mTOR kinases. Since ERK, AKT, and/or mTOR have been identified as 4E-BP1 and TFE3/TFEB kinases (6, 7, 56) (Figure 10A), SMAPs could affect 4E-BP1 through inhibition of these upstream regulators. The relationship between effects of SMAPs on 4E-BP1 and these pathways is also important because a major mechanism of resistance to targeted inhibitors of ERK and mTOR signaling is a failure to induce 4E-BP1 activity for suppression of eIF4F function (16). While previous studies have shown ERK inhibition following prolonged SMAP treatment (e.g., 24 hours) (32), our analysis revealed that ERK phosphorylation/activity was unaffected by DT-061 following shorter treatments (1–4 hours) in a majority of cell lines tested (Figure 10B). The rapid effects of SMAPs on 4E-BP1 are, therefore, unlikely to be mediated by alterations in ERK activity. ERK independence was confirmed using the ERK inhibitor SCH772984. Although ERK has been reported to function as a 4E-BP1 kinase in some studies (7), inhibition of ERK with SCH772984 (confirmed by analysis of phospho-RSK levels) did not result in hypophosphorylation of 4E-BP1 in either BxPC-3 or SW-620 cells (Figure 10C), likely reflecting the major role of mTOR as a 4E-BP1 kinase (see below). Similarly, SCH772984 failed to induce ATF4 or upregulate *4E-BP1* mRNA or protein in these cells (Figure 10, C and D). Importantly, SCH772984 treatment did not affect the ability of SMAPs to promote dephosphorylation of 4E-BP1, upregulation of ATF4, or induction of *4E-BP1* mRNA/protein (Figure 10, E and F), confirming that these effects of SMAPs are independent of ERK.

**Figure 11:**
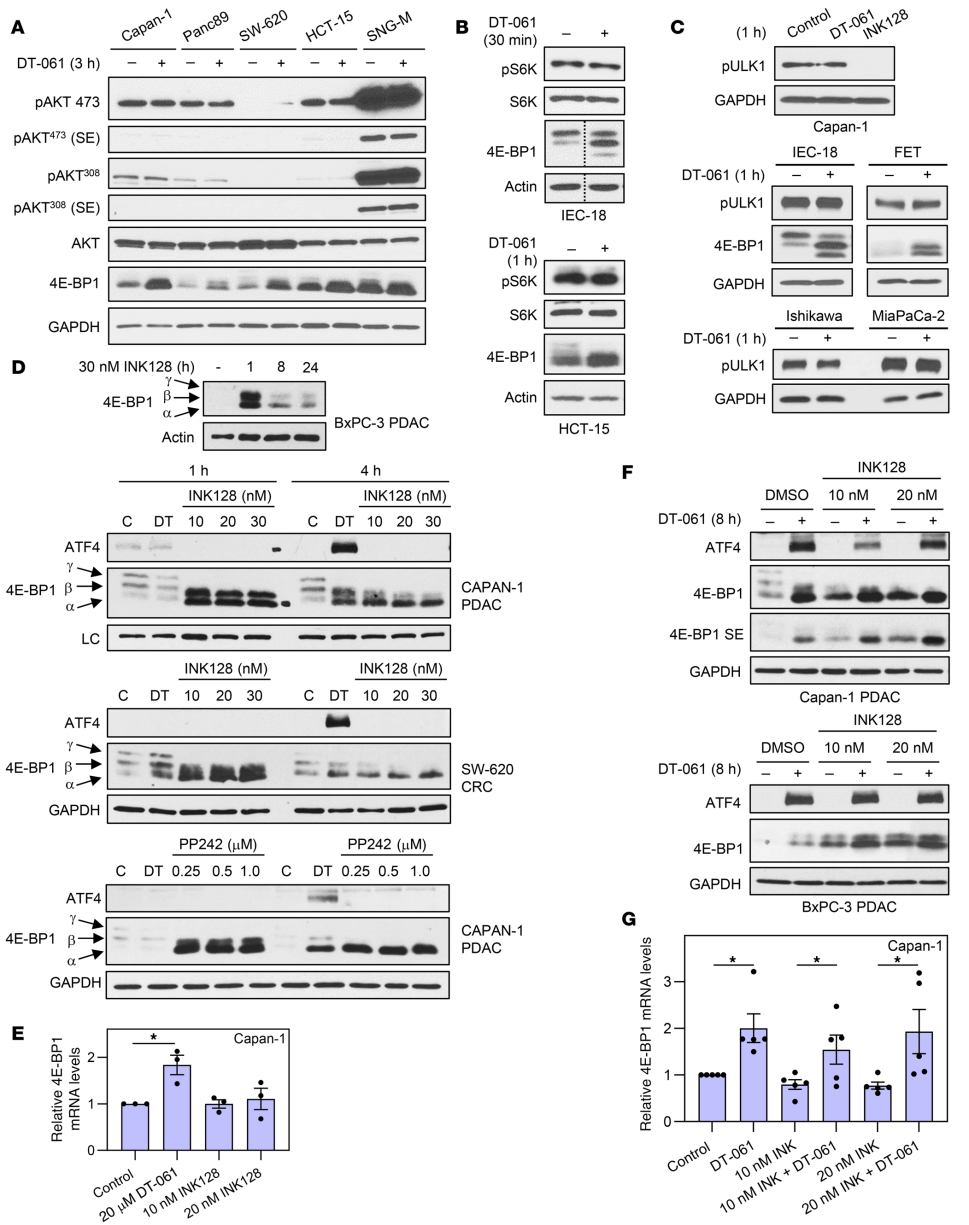
Inhibition of AKT/mTOR signaling does not account for effects of SMAP/B56-PP2A on 4E-BP1. (**A**–**C**) Cells were treated with vehicle, 20 μM DT-061, or INK128 for the indicated times before immunoblot analysis. pAKT, AKT phosphorylated on Ser473 or Thr308 as indicated; pS6K, S6 kinase phosphorylated on Thr389; pULK1, ULK1 phosphorylated on Ser757; SE, shorter exposure. (**D** and **F**) Immunoblot analysis of cells treated with DT-061 or 10–30 nM INK128 for the indicated times (**D**) or pretreated (30 minutes) with the indicated concentrations of INK128 before addition of DT-061 for 4 hours (**F**). (**E** and **G**) RT-qPCR analysis of *4E-BP1* mRNA levels (expressed relative to 18S rRNA and normalized to control) in cells treated as in **D** and **F**. Immunoblot data are representative of 3 or more independent experiments. Graphical data are averages (± SEM) of 3 (**E**) or 5 (**G**) independent experiments. *P < 0.05 for increase induced by DT-061 (Holm-Bonferroni–adjusted 1-sided Student’s *t* test).

Analysis of activating Ser473 and Thr308 phosphorylation of AKT excluded effects of SMAP treatment on AKT activation (Figure 11A), consistent with evidence that B55α-PP2A, but not B56-PP2A, dephosphorylates AKT (57). DT-061 treatment also failed to affect mTOR activity, as reflected in phosphorylation of the mTOR substrates S6 kinase and ULK1: while the mTOR inhibitor INK128 blocked ULK1 phosphorylation, SMAPs failed to affect the phosphorylation of these proteins (Figure 11, B and C). The role of mTOR in the effects of SMAPs was further examined using the mTOR inhibitors INK128 and PP242. As expected, mTOR inhibition led to robust hypophosphorylation of 4E-BP1 in BxPC-3/Capan-1 PDAC cells and SW-620 CRC cells, indicating that mTOR is the major 4E-BP1 kinase in these cells (Figure 11D). Interestingly, mTOR inhibition led to a marked increase in 4E-BP1 levels by 1 hour (Figure 11D). However, in contrast to DT-061, mTOR inhibitors did not induce upregulation of ATF4 or *4E-BP1* mRNA (Figure 11, D and E), pointing to a post-transcriptional mechanism. CHX chase assays showed that mTOR inhibition led to stabilization of 4E-BP1 protein (Supplemental Figure 6), paralleling the early upregulation of 4E-BP1 seen with SMAP-induced dephosphorylation of the protein (Figure 7B). Notably, the upregulation induced by INK128 or PP242 was transient, with levels of 4E-BP1 decreasing over time (e.g., 4 and 8 hours; Figure 11D). This effect cannot be attributed to inactivation of the inhibitor, since hypophosphorylation of 4E-BP1 was maintained throughout the experiment (Figure 11D). The transient effect of mTOR inhibition is consistent with the enhanced stability of eIF4E-bound hypophosphorylated 4E-BP1, together with enhanced ubiquitination of 4E-BP1 observed after 24-hour treatment with PP242 (45). Notably, combined treatment with INK128 and DT-061 resulted in prolonged 4E-BP1 protein upregulation and activation, in association with increased expression of ATF4 and *4E-BP1* mRNA (Figure 11, F and G). Taken together, the data indicate that, as with ERK, effects of SMAPs on 4E-BP1 are independent of effects on mTOR. Changes in 4E-BP1 phosphorylation, therefore, likely reflect direct dephosphorylation by B56α-PP2A, and possibly by B56β-PP2A and/or B56ε-PP2A. The data also point to the therapeutic potential of combining SMAPs with ERK and mTOR inhibitors, which rely on sustained suppression of eIF4E-dependent translation for their antitumor activity.

### SMAPs induce 4E-BP1–dependent apoptosis.

**Figure 12:**
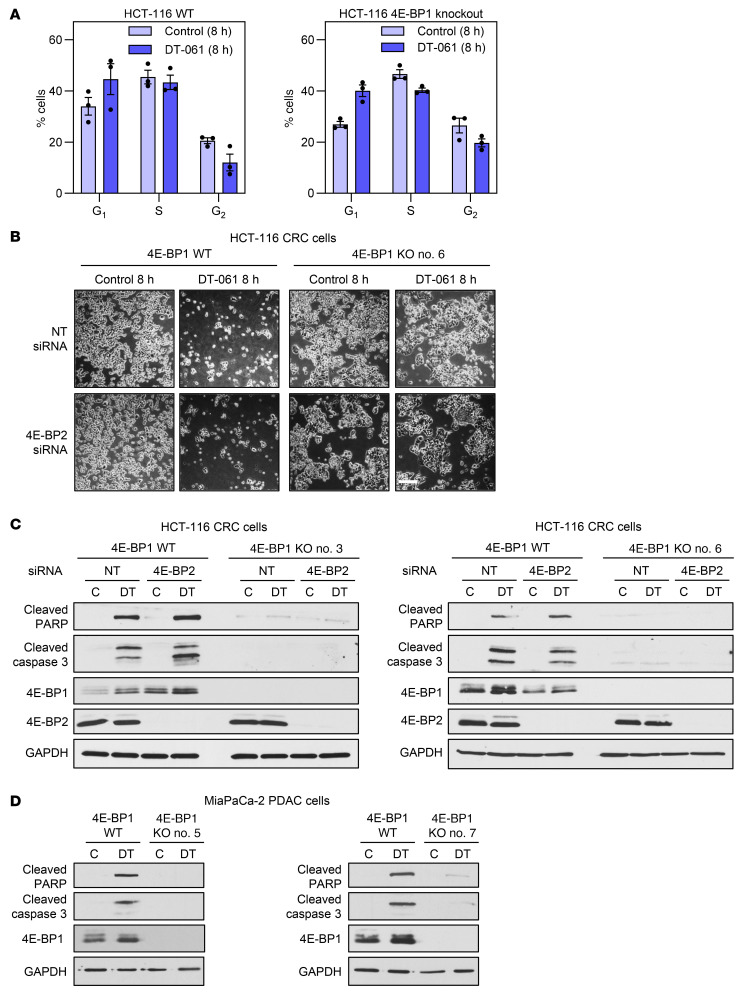
DT-061 induces G_1_ arrest and 4E-BP1–dependent apoptosis. (**A**) WT or 4E-BP1–knockout HCT-116 cells were treated with vehicle or 20 μM DT-061 for 8 hours, stained with propidium iodide, and subjected to flow cytometric analysis of cell cycle distribution. (**B**) WT and 4E-BP1–knockout (KO) HCT-116 cells were transfected with nontargeting (NT) or 4E-BP2–targeting siRNA for 48 hours and treated with vehicle or 20 μM DT-061 for 8 hours. Phase contrast images are shown. Scale bar: 200 μm. (**C** and **D**) WT and 4E-BP1–knockout (KO) HCT-116 cells (with or without 4E-BP2 knockdown) (**C**) or MiaPaCa-2 cells (**D**) were treated with vehicle or DT-061 for 8 hours and analyzed by immunoblotting. All data are representative of at least 3 independent experiments.

To determine the role of 4E-BP1 in the antitumor effects of SMAPs, we analyzed the effects of DT-061 on cell proliferation and cell death in HCT-116 cells with or without knockout of 4E-BP1. Treatment of these cells with DT-061 for 8 hours led to an increase in cells in G_1_ and a decrease in cells in G_2_/M, pointing to G_1_ blockade (Figure 12A). The effects were similar in wild-type and 4E-BP1–knockout cells, indicating that cell cycle blockade is largely mediated by other B56-PP2A substrates. Treatment of wild-type HCT-116 cells for 8 hours also led to a profound change in cell morphology (Figure 12B), reflecting induction of apoptosis as confirmed by cleavage of PARP and caspase-3 (Figure 12C). Notably, while knockdown of 4E-BP2 did not affect the ability of DT-061 to induce apoptosis in these experiments, pro-apoptotic effects of the SMAP treatment were not seen in either 4E-BP1–knockout clone tested. These effects were recapitulated in MiaPaCa-2 PDAC cells (Figure 12D). The ability of SMAPs to induce 4E-BP1–dependent apoptosis points to 4E-BP1 as an important target for the antitumor effects of these B56-PP2A agonists.

### SMAPs promote hypophosphorylation and upregulation of 4E-BP1 in vivo.

**Figure 13:**
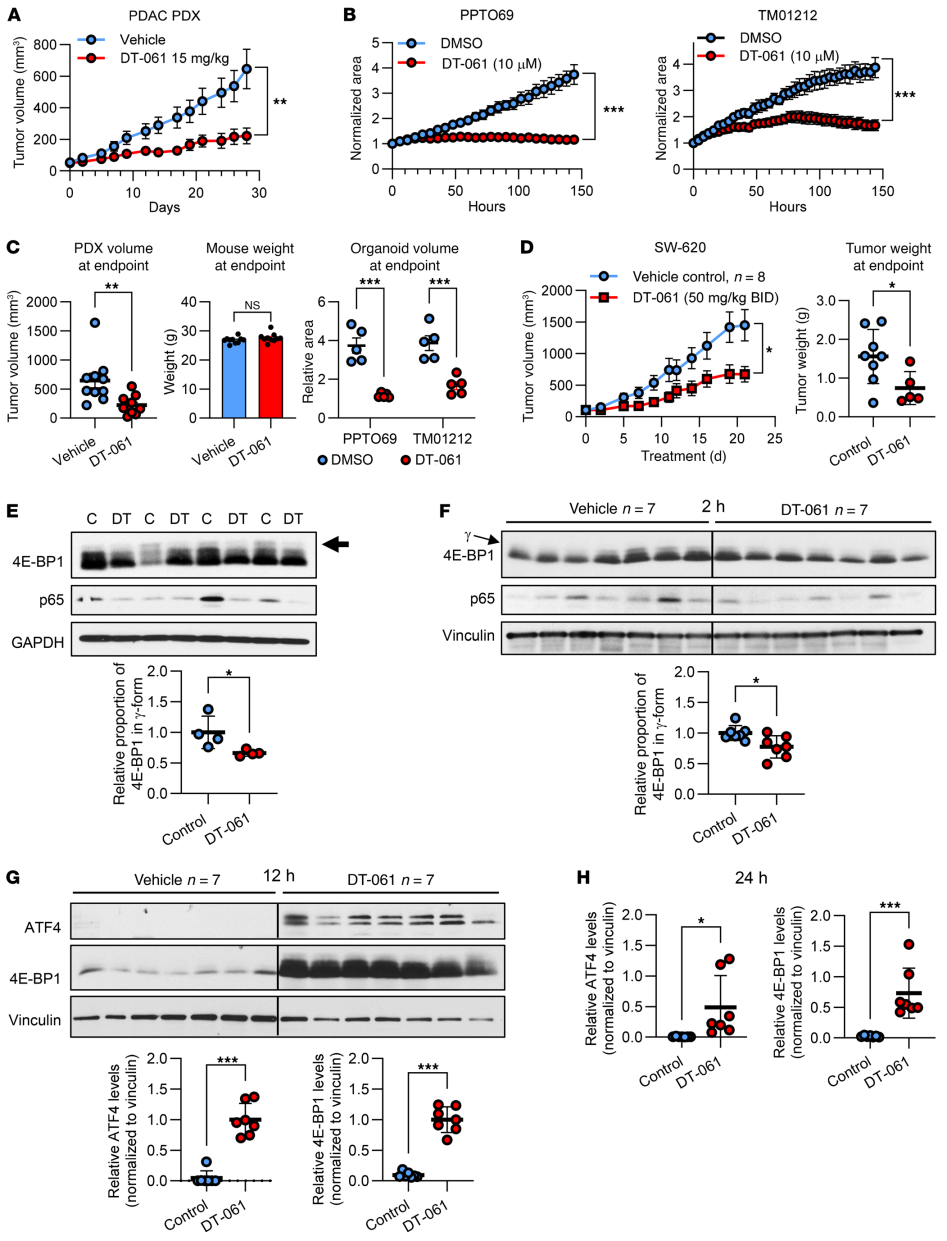
DT-061 induces upregulation and hypophosphorylation of 4E-BP1 in association with tumor suppression in organoids and mouse xenografts. (**A**) Athymic nude mice bearing subcutaneous PDAC PDXs were treated with vehicle or 15 mg/kg DT-061 twice daily (10 per group), and tumor volume was estimated using calipers. (**B**) PDAC PDTOs were treated with vehicle or 10 μM DT-061, and organoid total area (μm^2^/image) was measured using an Incucyte SX5 (Sartorius). Areas were normalized to time zero and are presented as averages (± SEM, *n* = 5). (**C**) Endpoint data for **A** and **B**. PDX volume and mouse weight are for the 28-day time point, and PDTO volumes are for 144 hours of treatment. (**D**) As in **A** except that mice bearing subcutaneous SW-620 xenografts were treated with vehicle or 50 mg/kg DT-061 twice daily and tumors were excised and weighed after 21 days. (**E**) Immunoblot analysis of extracts of SW-620 tumors harvested 3 hours after mice were treated with vehicle (C, control) or 15 mg/kg DT-061 (DT). The graph shows quantification of the signal for the slowest-migrating γ form of 4E-BP1 expressed relative to the corresponding total signal for 4E-BP1. (**F**–**H**) As in **E**, except that mice were treated with vehicle or 50 mg/kg DT-061 for 2 hours, 12 hours, or 24 hours. Note that the difference in 4E-BP1 signal in control cells at 12 hours compared with 2 hours reflects different exposure times necessitated by the intensity of signal in treated lanes at 12 hours. Graphs show quantification of the total signal for ATF4 and 4E-BP1 expressed relative to the corresponding signal for vinculin. *P < 0.05, **P < 0.01, ***P < 0.001 (2-sided Student’s *t* test).

Treatment of mice bearing PDAC patient-derived xenografts (PDXs) or PDTOs with DT-061 confirmed that SMAPs have pronounced in vivo and in vitro antitumor activity, with no detectable in vivo toxicity (Figure 13, A–C). DT-061 (50 mg/kg, twice a day) gave similar results in mice bearing subcutaneous xenografts generated using SW-620 CRC cells (Figure 13D), highly tumorigenic and metastatic cells that are resistant to a broad spectrum of chemotherapeutic and targeted agents (18, 58). To determine whether SMAPs/PP2A can hypophosphorylate 4E-BP1 in SW-620 xenografts, tumors were harvested 2–3 hours after administration of DT-061 at 15 mg/kg or 50 mg/kg DT-061 and subjected to anti–4E-BP1 immunoblot analysis (Figure 13, E and F). SMAP treatment reduced phosphorylation of 4E-BP1 in these tumors, as indicated by a decrease in the proportion of 4E-BP1 in the hyperphosphorylated, γ form (Figure 13E, arrow) and by a reduction in phosphorylation at Ser65. As expected, total levels of 4E-BP1 were not affected at this time. However, treatment with 50 mg/kg DT-061 for 12 hours resulted in marked upregulation of ATF4 and 4E-BP1 expression in SW-620 tumors (Figure 13G). The effect was sustained at 24 hours, albeit at a reduced level (Figure 13H), likely reflecting reduced activity of DT-061 by this time point (32, 33). Thus, consistent with our in vitro data, DT-061 can modulate the phosphorylation and expression of 4E-BP1 in tumor cells in vivo, supporting the clinical potential of PP2A-based therapeutics for restoration of translational control in cancer.

## Discussion

**Figure 14:**
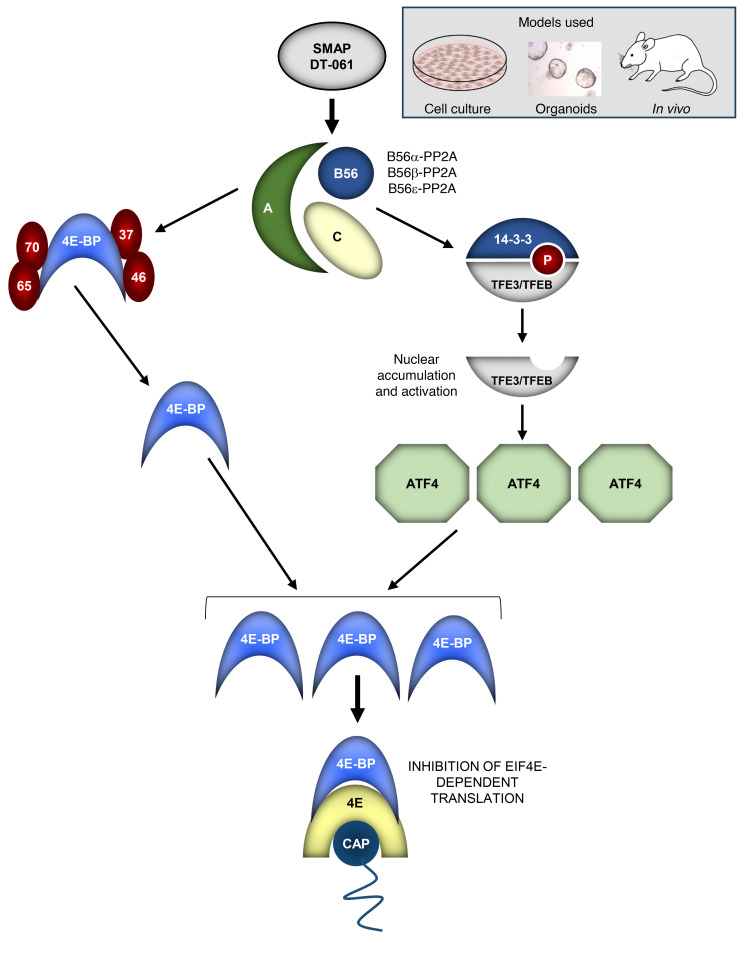
Model for the effects of SMAPs on 4E-BPs. SMAPs such as DT-061 and DT-1154 activate a subset of B56-PP2A heterotrimers (B56α-, B56β-, B56ε-PP2A) to dephosphorylate 4E-BP1 and 4E-BP2 at canonical sites. SMAPs/B56-PP2A also dephosphorylate and activate the transcription factors TFE3/TFEB to induce *ATF4* transcription. ATF4 accumulation activates transcription of the *4E-BP1* gene, leading to upregulation of 4E-BP1 protein that is also dephosphorylated by B56-PP2A. Active 4E-BP1 prevents formation of the eIF4F translation initiation complex and inhibits eIF4E-dependent translation.

By leveraging novel small-molecule activators of PP2A with selectivity for a subset of PP2A heterotrimers (26, 30, 31), this study shows that B56-PP2A(s) orchestrate a translation-repressive program in tumor cells involving transcriptional upregulation of *4E-BP1* and dephosphorylation of 4E-BP1 and 4E-BP2 (Figure 14). Remarkably, SMAPs/B56-PP2A can restore translational control in tumor cells with profound suppression of 4E-BP1 expression (e.g., many PDAC and CRC cell lines) by engaging a TFE3/TFEB→ATF4→4E-BP1 transcriptional axis (Figure 14). The 4E-BP1 protein that accumulates following SMAP treatment is in the active, translation-repressive form as a result of B56-PP2A–mediated hypophosphorylation.

To our knowledge, our study provides the first evidence that B56-PP2A heterotrimer(s) can activate 4E-BP1 and 4E-BP2 by hypophosphorylation. B56-PP2A activation reduces phosphorylation at Thr37, Thr46, Thr70, and Ser65 and thus targets all canonical 4E-BP phosphosites. While previous studies have linked PP2A to regulation of 4E-BP phosphorylation (25), the regulatory subunit(s) that target 4E-BP were not defined. Our knockdown studies support a role for B56α, a major regulatory subunit targeted by SMAPs (31), although the data suggest that B56β and/or B56ε are also involved. The demonstration that SMAP/B56-PP2A–induced 4E-BP hypophosphorylation is independent of effects on upstream kinases such as mTOR, AKT, and ERK indicates that B56-PP2A(s) do not activate 4E-BPs by blocking their phosphorylation; instead, these results point to B56-PP2A(s) as direct 4E-BP phosphatases. This conclusion is consistent with the demonstration that PP2A-C can dephosphorylate GST–4E-BP1 in vitro (59), although physical interaction between B56-PP2A(s) and 4E-BPs remains to be demonstrated.

Based on the function of SMAPs as PP2A activators, a major goal of this study was to test whether these compounds could induce 4E-BP dephosphorylation and thus reestablish homeostatic translational control in cancer cells. The finding that SMAPs also restore and upregulate 4E-BP1 expression via a transcriptional mechanism was unexpected. Our data show that B56-PP2A activation rapidly induces expression of *ATF4* at the mRNA and protein levels, and that ATF4 plays a requisite role in B56-PP2A–mediated upregulation of 4E-BP1. Consistent with previous studies showing that ATF4 can induce the expression of 4E-BP1 (47, 50), but not 4E-BP2 or 4E-BP3, SMAPs only affected the expression of 4E-BP1.

*ATF4* has recently been identified as a target of TFE3 and TFEB, which recognize a coordinated lysosomal expression and regulation (CLEAR) motif (GTCACGTGAC) in the *ATF4* promoter (53). Notably, SMAPs/B56-PP2A promote dephosphorylation of TFE3 and TFEB, which is required for their translocation to the nucleus for activation of transcription. Calcineurin and B55α-PP2A have recently been identified as TFE3/TFEB phosphatases (53, 55), and the use of SMAPs provided the first evidence that B56-PP2A(s) also promote dephosphorylation and translocation of these factors. Our demonstration that knockdown of TFE3/TFEB reduces SMAP/PP2A-induced 4E-BP1 upregulation provides, to our knowledge, the first evidence that these factors regulate 4E-BP1 expression, while suggesting a model in which B56-PP2A activates TFE3/TFEB by dephosphorylation, leading to accumulation of ATF4 for transcriptional upregulation of 4E-BP1 (Figure 14).

Since tumor cells are “addicted” to aberrant eIF4E-dependent protein synthesis, translational control serves as an “Achilles heel” that has emerged as a promising therapeutic target (1, 12). Initial efforts to target this vulnerability focused on inhibitors of upstream 4E-BP1 kinases (17) such as mTOR, with the antitumor activity of mTOR inhibitors largely dependent on their ability to abrogate 4E-BP1 phosphorylation. However, numerous studies have shown that resistance to mTOR inhibitors arises through 4E-BP1 phosphorylation by other mechanisms (3, 16–20). Similarly, ERK-independent 4E-BP1 phosphorylation underlies resistance to anti-BRAF and anti-MEK therapies (16, 21–23). Thus, strategies for suppression of eIF4E-dependent translation that do not rely on inhibition of 4E-BP kinases offer therapeutic promise. Intense efforts are currently focused on targeting the eIF4F complex by downregulating eIF4E using antisense oligonucleotides (ISIS-EIF4ERx) (60), inhibiting the interaction between eIF4E and eIF4G using 4EGI-1, 4E1RCat, and 4E2RCat (61), blocking interaction of eIF4E with the mRNA cap with 4Ei-1 (61), and inhibiting the eIF4A helicase with silvestrol, rocaglamide A, or eFT226 (1, 2, 23). Our discovery that SMAPs block eIF4F assembly through PP2A-dependent activation of 4E-BP1 points to an additional mechanism for restoration of translational control in cancer. Importantly, this approach holds promise not only for tumor suppression, but also for overcoming resistance to small-molecule inhibitors of the ERK and PI3K/AKT/mTOR pathways (16) that are being tested in numerous ongoing clinical trials (ClinicalTrials.gov), a strategy supported by the ability of DT-061 to enhance 4E-BP1 expression and activity in the presence of ERK and mTOR inhibitors.

4E-BPs function as tumor suppressors, and tumorigenesis is critically dependent on inhibition of 4E-BP activity. Thus, many tumors express abundant levels of inactive hyperphosphorylated 4E-BP1, which is available for reactivation and tumor suppression. Other tumors downregulate 4E-BPs or overexpress eIF4E to increase the eIF4E/4E-BP ratio and overwhelm the inhibitory capacity of 4E-BPs. By upregulating and hypophosphorylating 4E-BP1, B56-PP2A activation offers a promising approach for restoration of translational control in tumors harboring different mechanisms of 4E-BP inactivation. Our data further demonstrate that SMAPs/B56-PP2A can activate 4E-BPs in the context of common mechanisms of PP2A inhibition (e.g., overexpression of the endogenous PP2A inhibitors SET [HCT-15, Capan-1] and CIP2A [HCT-15, RKO, MiaPaCa-2] or mutation of Aα subunit at R183 [HCT-116]; refs. 62, 63), indicating that they would be active in a wide range of tumors. The therapeutic potential of SMAPs is highlighted by findings that they induce 4E-BP1–dependent apoptosis in cancer cells and lead to upregulation and activation of 4E-BP1 in tumors in vivo. Thus, B56-PP2A activation is a promising approach for repression of aberrant eIF4E-dependent translation for therapeutic benefit in cancer patients.

## Methods

### Sex as a biological variable.

Male and female athymic nude (*Foxn1^nu^/Foxn1^nu^*) mice (The Jackson Laboratory) were used, and no differences were noted between the sexes.

### Cell culture.

Cells were grown in 5% CO_2_ at 37°C in the following media: HCT-15 (ATCC CCL-225), RKO (ATCC CRL-2577), LoVo (ATCC CCL-229), SW-620 (ATCC CCL-227), SW-480 (ATCC CCL-228), FET (64), HCT-116 (ATCC CCL-247), Capan-1 (ATCC HTB-79), Capan-2 (ATCC HTB-80), Panc-1 (ATCC CRL-1469), BxPC-3 (ATCC CRL-1687), MiaPaCa-2 (ATCC CRM-CRL-1420), Panc89 (RRID:CVCL_4056; from M.A. Hollingsworth, University of Nebraska Medical Center, Omaha, Nebraska), and S2-013 (RRID:CVCL_B280; from M.A. Hollingsworth) cells: RPMI 1640, 10% FBS, 2 mM l-glutamine, and penicillin/streptomycin; Ishikawa cells (RRID:CVCL_2529; from Tim Hui-Ming Huang, Ohio State University, Columbus, Ohio, USA): DMEM, 10% FBS, 1% non-essential amino acids, 5 μg/mL insulin, and penicillin/streptomycin; SNG-M cells (RRID:CVCL_1707, Japanese Collection of Research Bioresources Cell Bank, Osaka, Japan): Ham’s F-12, 10% FBS, and penicillin/streptomycin; H358 cells (ATCC CRL-5807): RPMI 1640, 10% FBS, and penicillin/streptomycin; and IEC-18 cells (ATCC CRL-1589): DMEM, 5% FBS, 4 mM glutamine, 5 μg/mL insulin. PDTOs were generated and cultured as previously described (65, 66). The TM01212 organoid was derived from the PDAC PDX TM01212 (The Jackson Laboratory).

### Drug treatments.

Cells were treated with 20 μM DT-061 (unless otherwise indicated), 10–40 μM DT-1154, 20 μM DT-1310, and 80 μM DT-766. ERK and mTOR inhibitors (Cayman Chemical Co.) were used as follows: 0.1-1.0 μM SCH772984, 0.25–1.0 μM PP242, 10–30 nM INK128, and 10 nM rapamycin. Cal-A (6 nM), OA (120 nM), CHX (200 μM), and ActD (1 μg/mL) were from MilliporeSigma. All drugs were resuspended in DMSO except OA, which was in 50% DMSO/50% ethanol, and rapamycin, which was in 100% ethanol. Equal volumes of vehicle were added to all controls, and final solvent concentrations were ≤0.2%.

### Immunoblotting.

Cells were lysed in 1% SDS, and equal amounts of protein were subjected to immunoblot analysis as we have described (24). Equal loading and even transfer were confirmed by staining with 0.1% fast green (Sigma-Aldrich). Bands were quantified from scans of multiple exposures using ImageJ software (NIH). Dilutions of antibodies for immunoblotting were as follows: 4E-BP1 (Cell Signaling Technology CST-9644, 1:20,000), 4E-BP2 (CST-2845, 1:2,000–1:4,000), 4E-BP3 (Abnova H00008637-M05, 1:500), phospho-64/65 4E-BP1 (CST-9451, 1:500–1:2,000, and Santa Cruz Biotechnology sc-18091-R, 1:500), non-phospho–45/46 4E-BP1 (CST-4923, 1:2,000–1:4,000), GAPDH (CST-5174, 1:60,000), actin (Sigma-Aldrich A2066 and AbClonal AC026, 1:10,000–1:20,000), eIF4E (sc-271480 or sc-9976, 1:2,000–1:4,000), phospho-eIF4E Ser209 (CST-9741, 1:1,000), eIF4G (sc-133155, 1:1,000, or CST-2469, 1:100,000), AKT (CST-9272, 1:3,000), phospho-AKT S473 (CST-4060, 1:20,000), phospho-AKT T308 (CST-2965, 1:1,000–1:8,000), pULK-1 (CST-14202, 1:1,000), total p70S6K (sc-8418, 1:2,000), phospho-p70S6K (AbClonal AP0564, 1:2,000), ERK (CST-9102, 1:2,000), phospho-ERK (CST-9106, 1:2,000), B56α (sc-136045, 1:200), B55α (CST-2290, 1:2,000), ATF4 (Proteintech 10835-1-AP, 1:2,000–1:8,000), TFE3 (CST-14779, 1:2,000), TFEB (CST-4240, 1:1,000), cleaved PARP (CST-5625, 1:1,000), cleaved caspase-3 (CST-9661, 1:500), goat anti-rabbit–HRP (Millipore AP132P, 1:1,000–1:2,000), goat anti-rabbit–HRP (Jackson ImmunoResearch 111-035-144, 1:10,000–1:50,000), and goat anti-mouse–HRP (Bio-Rad 170-6516, 1:3,000). All antibodies were diluted in Tris-buffered saline, 1% Tween-20 (TBS-T), and 5% BSA except for anti-actin, anti-eIF4E, and secondary antibodies, which were diluted in TBS-T with 5% nonfat dried milk.

### RNA interference.

Cells were transfected with 30–100 pmol siRNA using RNAiMAX (Invitrogen). siRNAs from Ambion were: ATF4 siRNA#1, GUGCUGUAGCUGUGUGUUC; ATF4 siRNA#2, CCUGGAAACCAUGCCAGAU; TFEB siRNAs, AGACGAAGGUUCAACAUCA and ACAUCAAUCCUGAAAAUGCAtt; TFE3 siRNAs, GGCGAUUCAACAUUAACGA and AGUUCUACGAGCUCAAAAtt; B55α (rat- and human-specific) Silencer Select Custom siRNA ID s554710, UAAAACUCCUGUCUGUAAUtt; and 4E-BP2 Silencer predesigned siRNA 14090, CAUAAAUGAUUCGAGUUCCtc. siRNAs from Dharmacon were: Human PPP2R5A ON-TARGET plus SMARTpool, L-009352-00-0005; Rat PPP2R5A ON-TARGET plus SMARTpool, L-083443-02-0005. Controls were transfected with 100 pmol ON-TARGET plus nontargeting siRNA (Dharmacon D-001810-01-05).

### Cap affinity assay.

Cells were lysed in 50 mM Tris, pH 7.4, 100 mM NaCl, 1 mM EDTA, and 1% Triton X-100, supplemented with protease and phosphatase inhibitors. After removal of particulate material by centrifugation, lysates (500 mg protein) were incubated with 25–50 μL of 7-methyl-GTP-agarose bead slurry (Jena Biosciences AC-155S) overnight at 4°C. Beads were washed 4 times with lysis buffer, and cap-associated proteins were eluted with 25–50 μL of 2× SDS-PAGE sample buffer.

### Immunoprecipitation.

Cells were lysed in 50 mM Tris-HCl, pH 7.5, 150 mM NaCl,1 mM EDTA, 1% IGEPAL, 10% glycerol, Protease Inhibitor Cocktail, and Phosphatase Inhibitor Cocktails I and II (Sigma-Aldrich). Extracts were incubated with anti-eIF4E antibody (sc-271480, 1–2 μg) overnight at 4°C before 25–50 μL of Protein G PLUS-Agarose slurry (sc-2002) was added for 2 hours at 4°C. Beads were washed 4 times with lysis buffer and extracted with 25–50 μL 2× SDS-PAGE sample buffer.

### 4E-BP1–knockout HCT-116 and MiaPaCa-2 cells.

Knockout cells were generated by the Nebraska Center for Molecular Target Discovery and Development (NCMTDD) Target Validation Core at the University of Nebraska Medical Center. Briefly, cell lines were transfected with the pSpCas9(BB)-2A-GFP (PX458) plasmid expressing guide RNA targeting the *4E-BP1* gene (GCCGAGCACCACCCGGCGAG). GFP-positive clones were selected by flow cytometry and screened for loss of 4E-BP1 expression by immunoblotting.

### Protein synthesis assays.

Protein synthesis was measured using the Click-iT HPG Alexa Fluor 488 Protein Synthesis Assay Kit (Thermo Fisher Scientific). Cells in 96-well plates were treated with vehicle or 20 μM DT-061 for 2.5 or 3.5 hours before incubation in methionine-free RPMI/dialyzed FBS, 20 μM DT-061 (or vehicle), and 50 μM l-homopropargylglycine. After 30 minutes, l-homopropargylglycine–containing proteins were labeled with Alexa Fluor 488, and nuclei were stained with NuclearMask Blue (Thermo Fisher Scientific) according to the manufacturer’s instructions. Fluorescence was measured using a SpectraMax M5e plate reader (Molecular Devices). Background Alexa Fluor fluorescence was determined by labeling of cells in the presence of 100 μg/mL CHX, and Alexa Fluor readings above background were divided by the NuclearMask signal to normalize for cell number.

### Cap-dependent translation assay.

MiaPaCa2 cells were stably transfected with the pcDNA3-RLUC-POLIRES-FLUC plasmid (Addgene 45642) (44). Pooled stable transfectants were plated at 1.67 × 10^5^ cells per well in 12-well plates and treated with 20 μM DT-061 or vehicle for 6 hours before firefly and Renilla luciferase activity was quantified using the Dual Luciferase Reporter Assay System (Promega). Relative cap-dependent translation was derived by normalization of Renilla luciferase activity to firefly luciferase activity for each sample.

### RT-qPCR and qPCR.

RNA was isolated using Trizol (Invitrogen), and RT-qPCR was performed using the 1-Step Brilliant II SYBR Green QRT-PCR Master Mix kit (Agilent) on a Bio-Rad C1000 Thermal Cycler CFX96 Real-Time System. Relative mRNA concentrations were determined from standard curves using Bio-Rad CFX Manager Software, v3.1, and mRNA levels were normalized to 18S rRNA. Primers were: human 4E-BP1, GGAGTGTCGGAACTCACCTG and ACACGATGGCT‌GGTG‌CT‌TTA; human ATF4, TTCTCCAGCGACAAGGCTAAGG and CTCCAACATCCAATCTGTCCCG; human/rat 18S rRNA, CATTGGAGGGCAAGTCTGGTG and CTCCCA‌AGC‌TCCA‌ACTA‌C‌GAG; human GAPDH, TGAAGGTCGGAGTCAACGGA and CCATTGATGACA‌AGC‌TT‌CCCG.

### Nascent RNA analysis.

Analysis used the Click-iT Nascent RNA Capture Kit (Invitrogen). In brief, 0.5 mM 5-ethynyl uridine (EU) was added for the final 1 hour of drug/vehicle treatment, and RNA was purified using Trizol. EU-labeled RNA was biotinylated and isolated using streptavidin beads. cDNA was generated from bead-bound RNA using the iScript cDNA Synthesis Kit and analyzed by qPCR using iTaq Universal SYBR Green Supermix (Bio-Rad). Normalization to nascent GAPDH mRNA and calculation of relative levels used the ΔΔCt method.

### Proteomic analysis.

Frozen primary and metastatic PDAC and tumor-adjacent tissues, sample preparation, and demographics were as we described (67). Mass spectrometry data files have been published, and the raw data can be found in the ProteomeXchange Consortium via the PRIDE repository with project accessions PXD012173 and PXD015492 (67).

### Phosphoproteomic analysis.

Samples were lysed in 2% SDS and subjected to 2-step Lys-C/trypsin proteolytic cleavage and phosphopeptide enrichment using TiO_2_ spin tips (Thermo Fisher Scientific). Liquid chromatography–tandem mass spectrometry (LC-MS/MS) on an Orbitrap ProVelos Elite MS system (Thermo Fisher Scientific) with a nanoAcquity ultra-HPLC system (Waters) and phosphopeptide quantification were as we described (34).

### Immunofluorescence analysis.

Cells on glass coverslips were treated with vehicle or 20 μM DT-061 for 1 hour, fixed in 4% formaldehyde/PBS, permeabilized with 0.2% Triton X-100, and stained with anti-TFE3 (Sigma-Aldrich HPA023881, 1:100) and Alexa Fluor 488–conjugated donkey anti-rabbit antibody (1:1,000). Nuclei were stained with Hoechst 33258. Images were obtained using a Zeiss Axio Observer.Z1 microscope with an Axiocam 208 camera.

### Cell cycle analysis.

Cells were fixed in 70% ethanol and stained with propidium iodide as previously described (68). Propidium iodide fluorescence was detected using a BD FACSCalibur 3 and analyzed using Verity ModFit software.

### In vivo studies.

For PDX TM01212 (The Jackson Laboratory), tissue was suspended in Matrigel (Corning) and implanted subcutaneously into the flank of nude mice. For SW-620 xenografts, nude mice were injected subcutaneously with 5 × 10^6^ to 6 × 10^6^ cells in sterile 50% PBS/50% Matrigel or Cultrex Type 2 (R&D Systems). For analysis of effects on tumor growth, mice were randomized when tumors reached approximately 50–100 mm^3^ and treated twice daily with vehicle (10% dimethylacetamide, 10% Kolliphor HS 15) (Oakwood Chemicals) or DT-061 (15 or 50 mg/kg) by oral gavage. Tumor volume was estimated using calipers (width^2^ × length × 0.5). Mice were sacrificed after 21 or 28 days, and tumors were removed and weighed. For assessment of effects on 4E-BP1, mice were treated as above and sacrificed at 3 hours (15 mg/kg) or 2, 12, or 24 hours (50 mg/kg). Tumors were removed, extracted using T-PER Tissue Protein Extraction Reagent (Thermo Fisher Scientific) with protease and phosphatase inhibitors, and analyzed by immunoblotting.

### Analysis of publicly available datasets.

*4E-BP1* mRNA and protein data from TCGA and CPTAC were analyzed using the University of Alabama at Birmingham UALCAN interactive web portal (http://ualcan.path.uab.edu/analysis.html) (69).

### Statistics.

Numerical data are presented as means ± SEM. For presentation, contrast and brightness of scanned images were adjusted using Adobe Photoshop or Microsoft PowerPoint software; all adjustments were made equally across the entire blot, and no lanes were treated differently from the rest of the blot. Dashed lines indicate where lanes have been rearranged for clarity. Statistical analysis was performed using Microsoft Excel and involved 1-sided or 2-sided Student’s *t* test with Holm-Bonferroni adjustment for multiple comparisons as appropriate (log values were used in analysis of normalized fold change data). *P* less than 0.05 was considered significant. Generation of graphs used GraphPad Prism, and figures were assembled and annotated in Microsoft PowerPoint (without image compression).

### Study approval.

PDTOs Q23.28 and Q20.70 were obtained from the NCMTDD Organoid Core and were designated as non–human subjects research by the University of Nebraska Medical Center (UNMC) IRB. Human samples used for LC-MS/MS analysis were from deceased patients and these studies are not human subject research according to 45 CFR 46.102(e1) and do not require IRB oversight. Use of PDTO PPTO69 was approved by the University of Washington IRB and is covered by protocol 2015P002328. Animal studies were approved by the IACUCs at UNMC, the University of Michigan and the Fred Hutchinson Cancer Center, and are covered by protocols 18-177-01-FC (UNMC), PRO00010404 (University of Michigan), and 50935-200016 (Fred Hutchinson Cancer Center).

### Data availability.

All data described are contained within the article and its Supplemental Figures and the Supporting Data Values file. Mass spectrometry data files have been published and the raw mass spectrometry proteomics data have been deposited to the ProteomeXchange Consortium (http://proteomecentral.proteomexchange.org) via the PRIDE partner repository with the dataset identifiers PXD012173 and PXD015492 (67).

## Author contributions

MAL and KAJ were responsible for conceptualization, investigation, methodology, data curation, formal analysis, original draft preparation, reviewing and editing. ARB was responsible for conceptualization, investigation, methodology, data curation, formal analysis, original draft preparation, reviewing and editing, supervision, and visualization. CMO was responsible for investigation, data curation, and formal analysis. SP, SD, NY, TMR, AS, and MG were responsible for investigation. RAA and AD were responsible for conceptualization. SK was responsible for conceptualization and investigation. NTW was responsible for conceptualization, investigation, data curation, and formal analysis. GN was responsible for conceptualization, investigation, formal analysis, reviewing and editing, and funding acquisition. JDB was responsible for conceptualization, investigation, methodology, formal analysis, original draft preparation, supervision, visualization, reviewing and editing, and funding acquisition. MAL and KAJ share first authorship for the study. MAL is listed first because she initiated the study and generated the data that formed the basis of the manuscript.

## Supplementary Material

Supplemental data

Unedited blot and gel images

Supporting data values

## Version 1 01/27/2025

In-Press Preview

## Version 2 02/17/2025

Electronic publication
