# Supplementary material for: Small-molecule modulators of B56-PP2A restore 4E-BP function to suppress eIF4E-dependent translation in cancer cells
Source: J Clin Invest. 2025 Jan 27;135(4):e176093. doi: 10.1172/JCI176093 (PMC11827888; doi:10.1172/JCI176093)
Supplement: Supplemental data [file jci-135-176093-s263.pdf]

Supplemental Figure S1

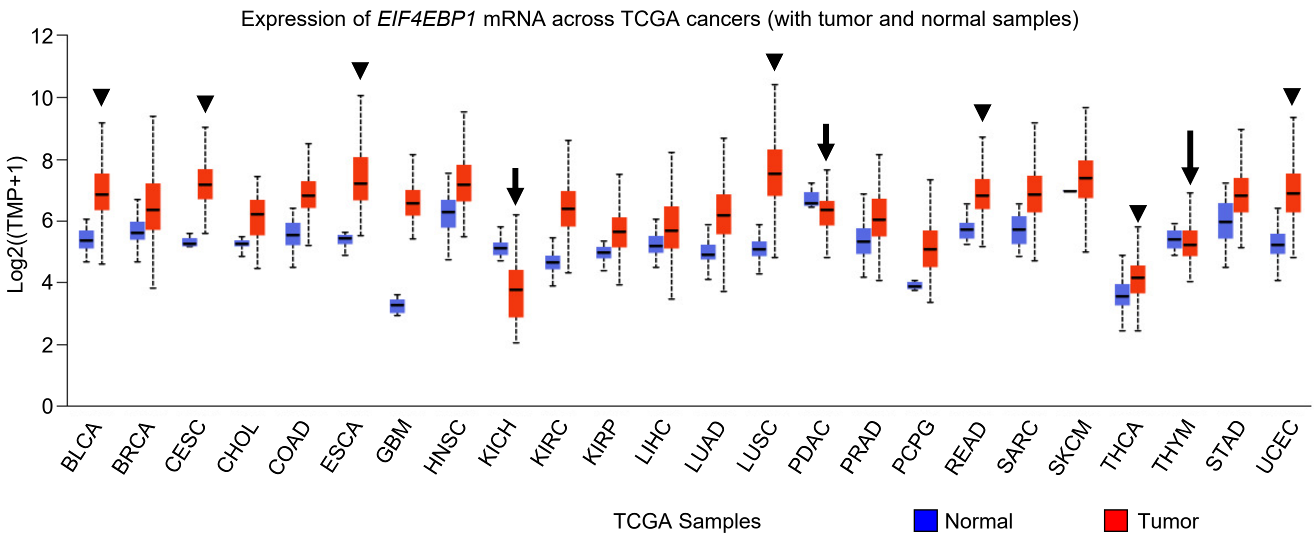

Figure S1. Expression 4E-BP1 mRNA in tumors. TCGA RNAseq data for the expression of *4E-BP1* (*EIF4EBP1*) mRNA in the indicated normal and tumor tissues analyzed using the UALCAN portal. The arrows and arrowheads indicate tumor types in which *4E-BP1* mRNA is downregulated or upregulated relative to normal tissue, respectively.

Supplemental Figure S2

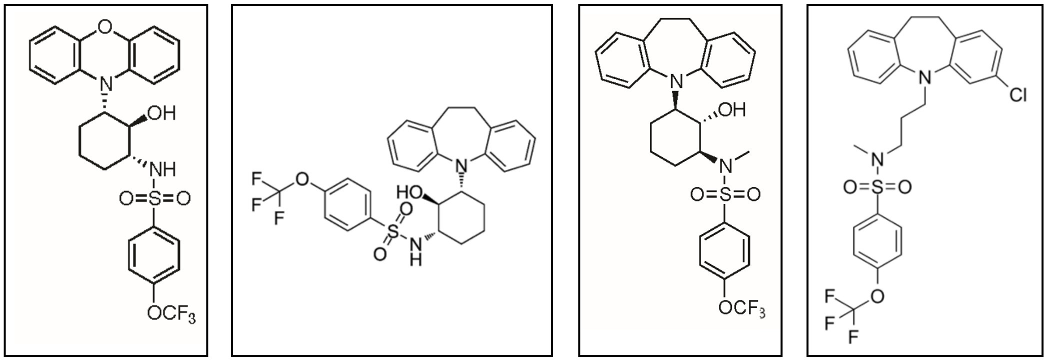

DT-061 DT-1154 DT-1310 DT-766

Figure S2. Chemical structure of DT-061, DT-1154, DT-1310, and DT-766.

Supplemental Figure S3

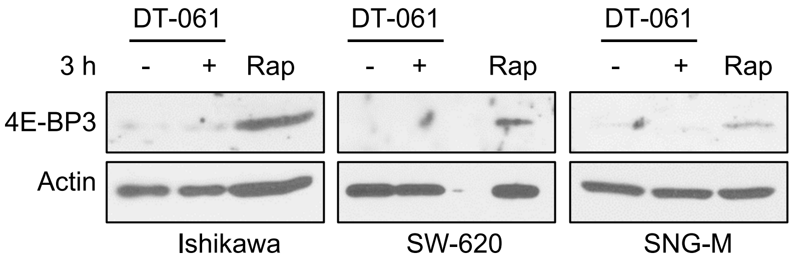

Figure S3. SMAPs do not induce upregulation of 4E-BP3. The indicated cells were treated with vehicle or 20 μM DT-061 for 3 h (Ishikawa, SW-620) or 5 h (SNG-M) and analyzed by immunoblotting. Rap: Extracts of MiaPaCa-2 cells treated with 10 nM rapamycin for 24 h to upregulate 4E-BP3 (38) used as a positive control.

Supplemental Figure S4

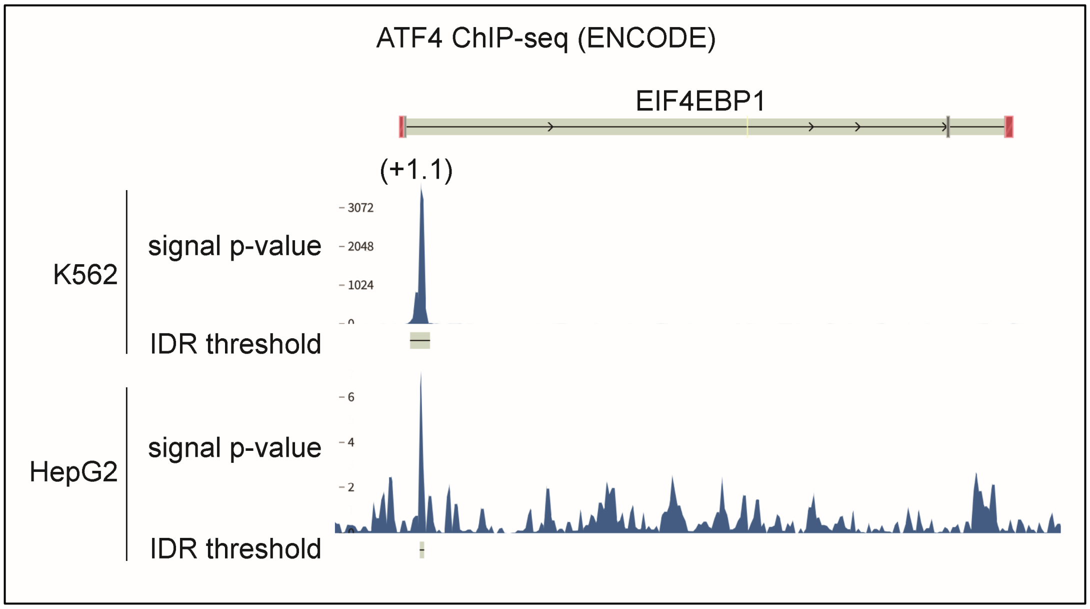

Figure S4. ATF4 ChIP-seq data in K562 and HepG2 cells from the ENCODE database. The number in parentheses above the peak represents its distance relative to the TSS.

Supplemental Figure S5

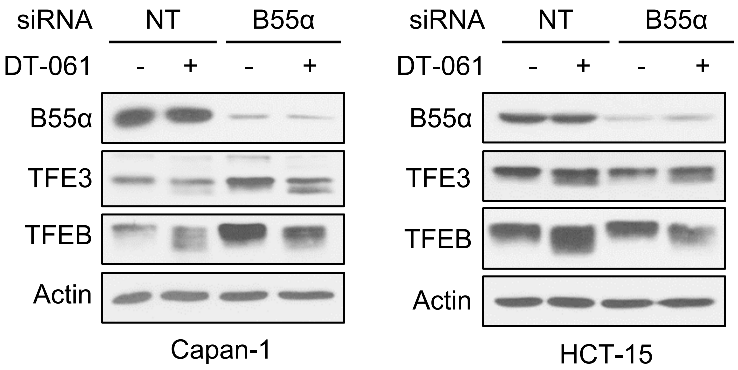

Figure S5. B55α-PP2A does not mediate the effects of SMAPs on TFE3 and TFEB. Cell lines were transfected with non-targeting (NT) or B55α-targeting siRNA 72 h prior to treatment with vehicle or 20 μM DT-061 for 1 h, and expression and electrophoretic mobility of the indicated proteins were determined by immunoblotting.

Supplemental Figure S6

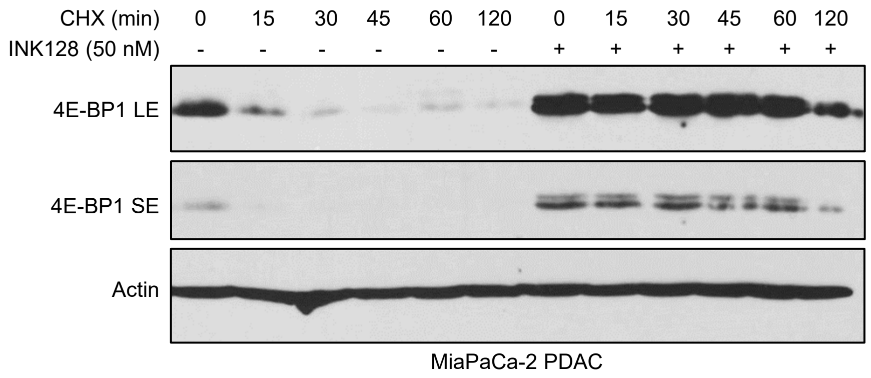

Figure S6. mTOR inhibition with INK128 leads to stabilization of 4E-BP1 protein. MiaPaCa-2 cells were treated with INK128 20 minutes prior to addition of 100 µg/ml CHX and cells were processed for immunoblotting at the indicated times. LE: longer exposure; SE: shorter exposure.
