# Supplementary material for: Small-molecule modulators of B56-PP2A restore 4E-BP function to suppress eIF4E-dependent translation in cancer cells
Source: J Clin Invest. 2025 Jan 27;135(4):e176093. doi: 10.1172/JCI176093 (PMC11827888; doi:10.1172/JCI176093)

Full unedited blots for Figure 1C

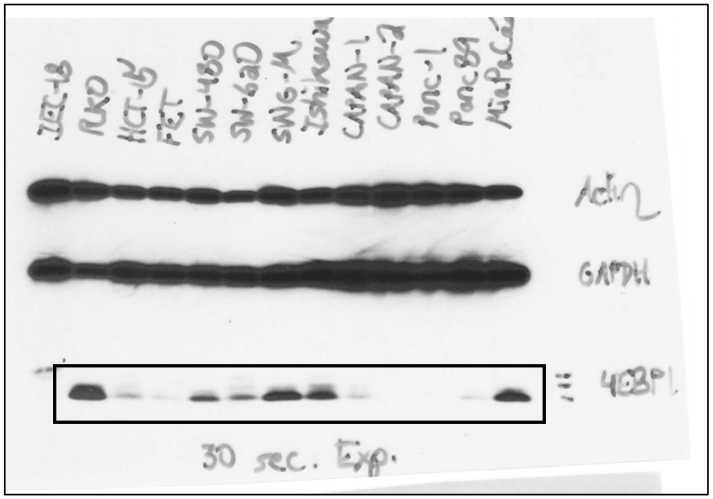

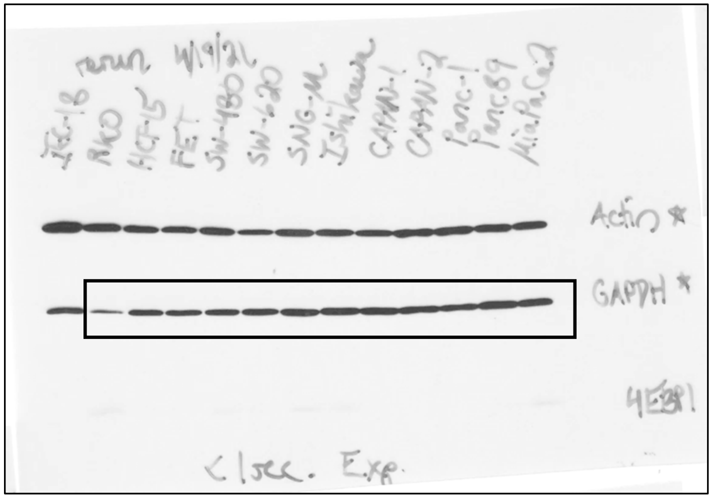

Full unedited blots for Figure 2A-C

2A

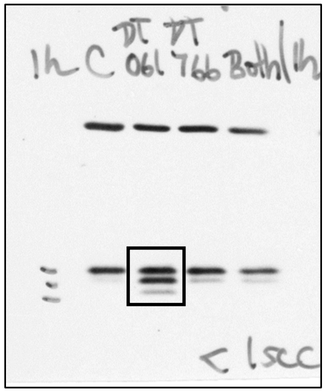

2B

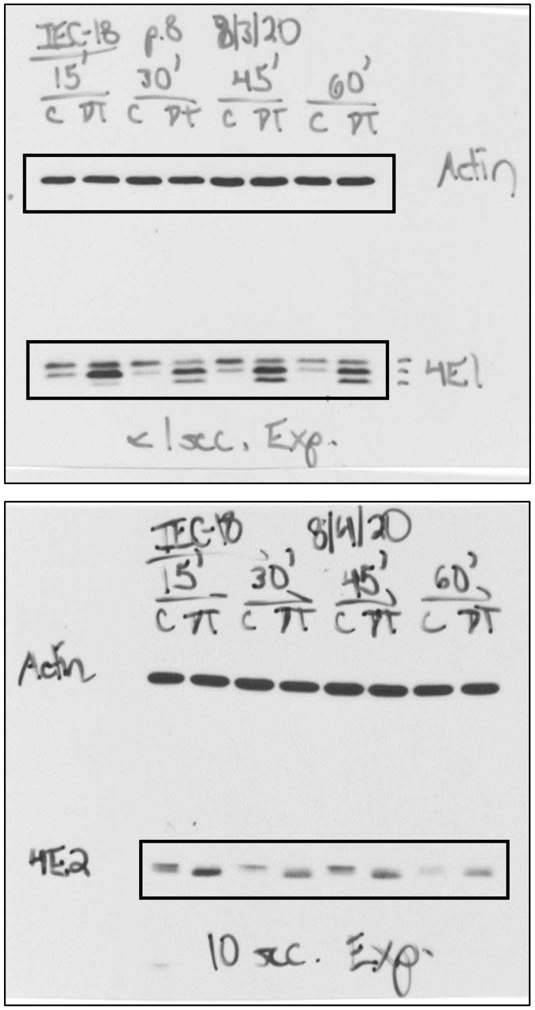

2C

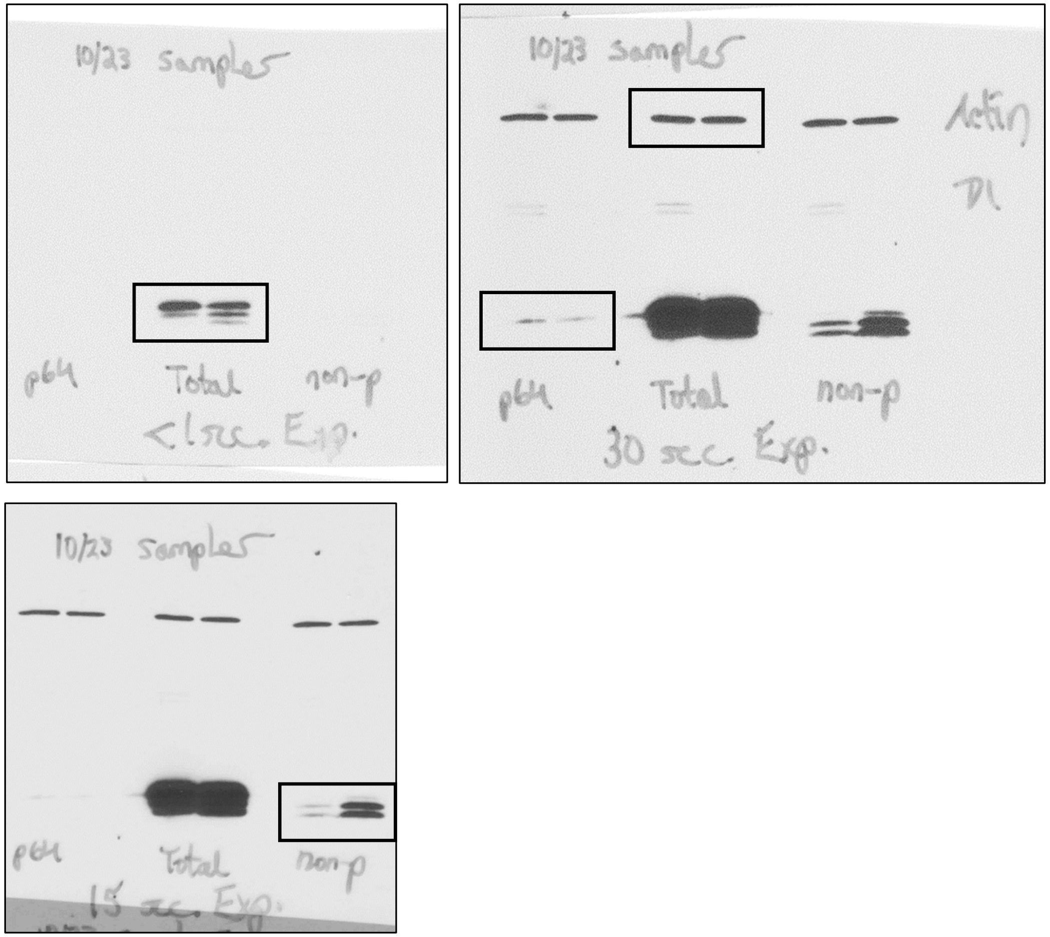

Full unedited blots for Figure 2D-E

2D

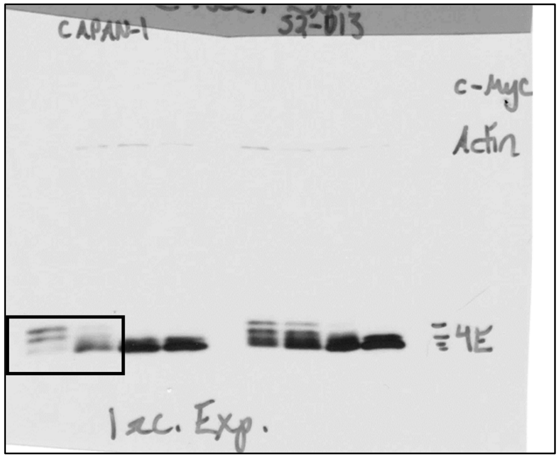

Ishikawa

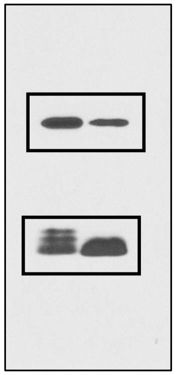

Loading control

4E-BP1

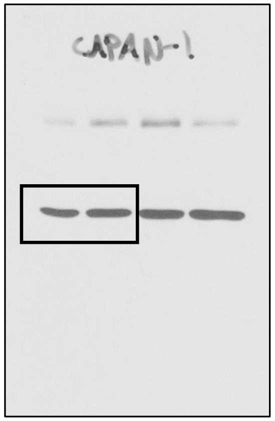

Actin

2E

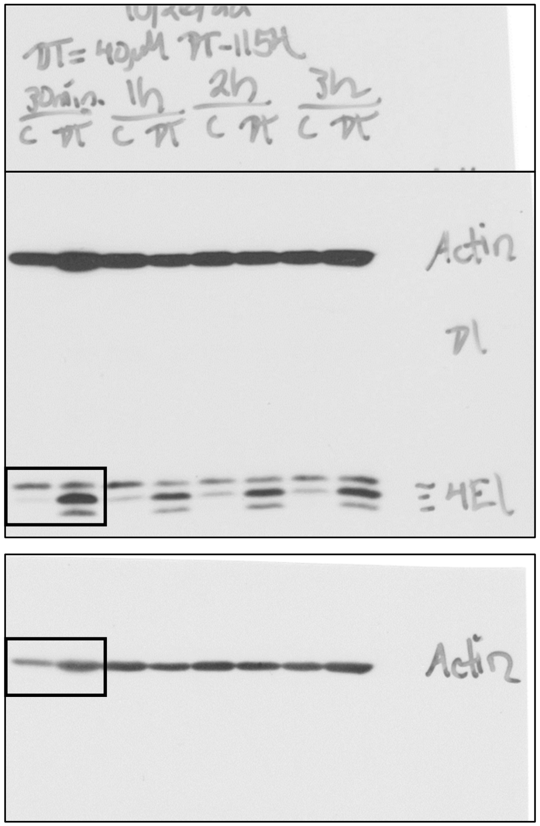

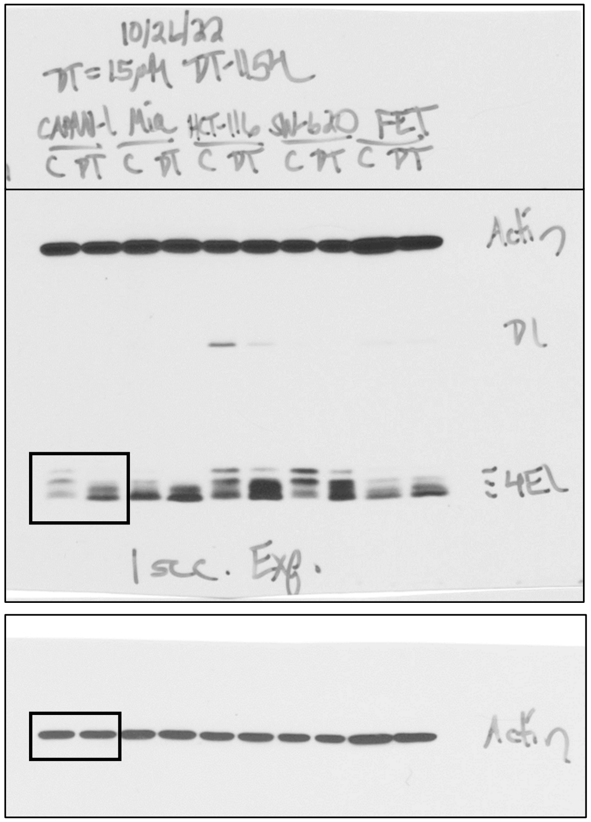

Full unedited blots for Figure 2F

2F

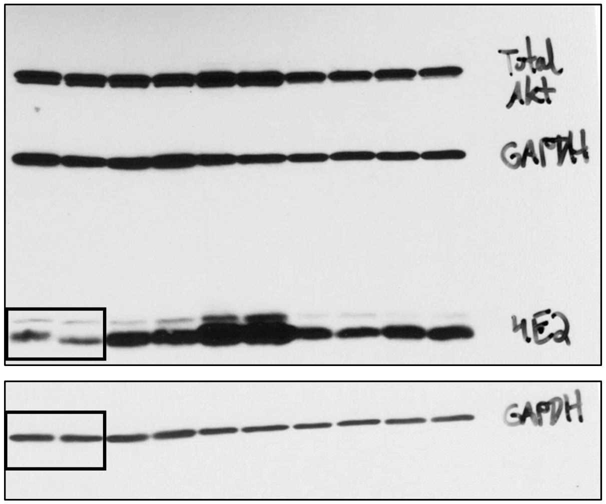

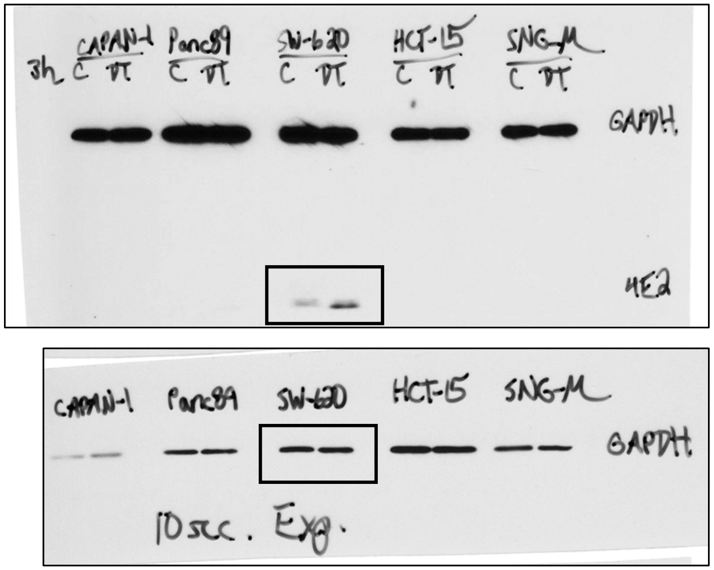

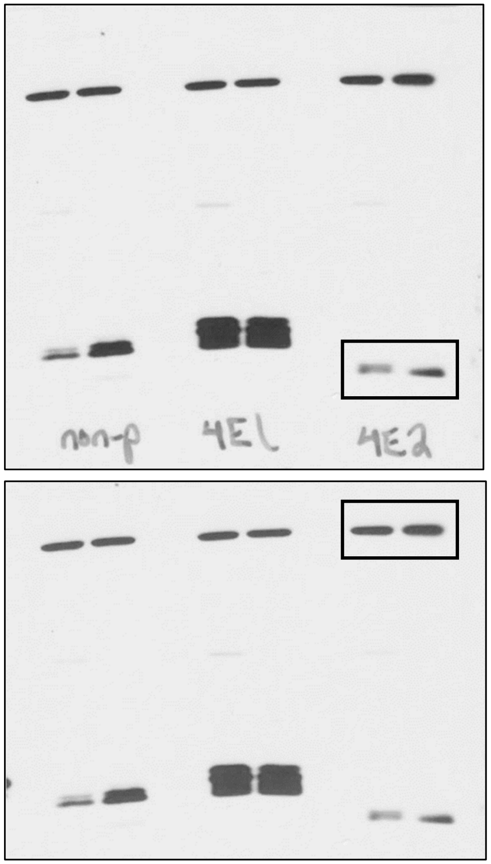

Full unedited blots for Figure 2G

2G

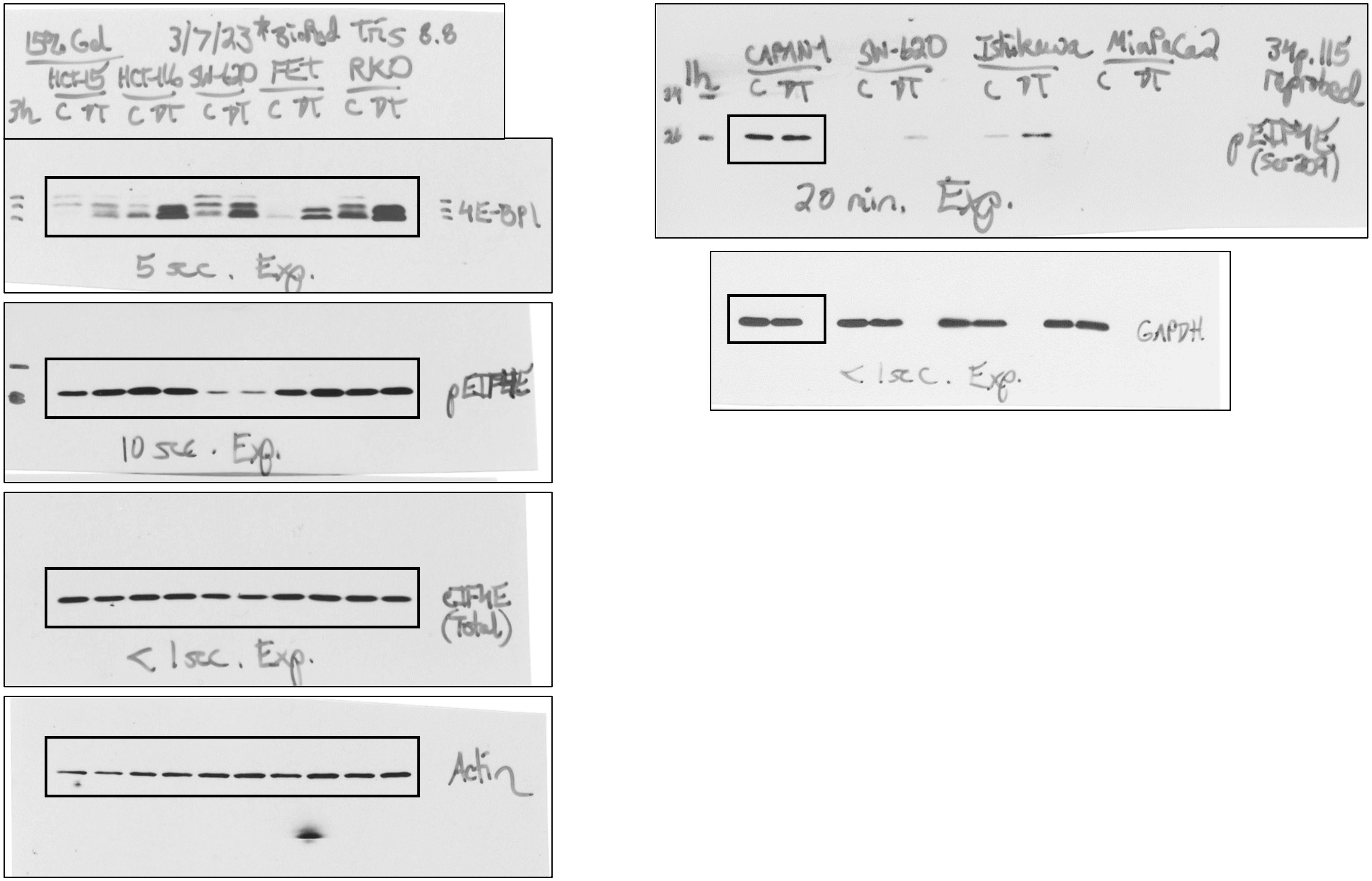

Full unedited blots for Figure 2H

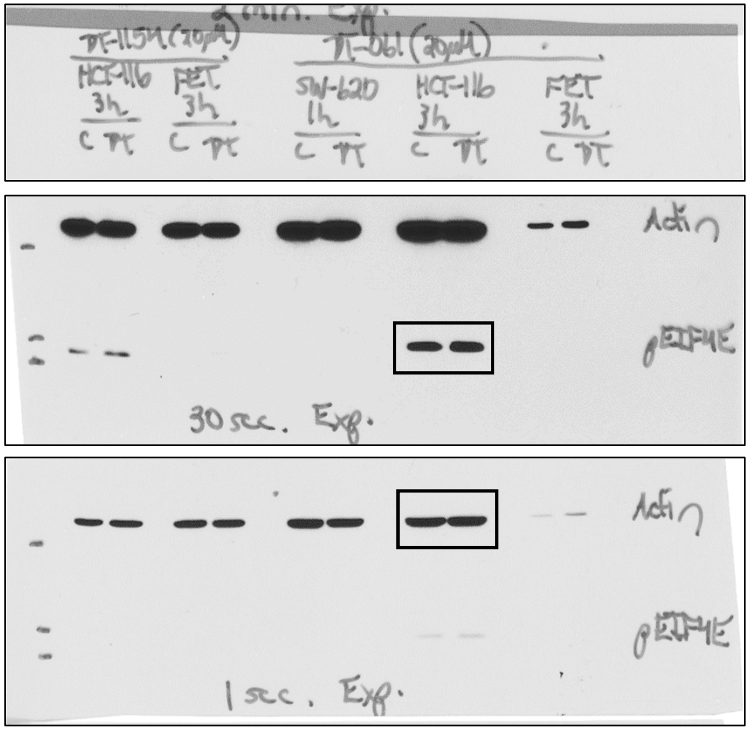

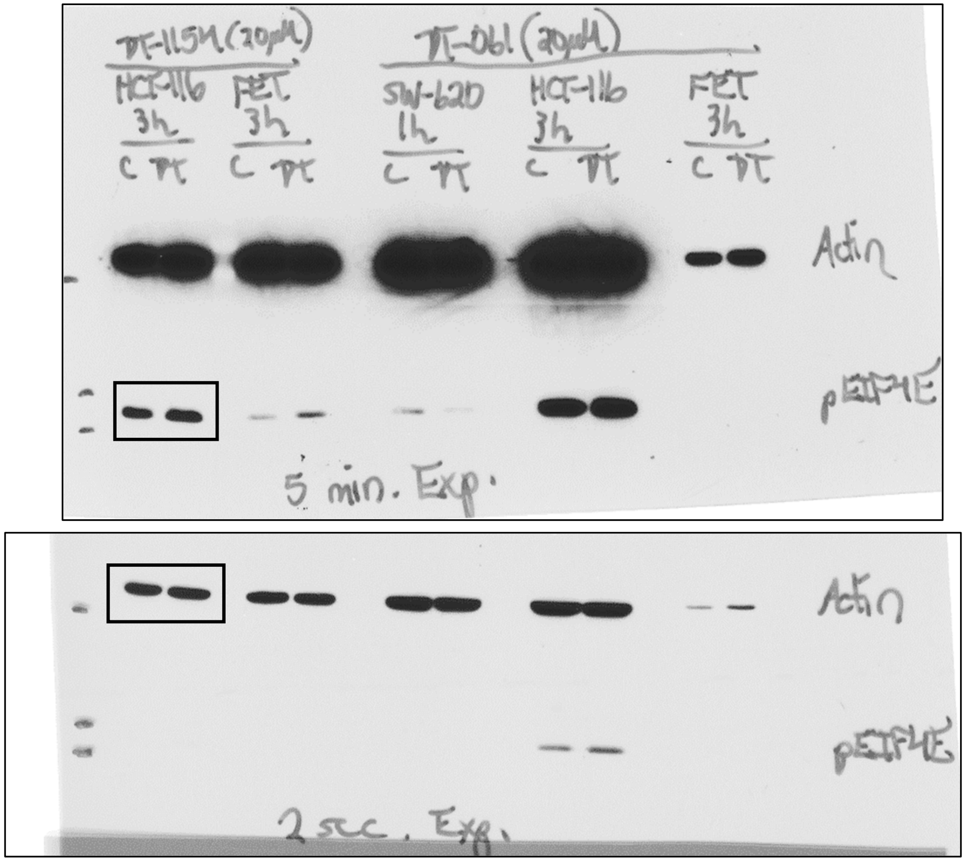

Full unedited blots for Figure 3A-C

3A

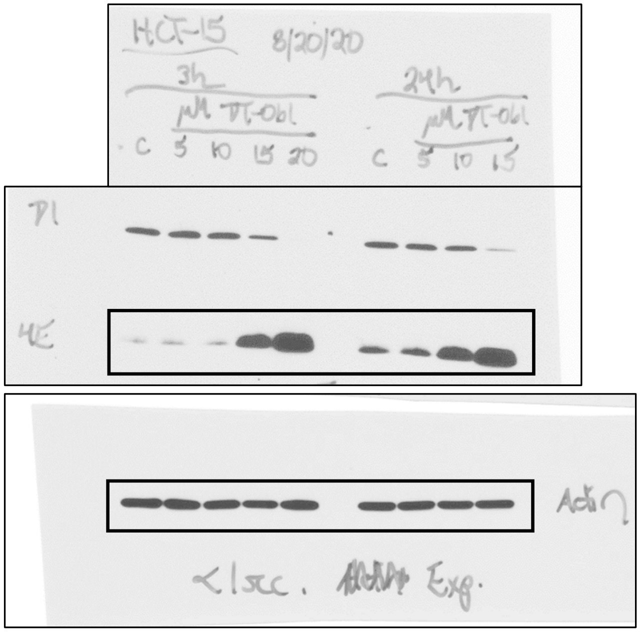

3B

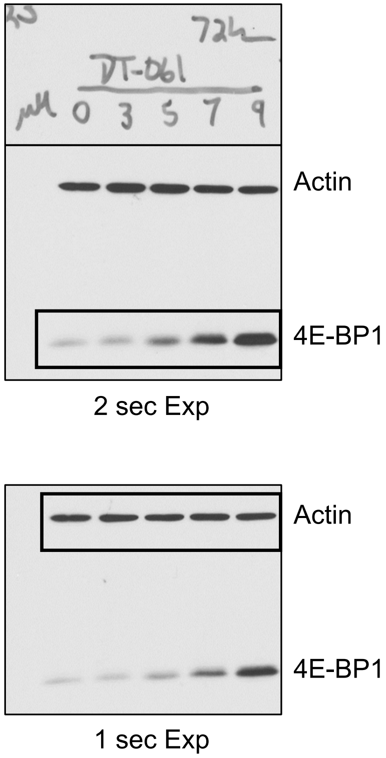

3C

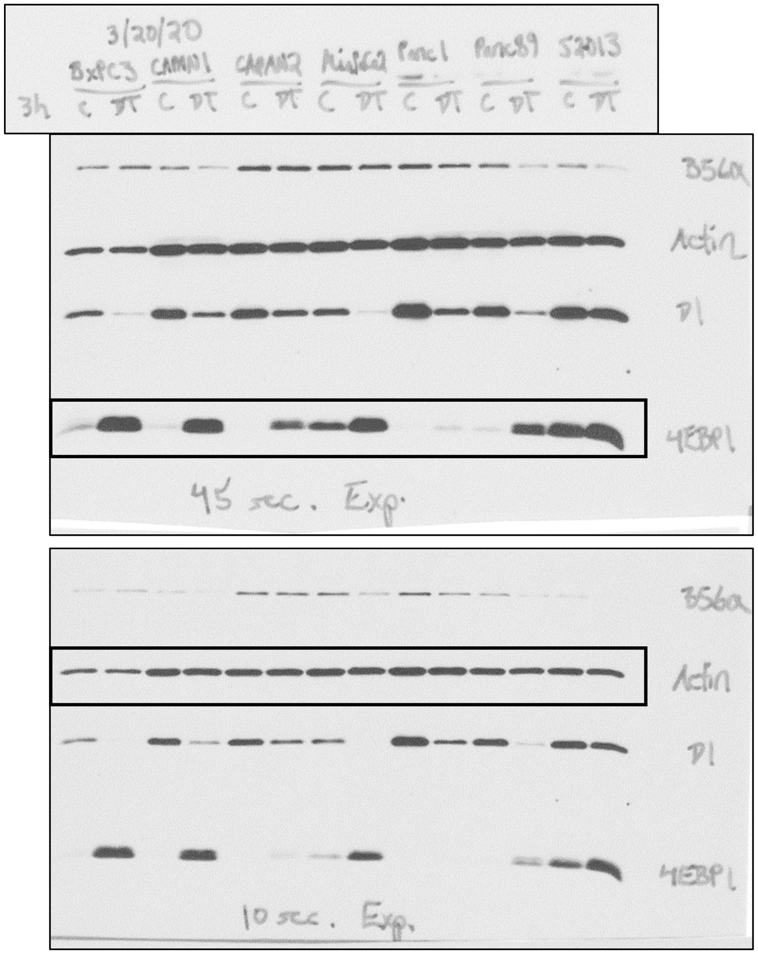

Full unedited blots for Figure 3D

3D

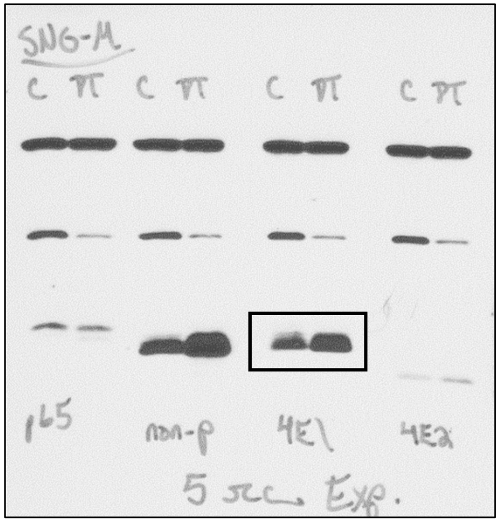

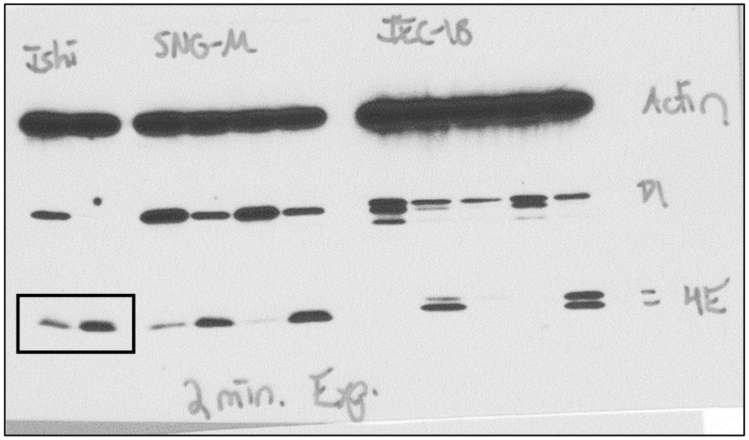

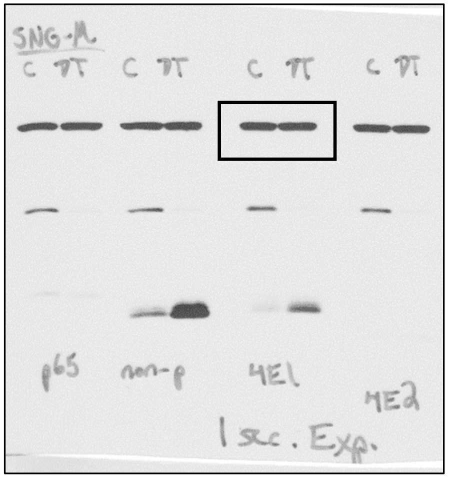

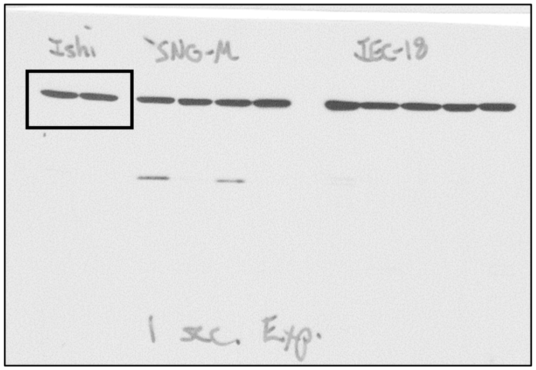

Full unedited blots for Figure 3E

3E

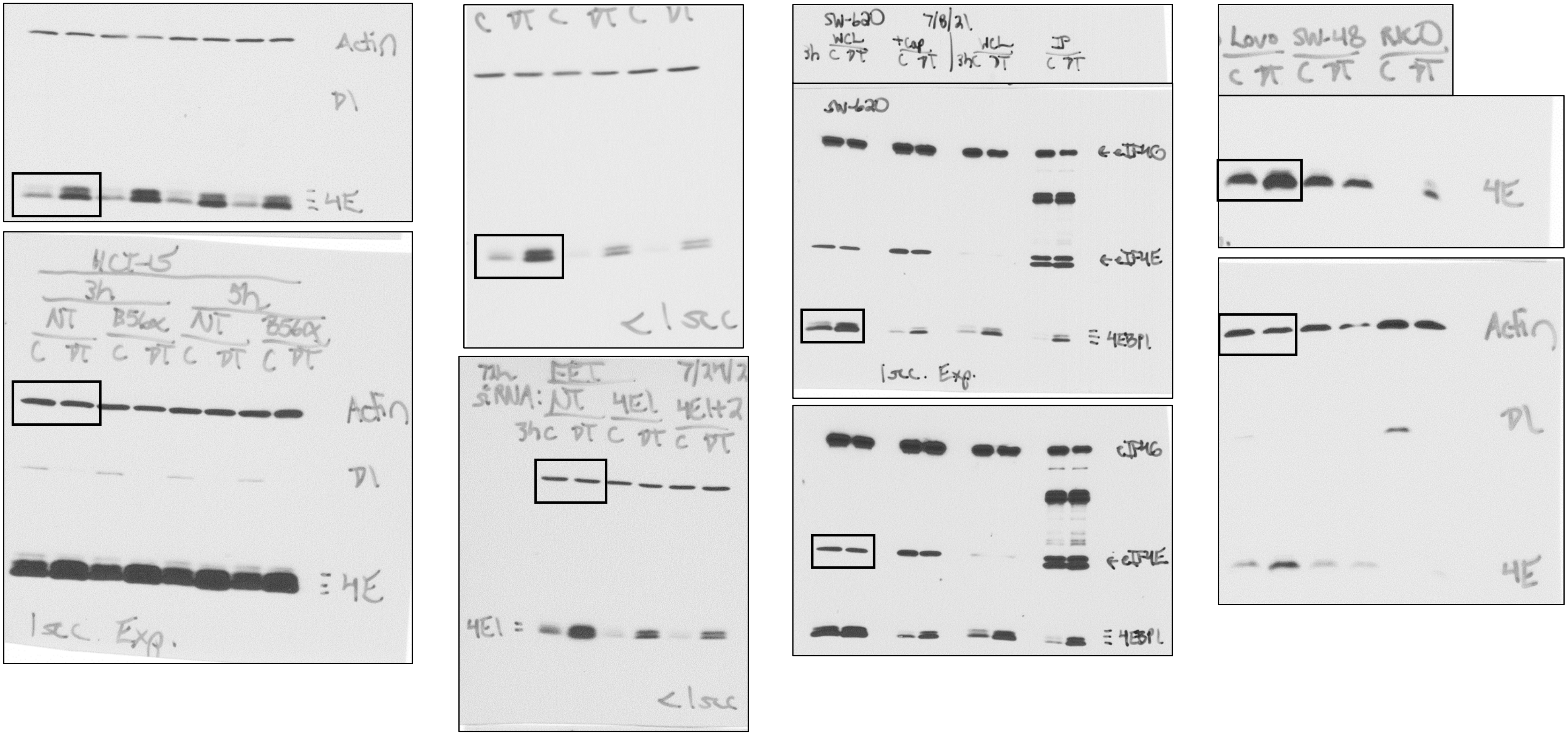

Full unedited blots for Figure 3F-G

3F

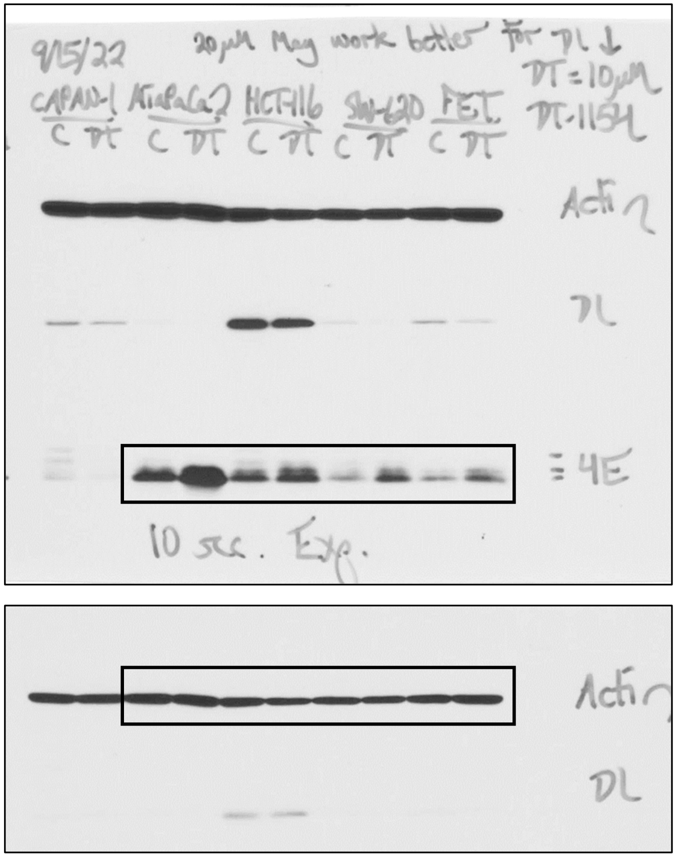

3G

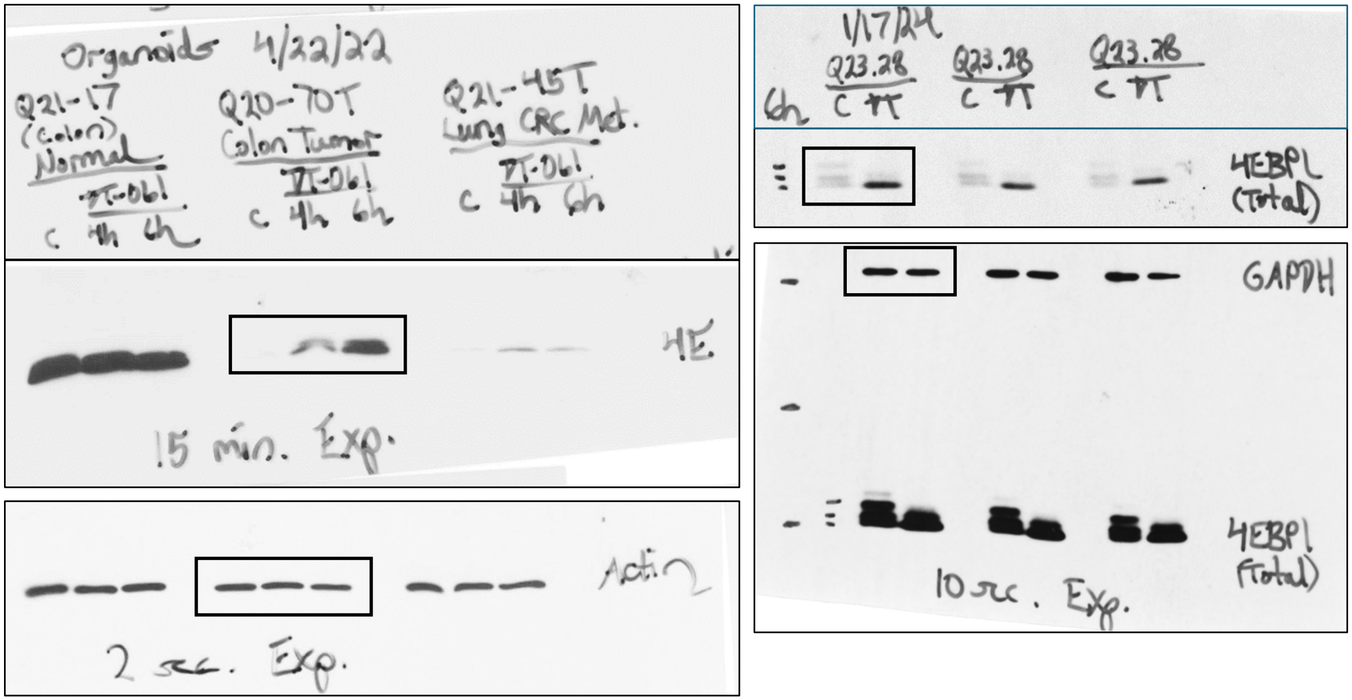

Full unedited blots for Figure 3H

3H

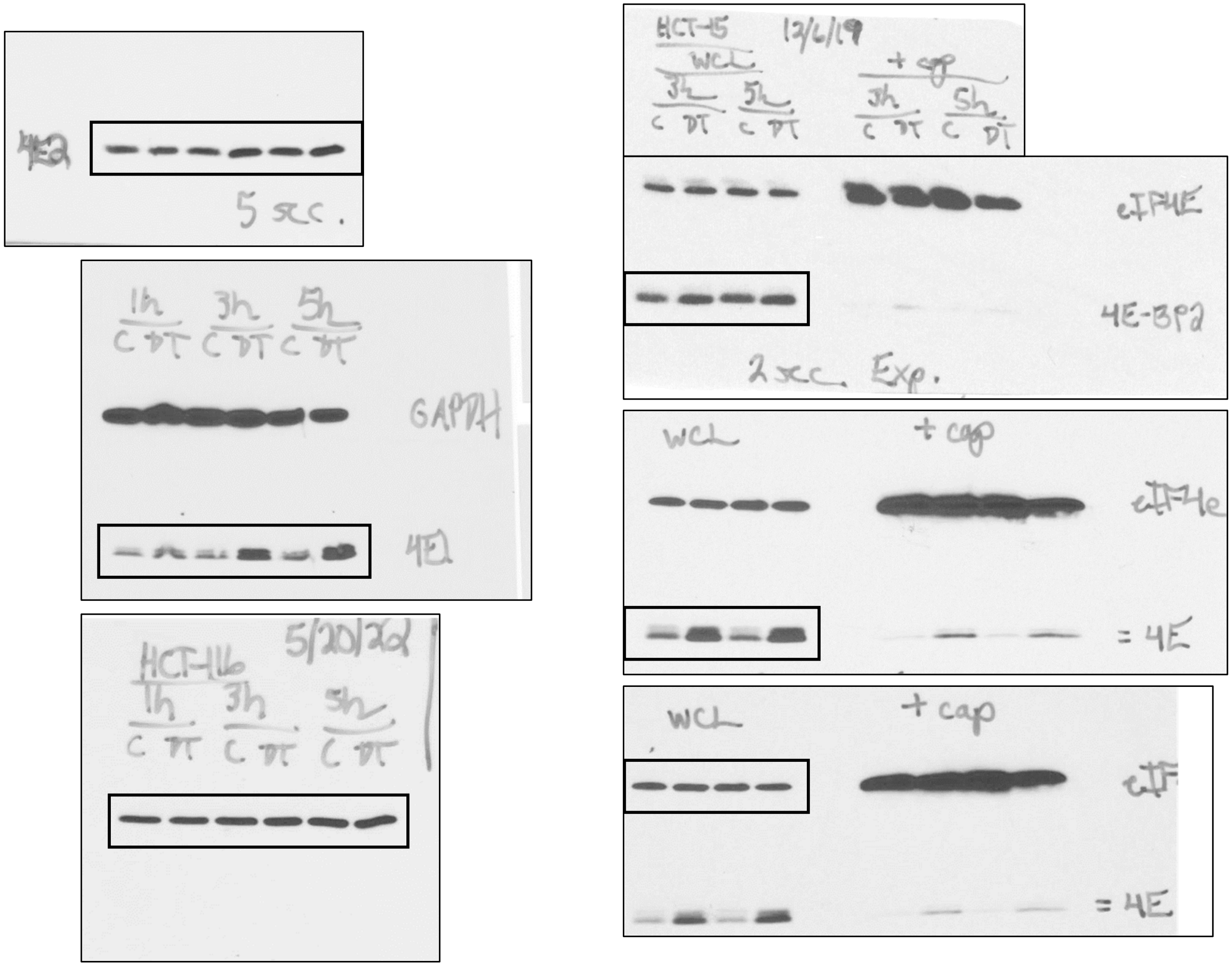

Full unedited blots for Figure 3I

3I

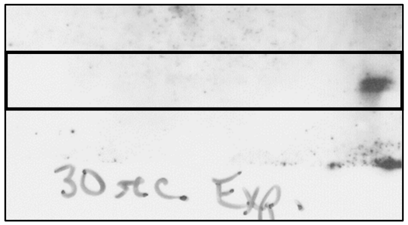

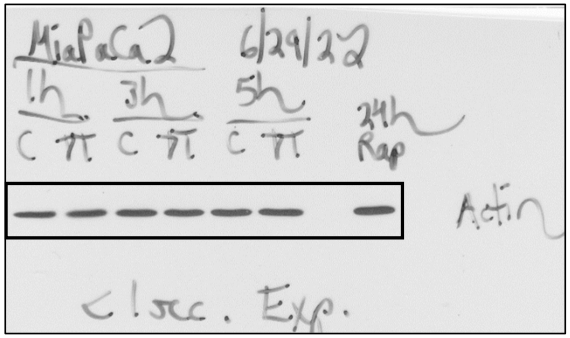

Full unedited blots for Figure 4A-B

4A

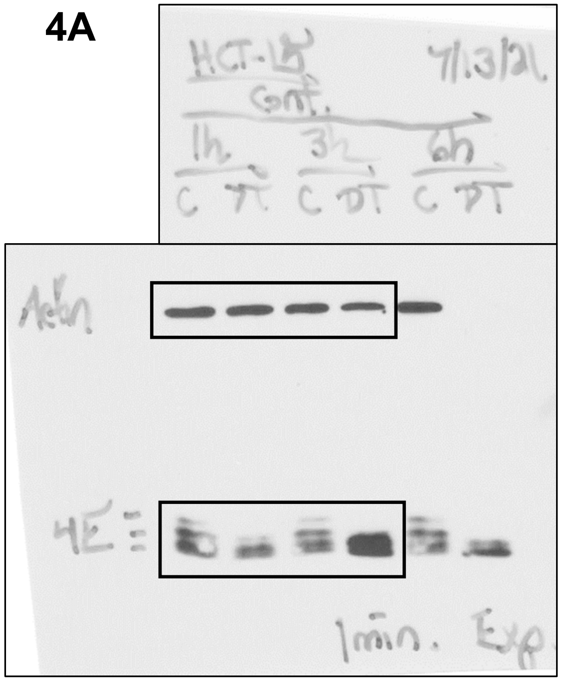

4B

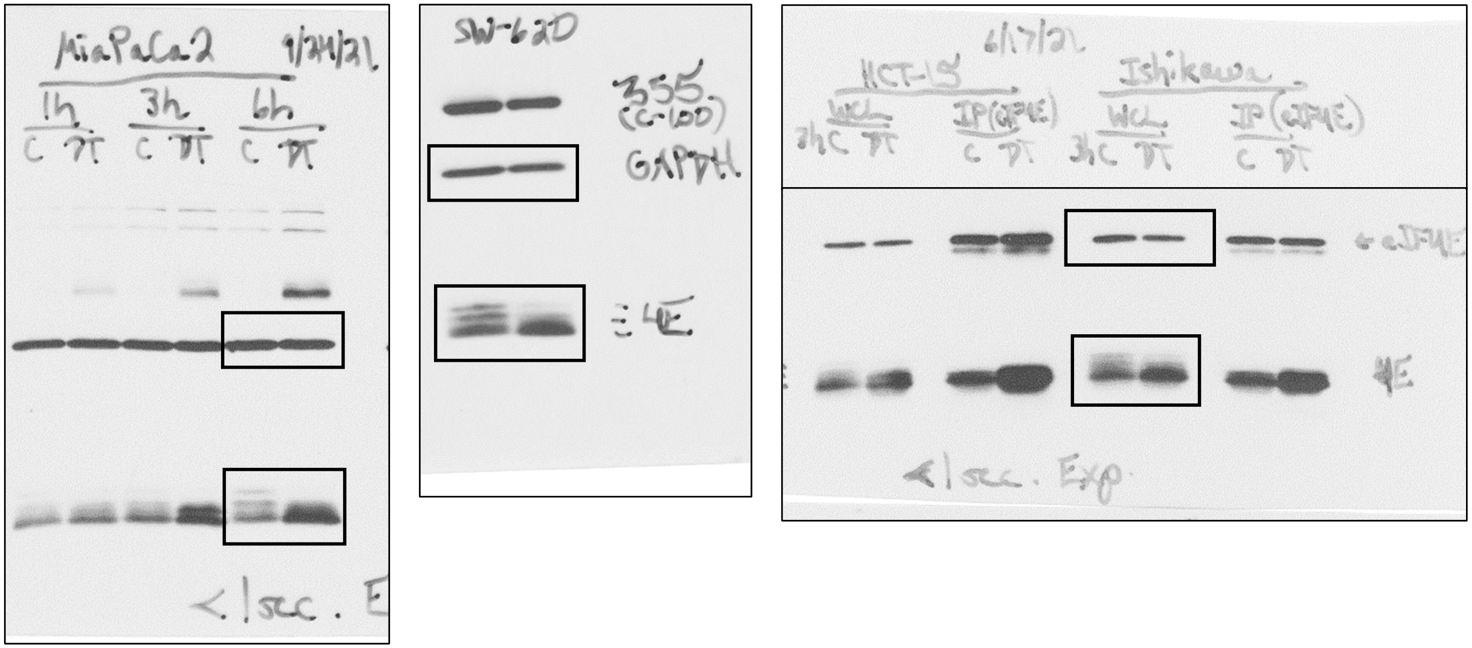

Full unedited blots for Figure 4C

4C

Capan-1 (3 h)

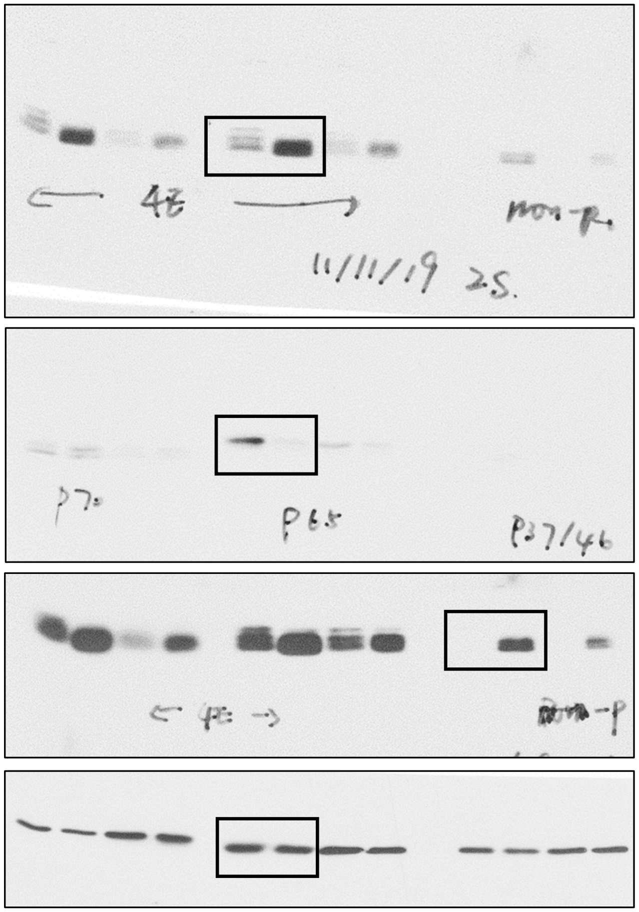

Panc89 (3 h)

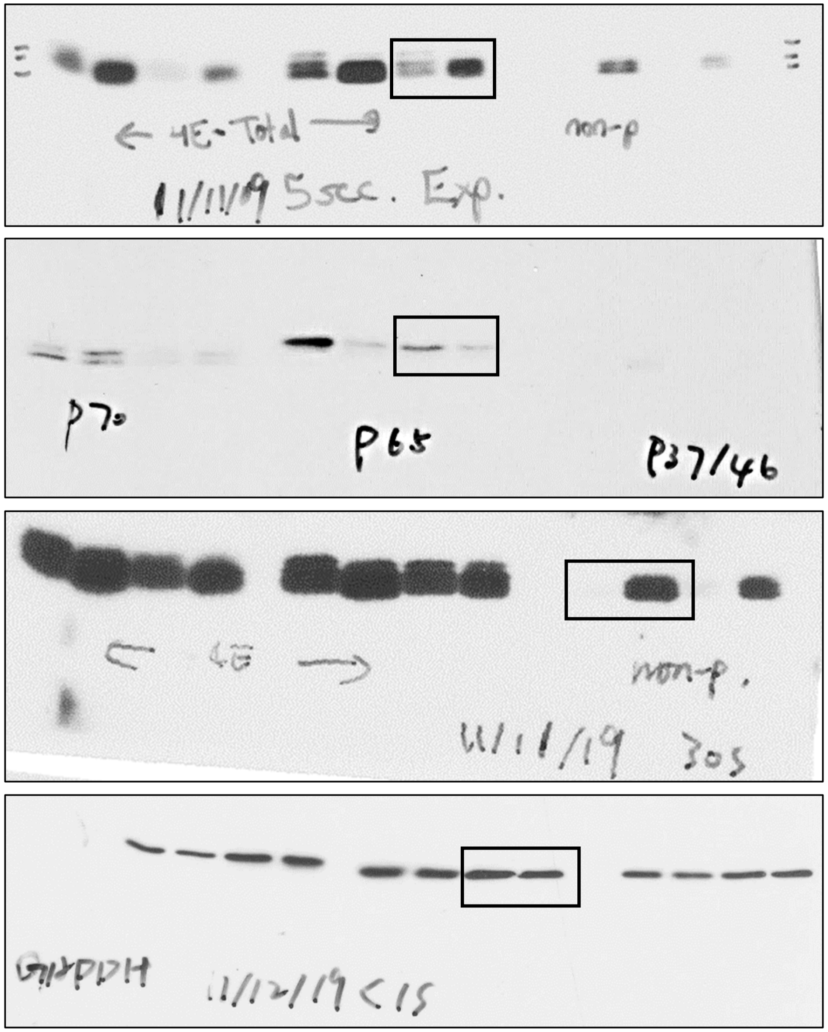

Full unedited blots for Figure 4C (continued)

4C

HCT-15 (3.5 h)

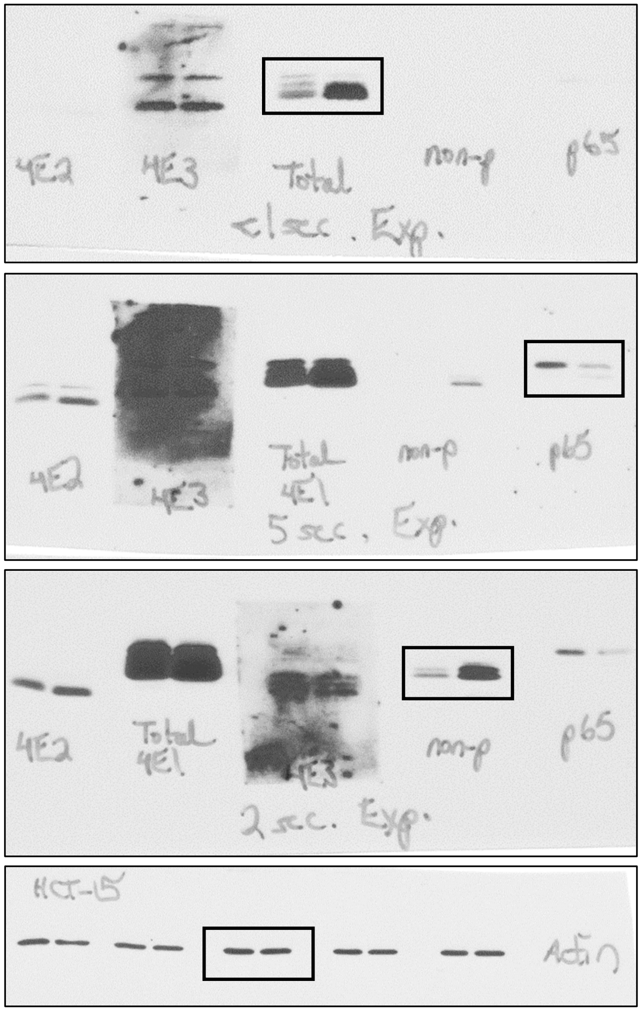

SW-620 (3 h)

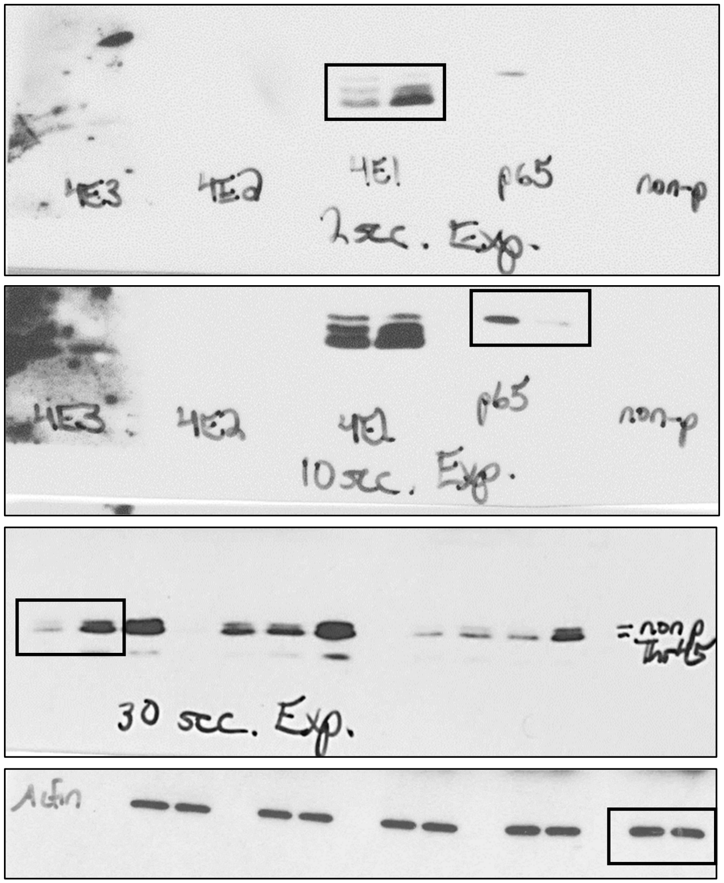

Full unedited blots for Figure 4C (continued)

4C

SNG-M (2 h)

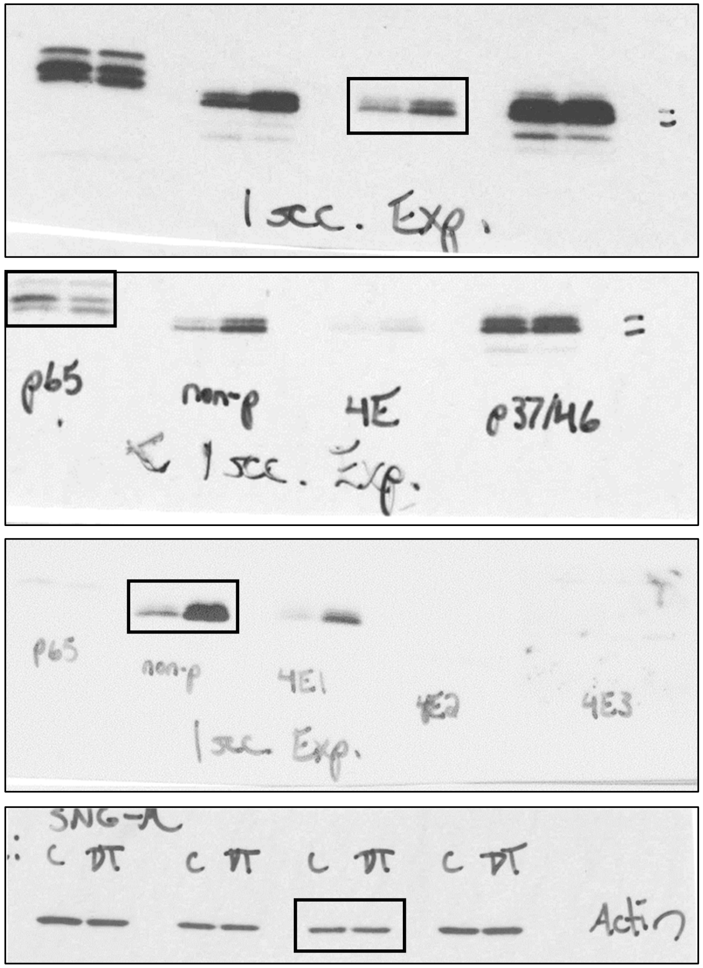

Full unedited blots for Figure 5A

5A

IEC-18

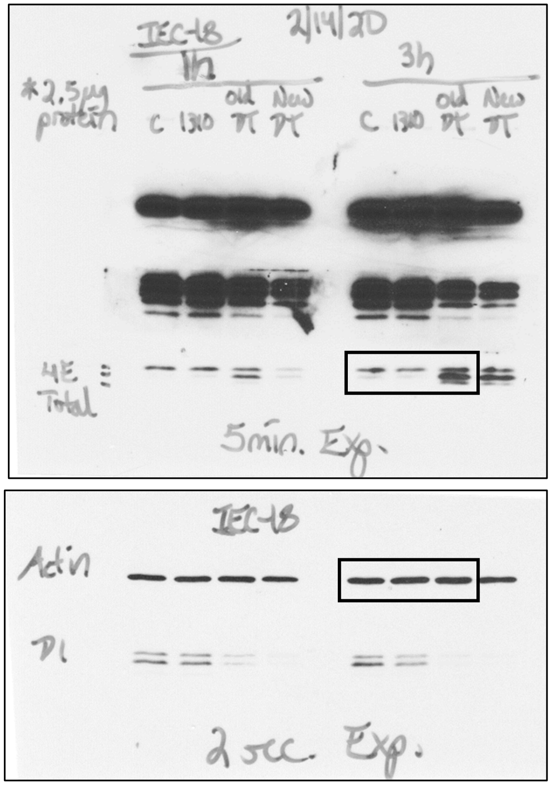

Capan-1 (PDAC)

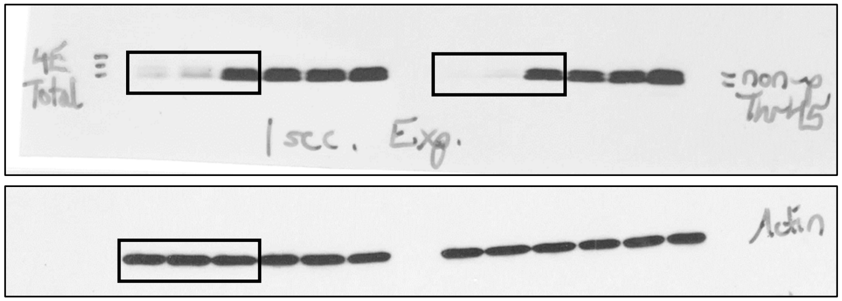

SNG-M (CRC)

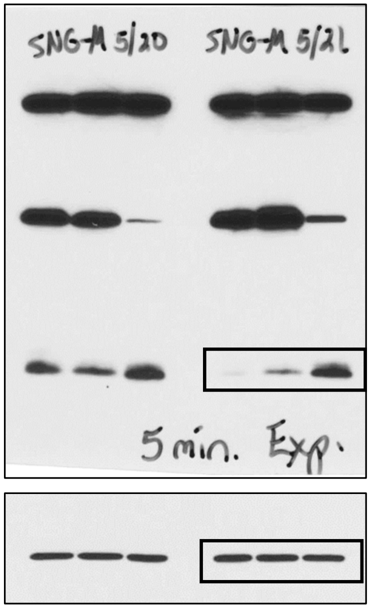

4E-BP1

Actin

FET (CRC)

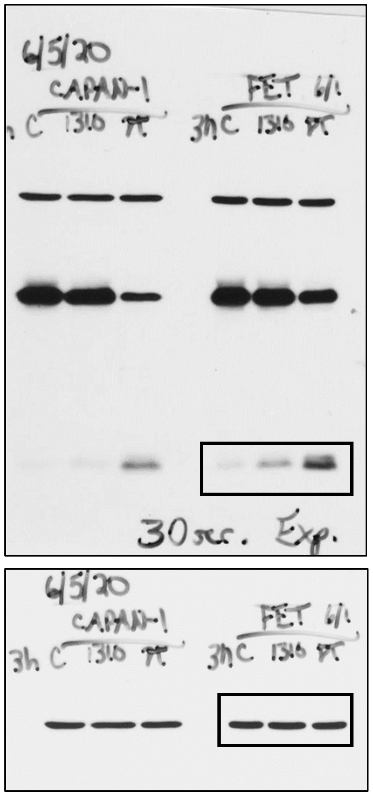

Full unedited blots for Figure 5B

5B

IEC-18 **FET**

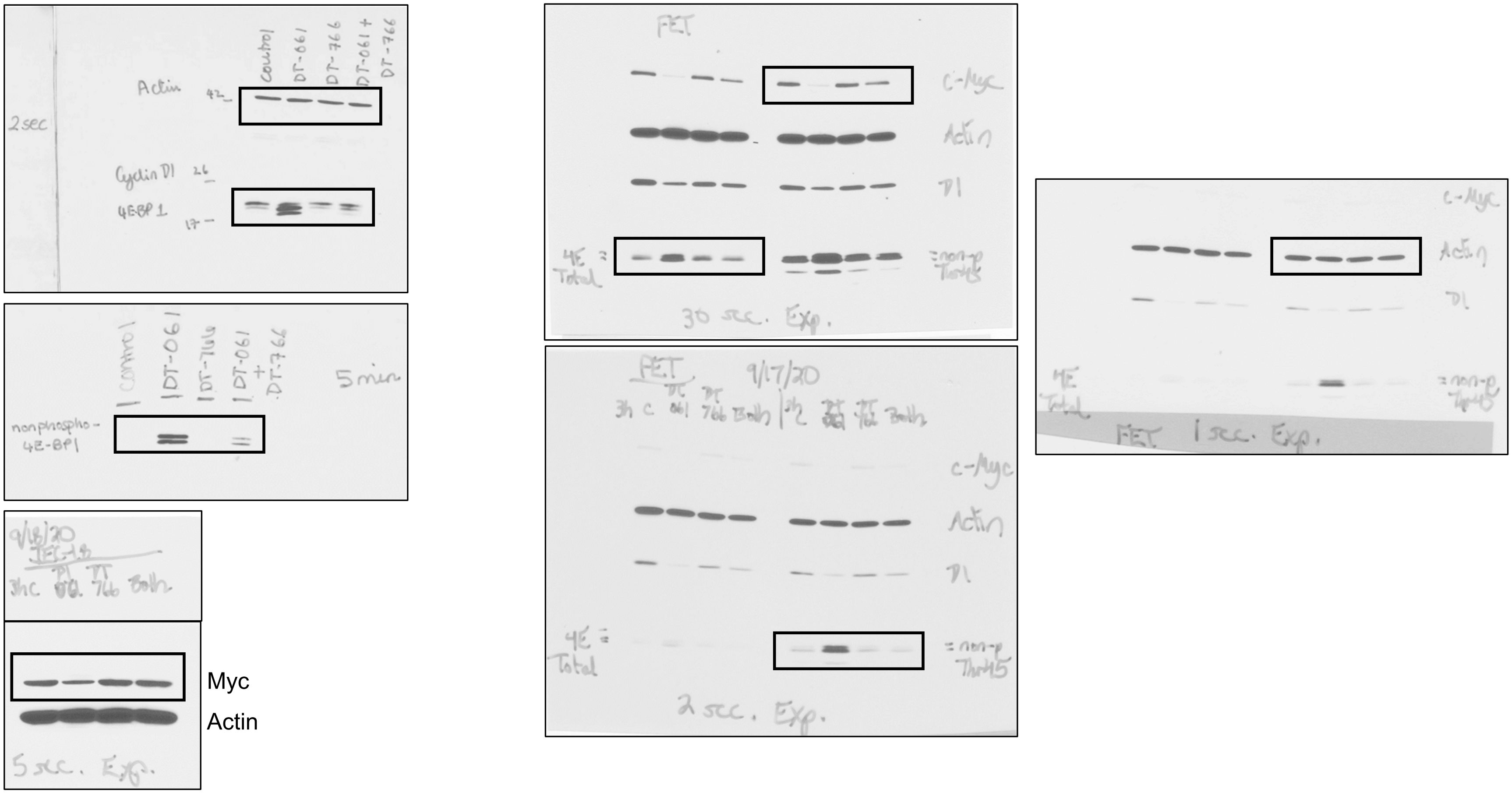

Full unedited blots for Figure 5B (continued)

5B

HCT-15

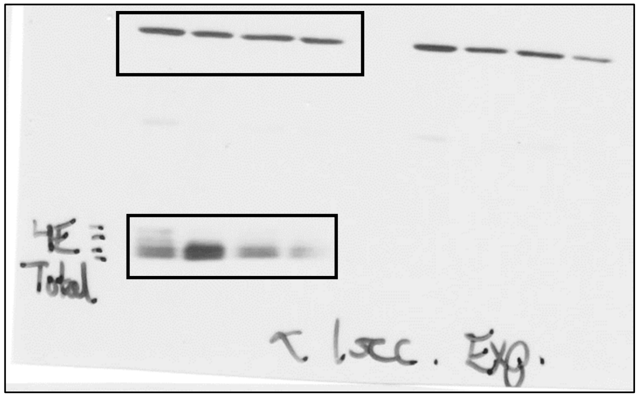

Actin

S2-013

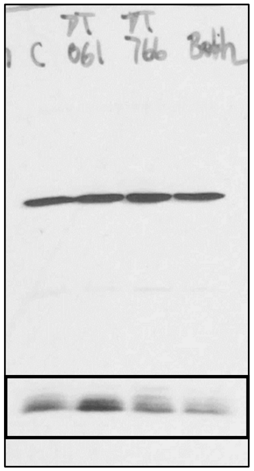

Actin

4E-BP1
Total

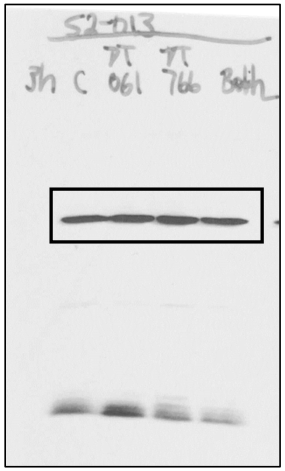

Actin

Full unedited blots for Figure 5C

5C

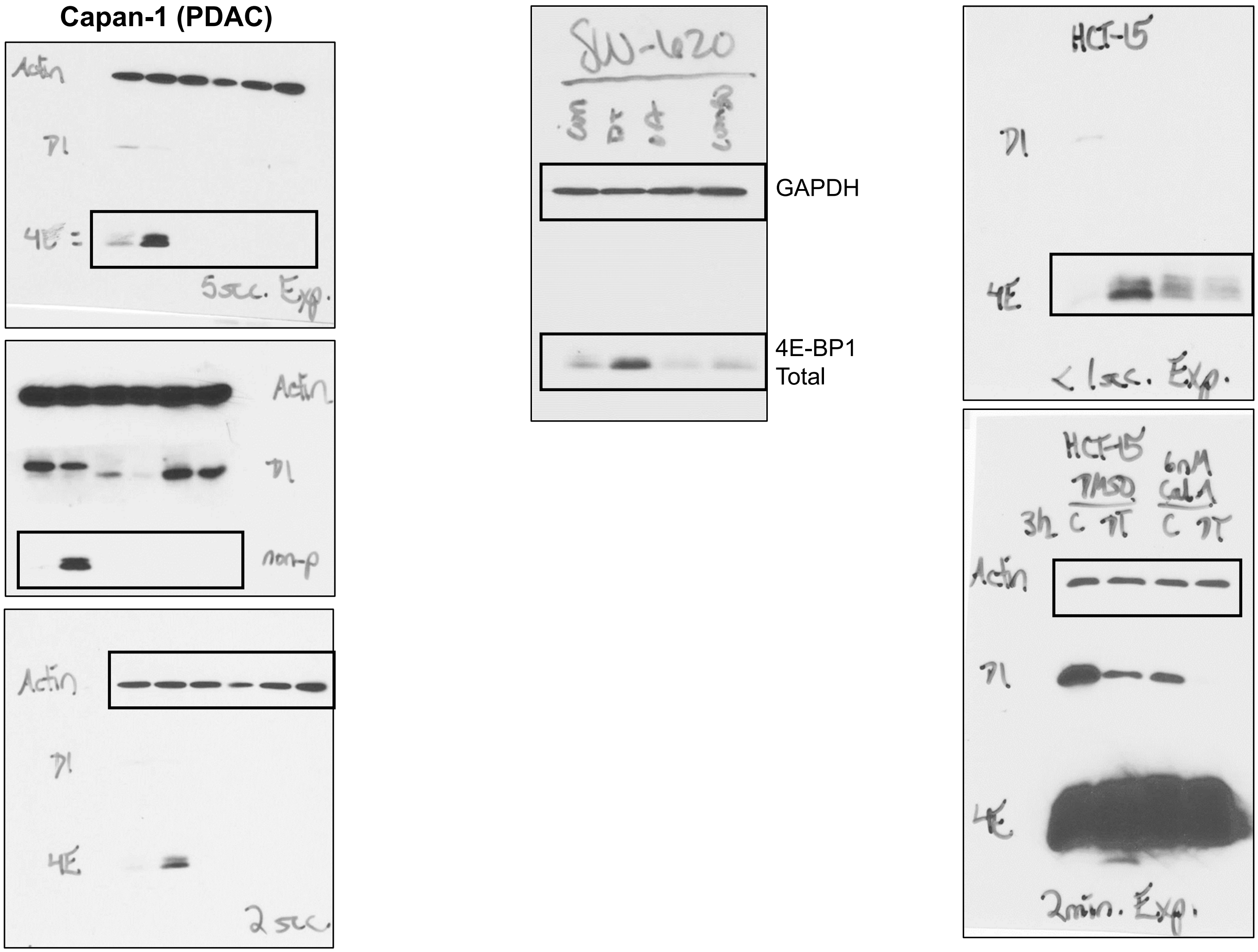

Full unedited blots for Figure 5D

5D

IEC-18 cells

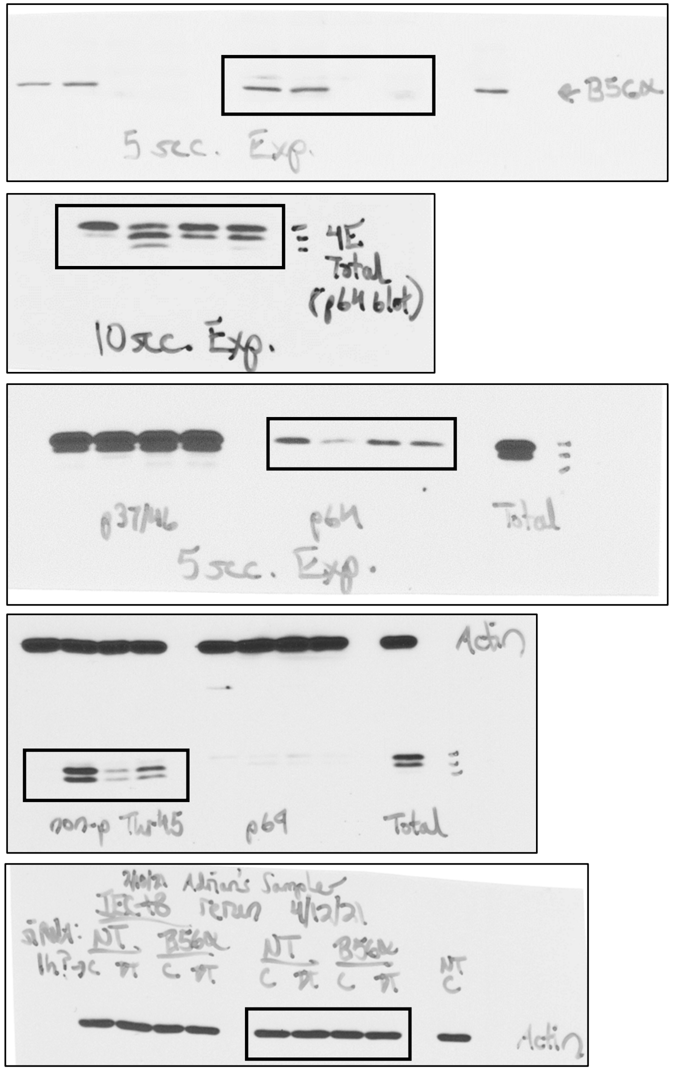

FET (CRC cells)

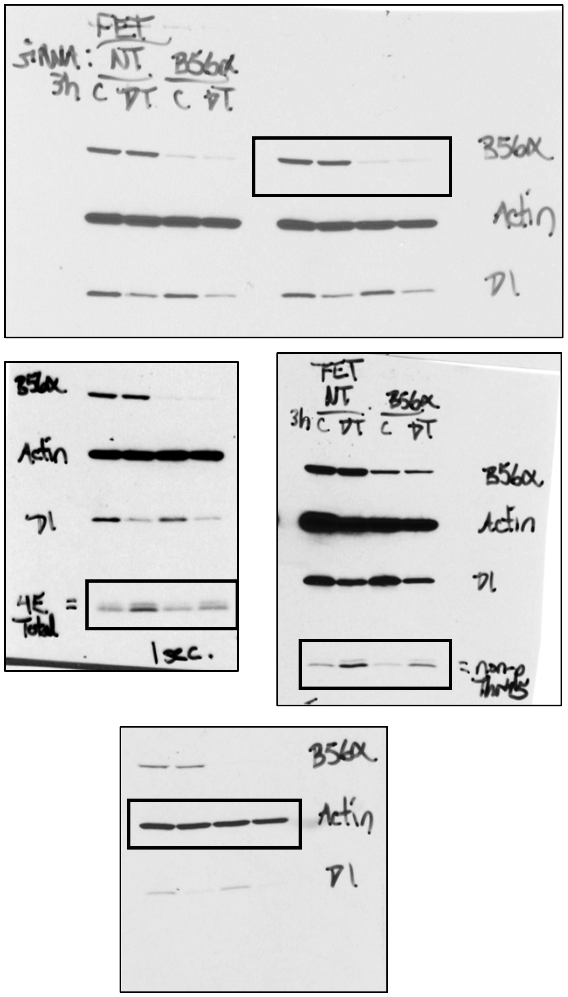

Full unedited blots for Figure 6B

HCT-15

6B

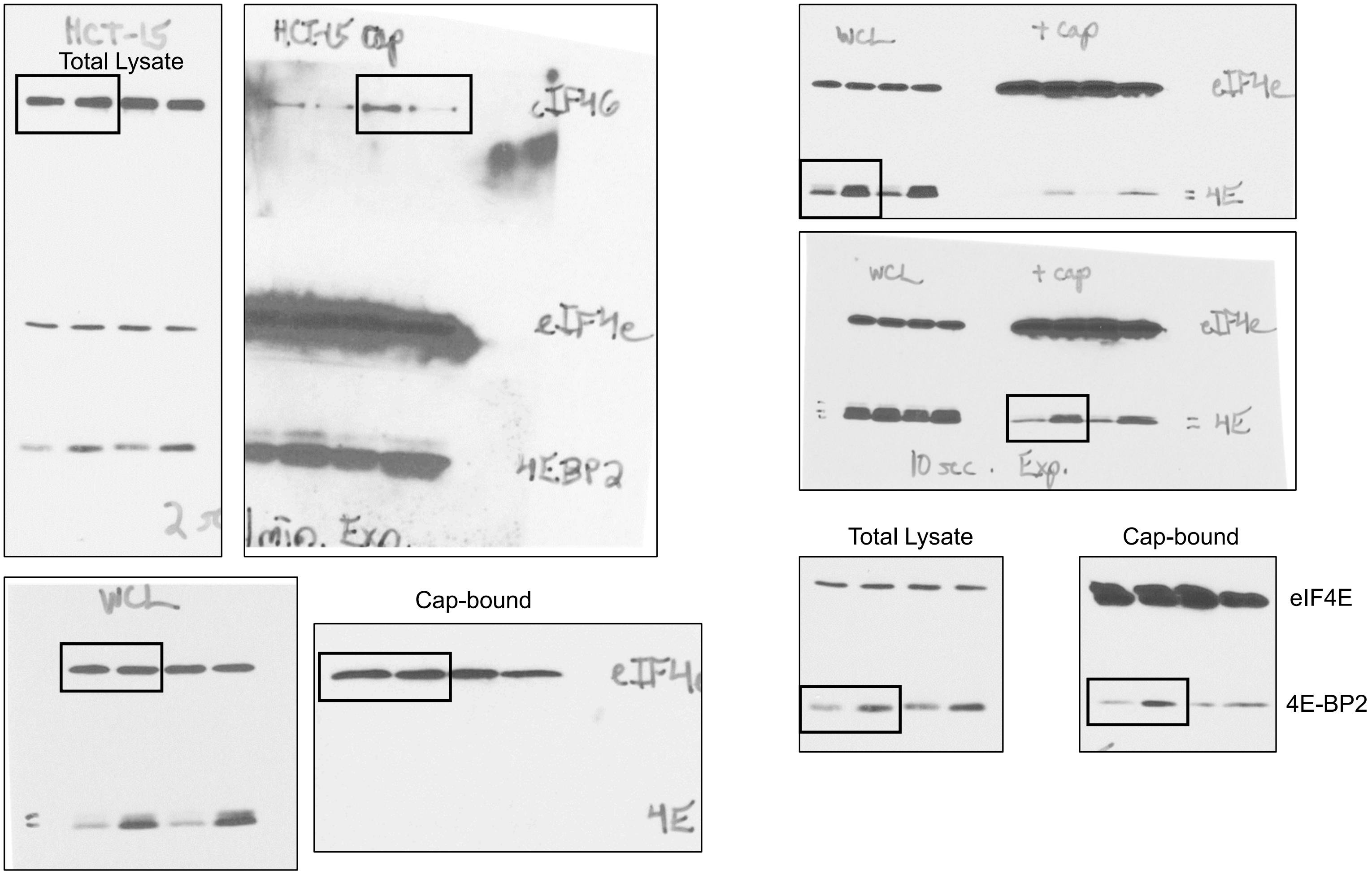

Full unedited blots for Figure 6B (continued)

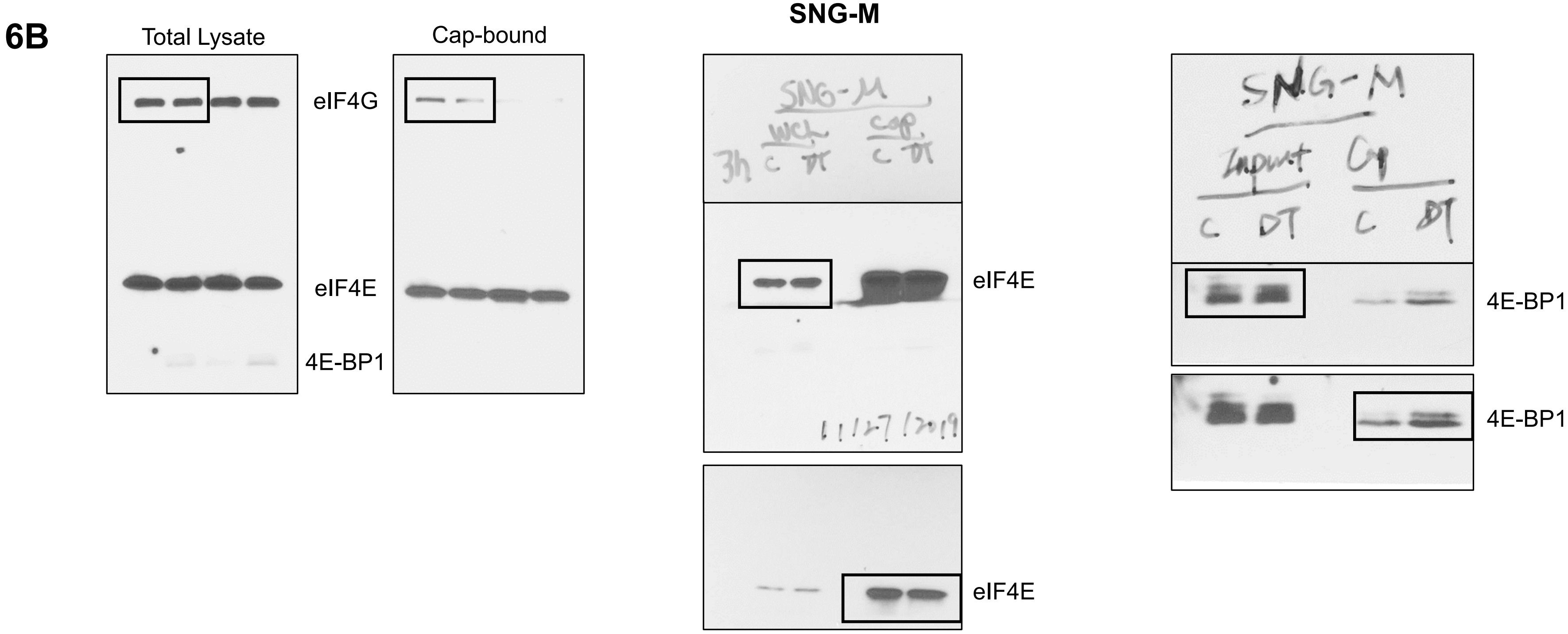

Full unedited blots for Figure 6C

6C

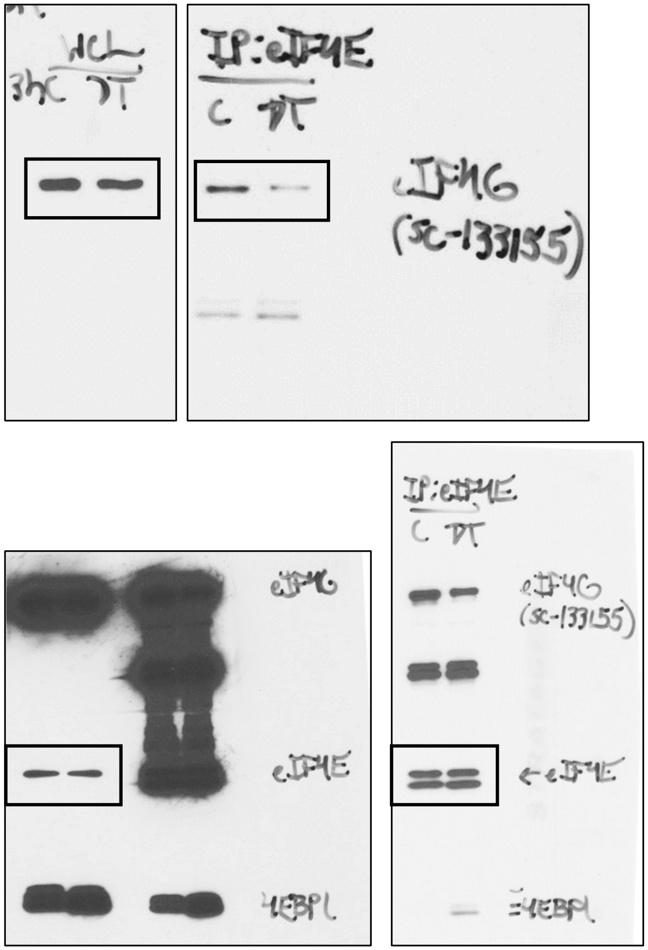

SW-620

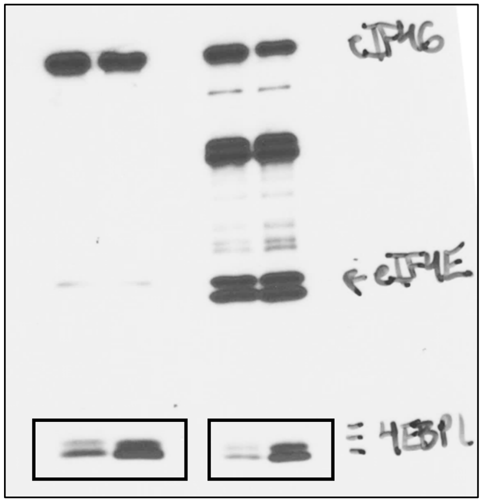

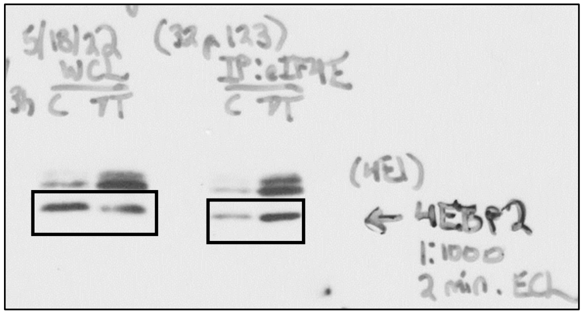

Full unedited blots for Figure 6C

Ishikawa

6C

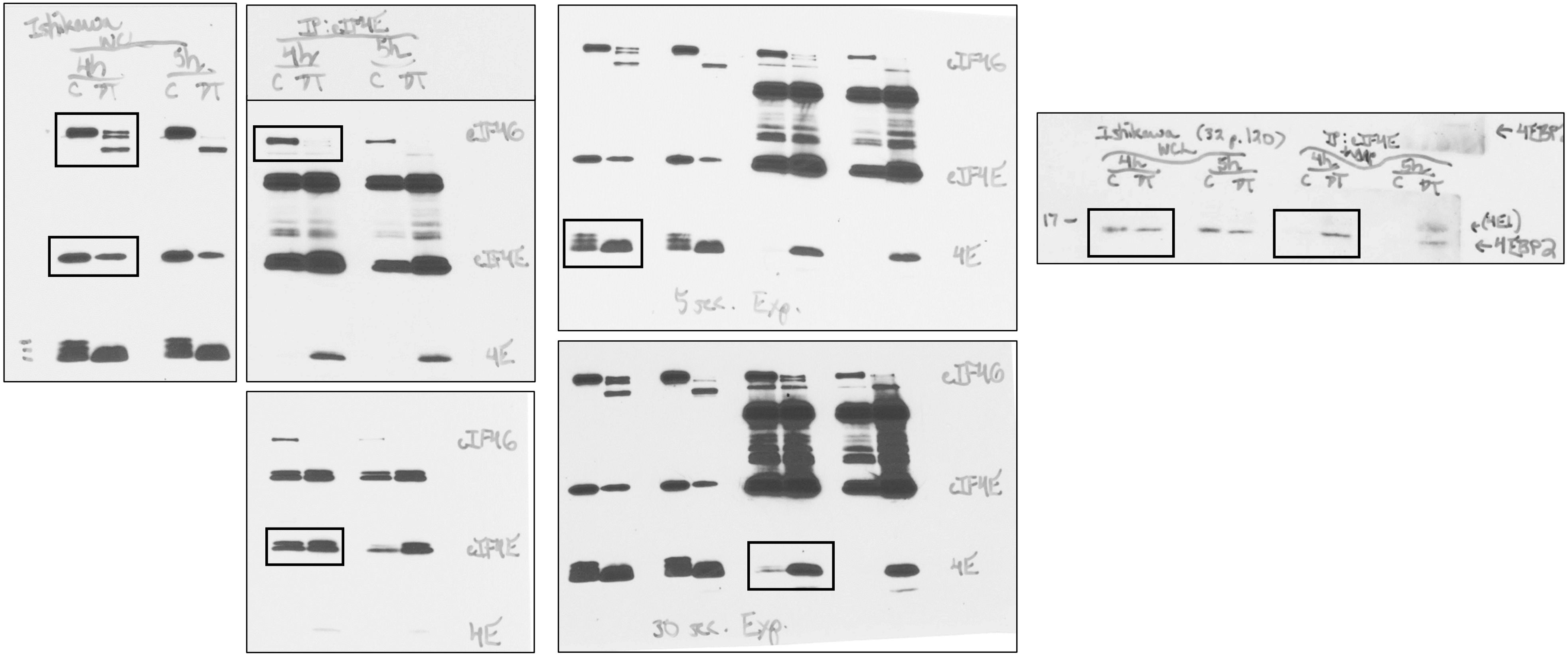

Full unedited blots for Figure 6E

6E

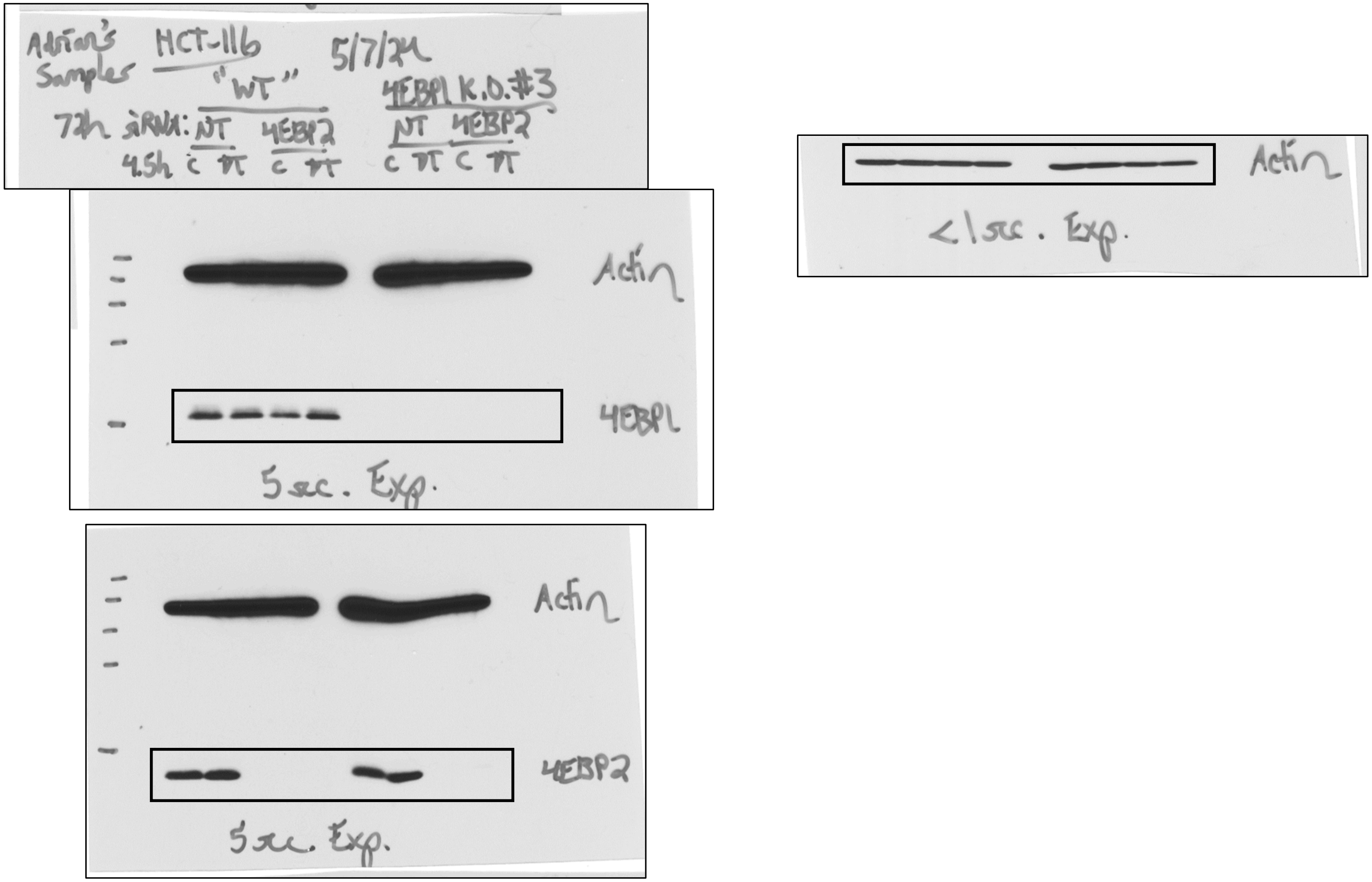

Full unedited blots for Figure 7A

7A

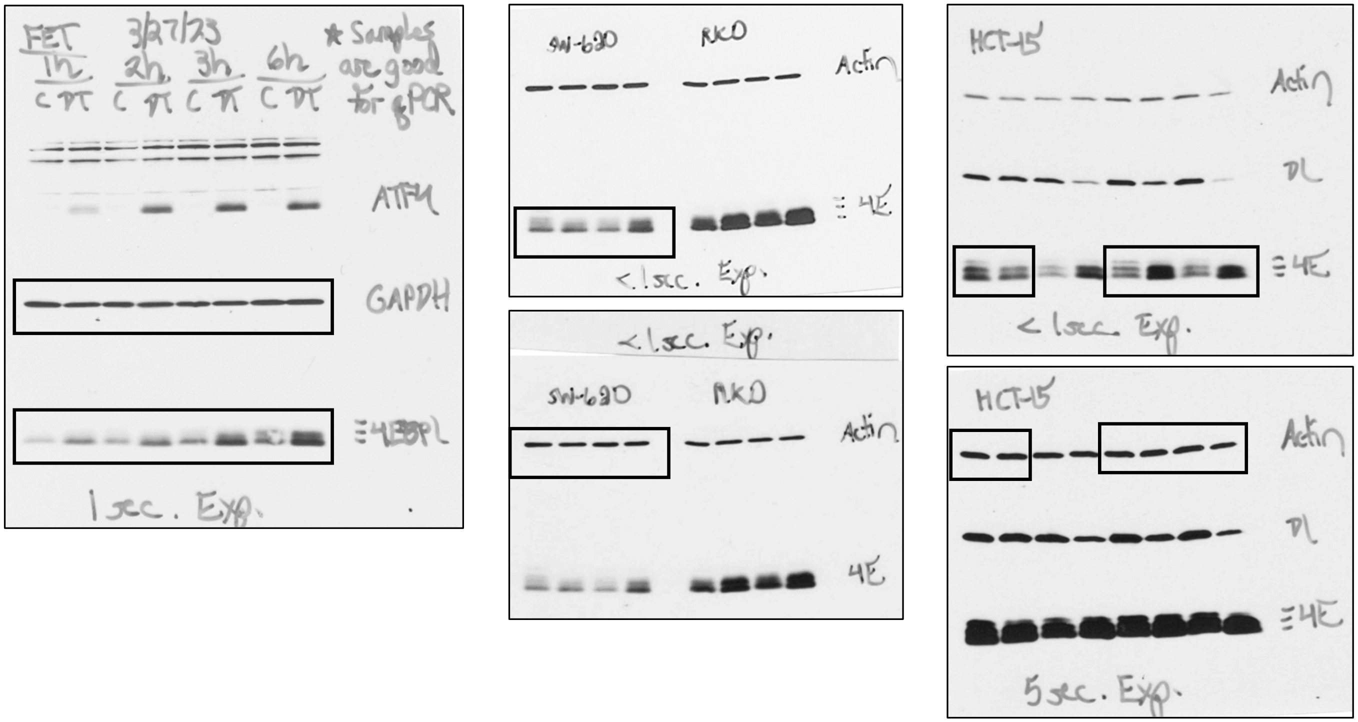

Full unedited blots for Figure 7A (continued)

7A

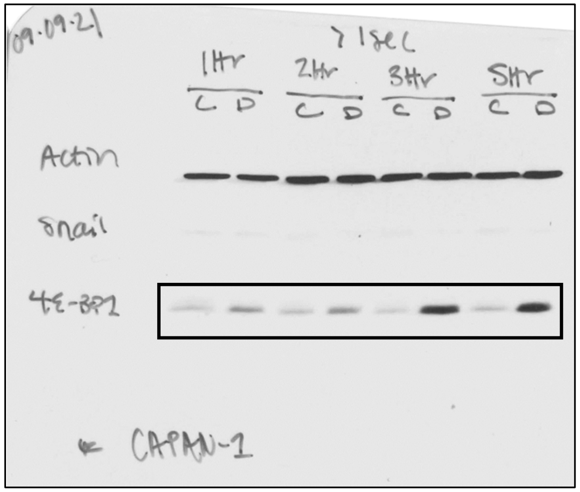

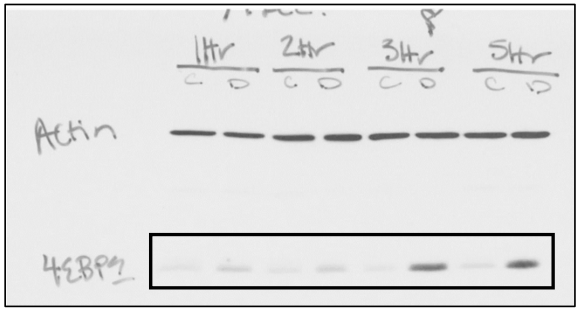

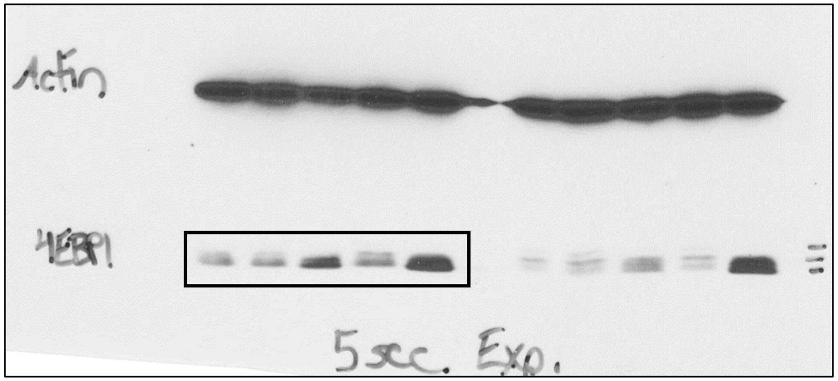

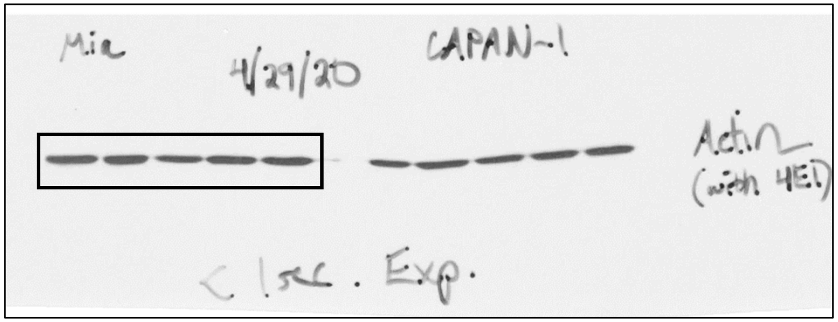

Full unedited blots for Figure 7B

7B

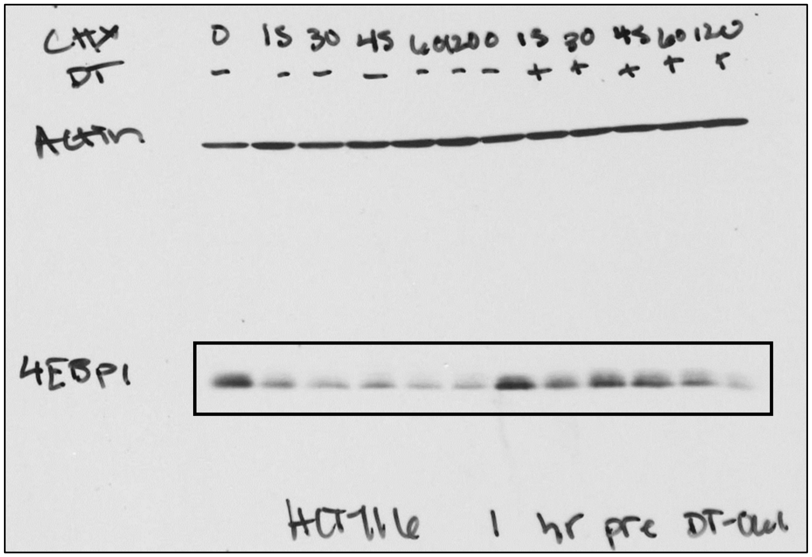

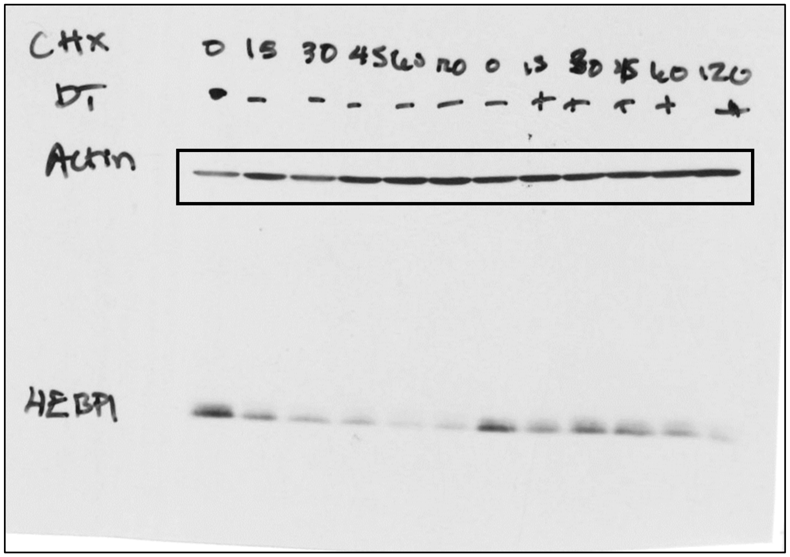

Full unedited blots for Figure 7D

7D

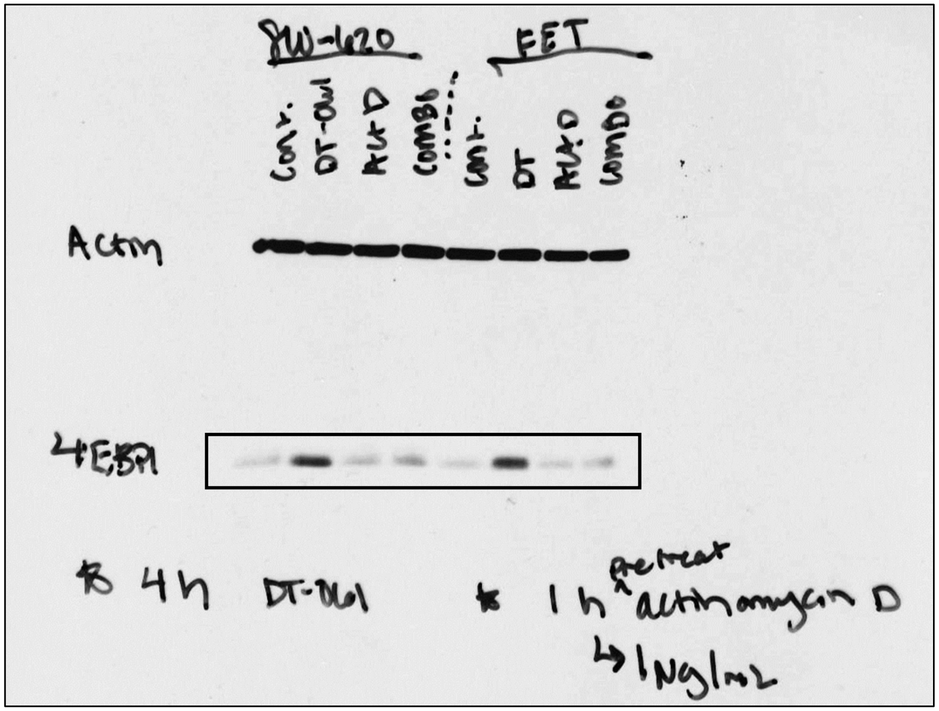

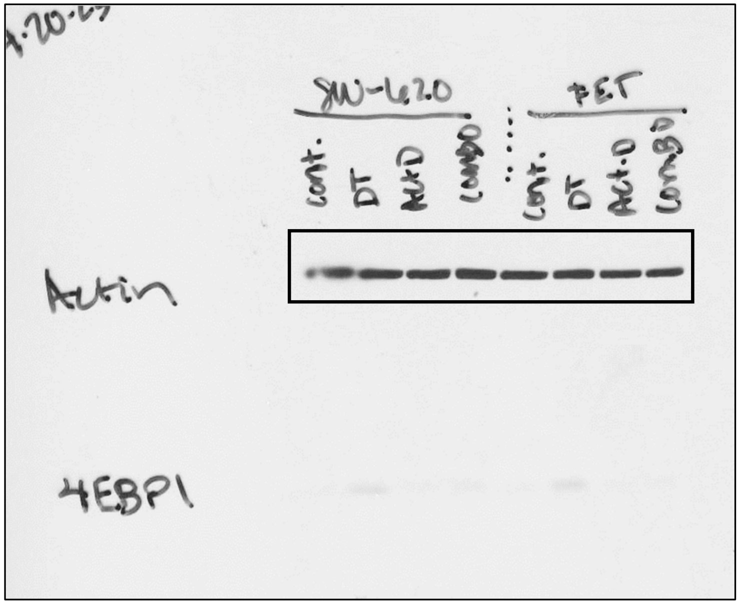

Full unedited blots for Figure 8A-B

8A

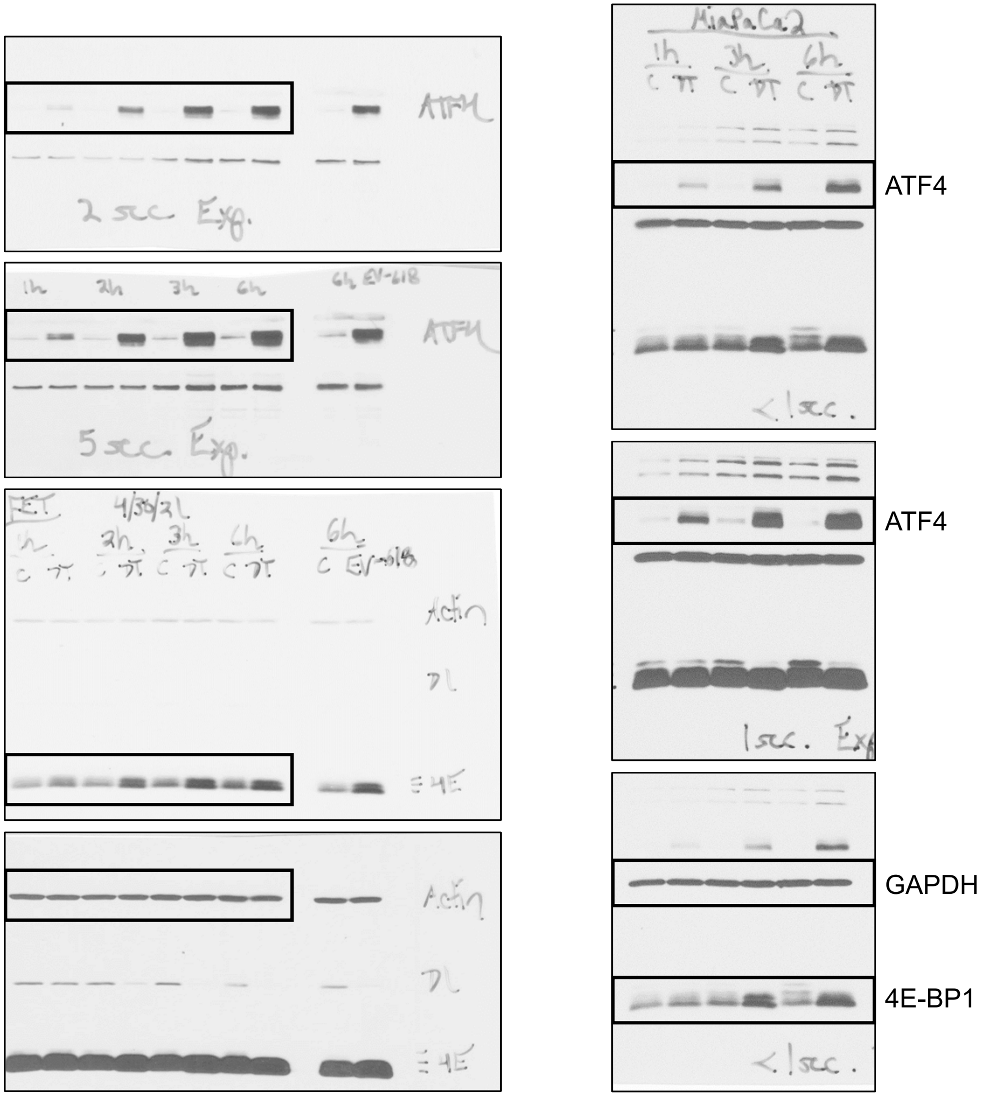

8B

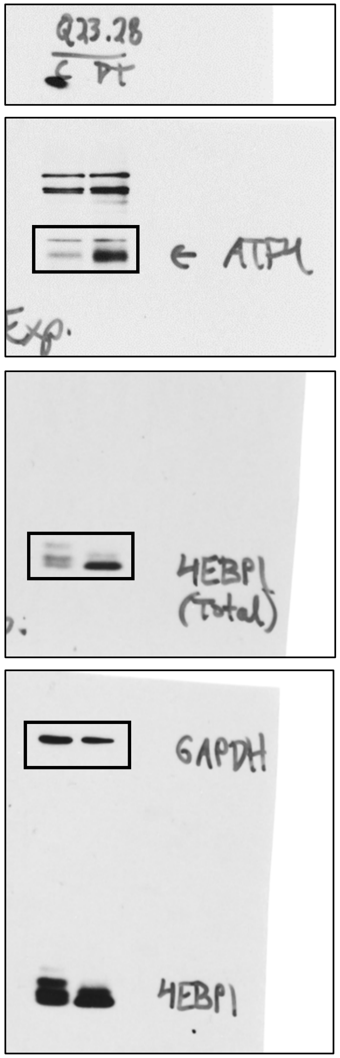

Full unedited blots for Figure 8C-D

8C

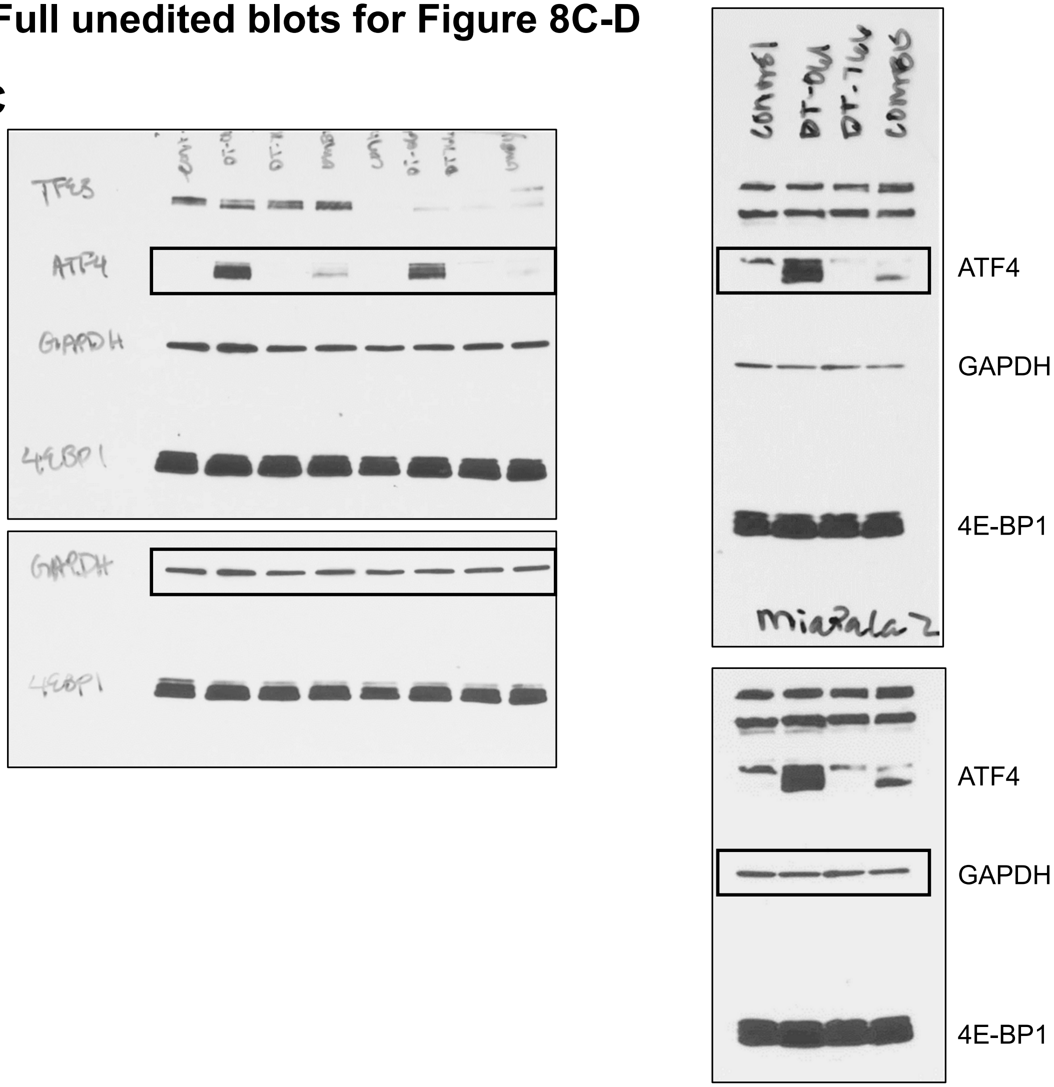

8D

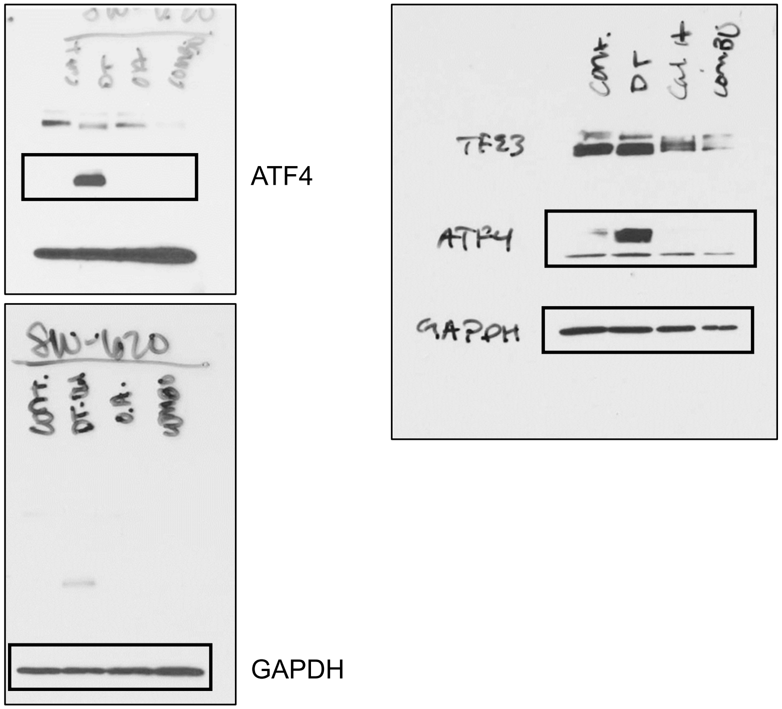

Full unedited blots for Figure 8E-F

8E

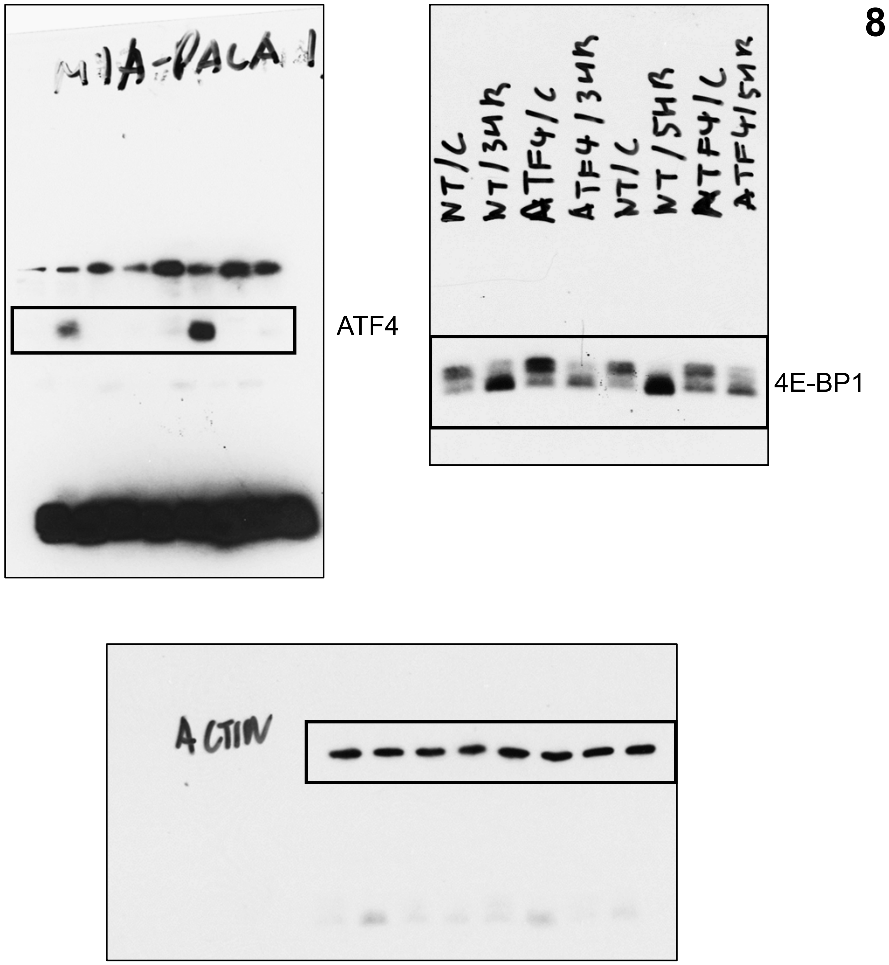

8F

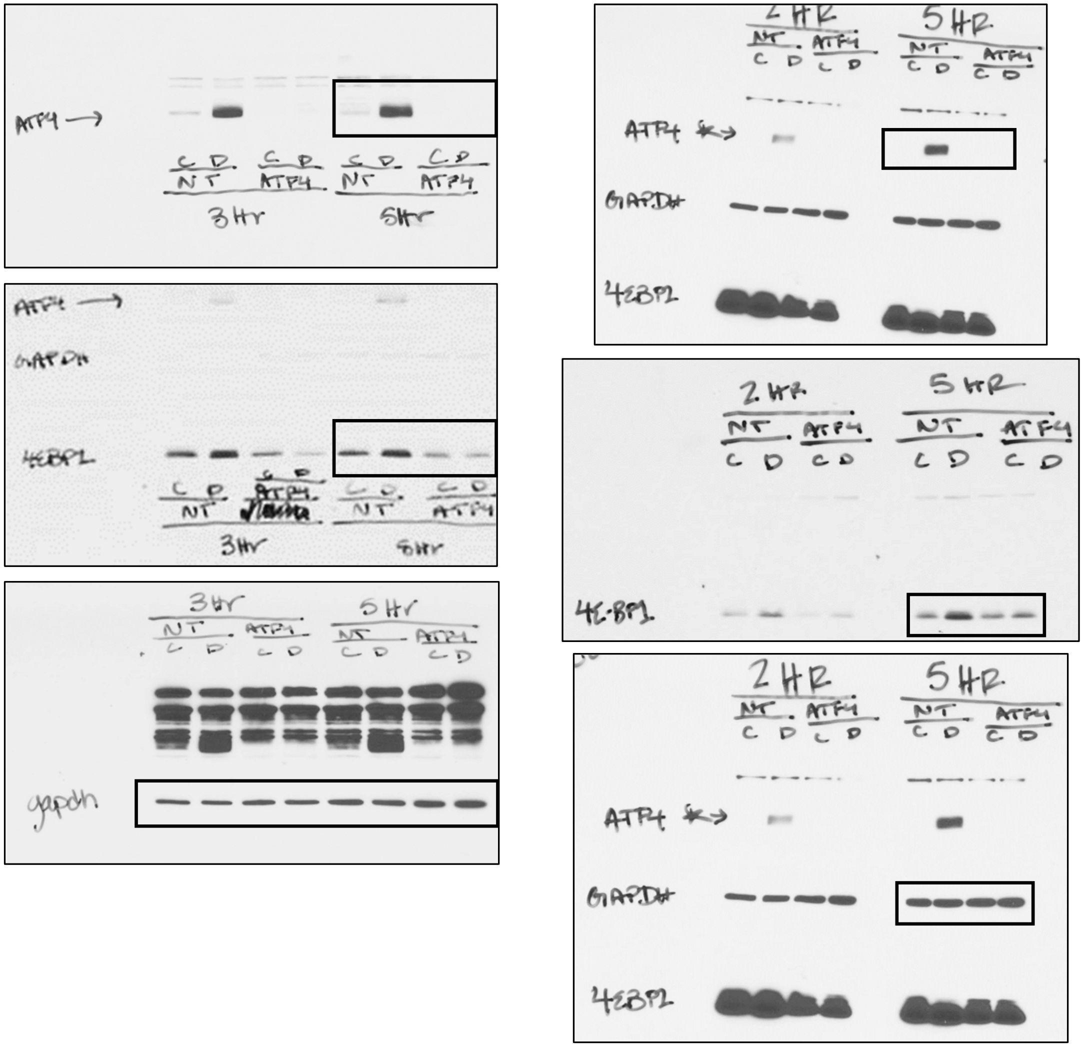

Full unedited blots for Figure 8G-I

8G

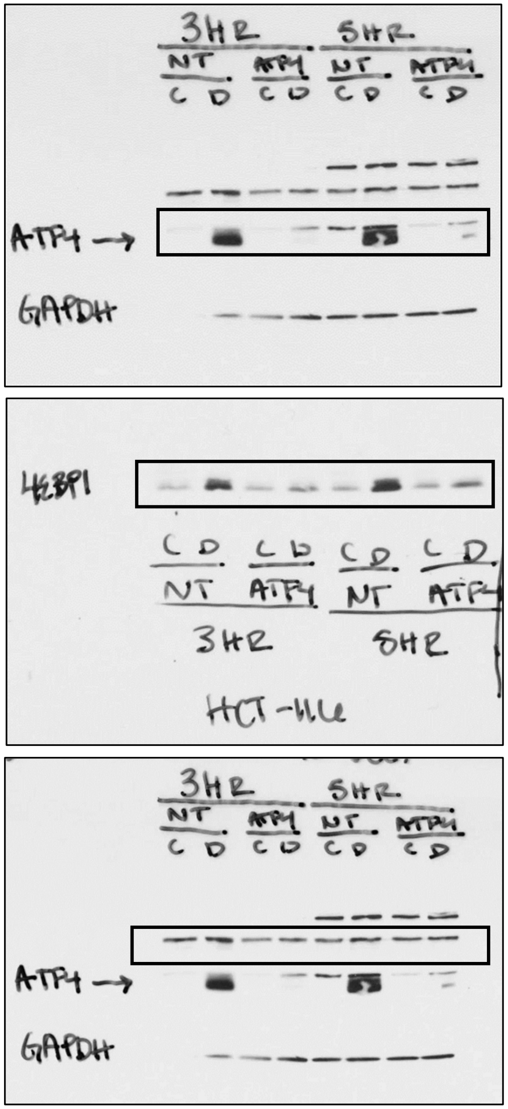

8I

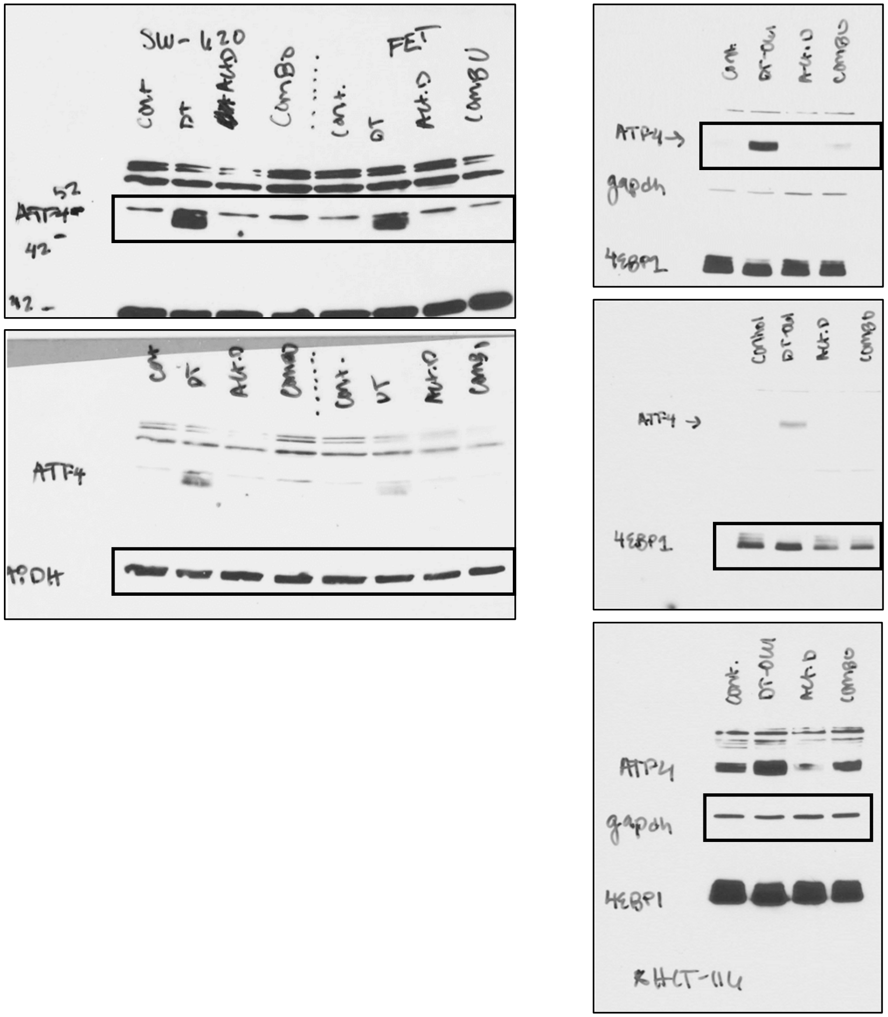

Full unedited blots for Figure 9A

9A

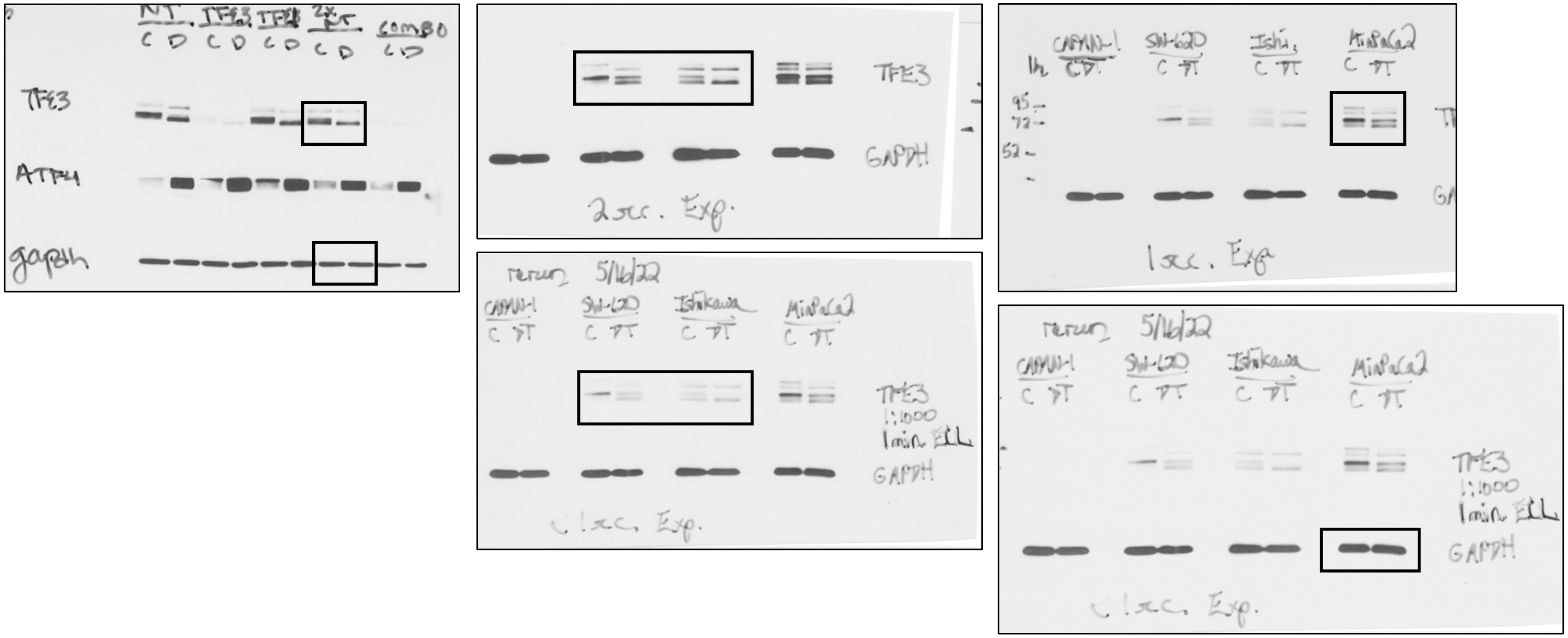

Full unedited blots for Figure 9B-C

9B

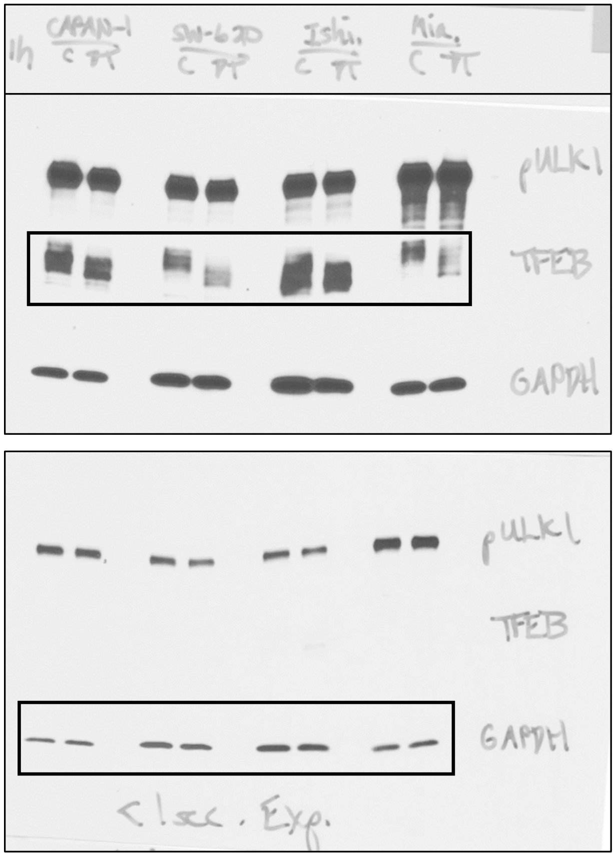

9C

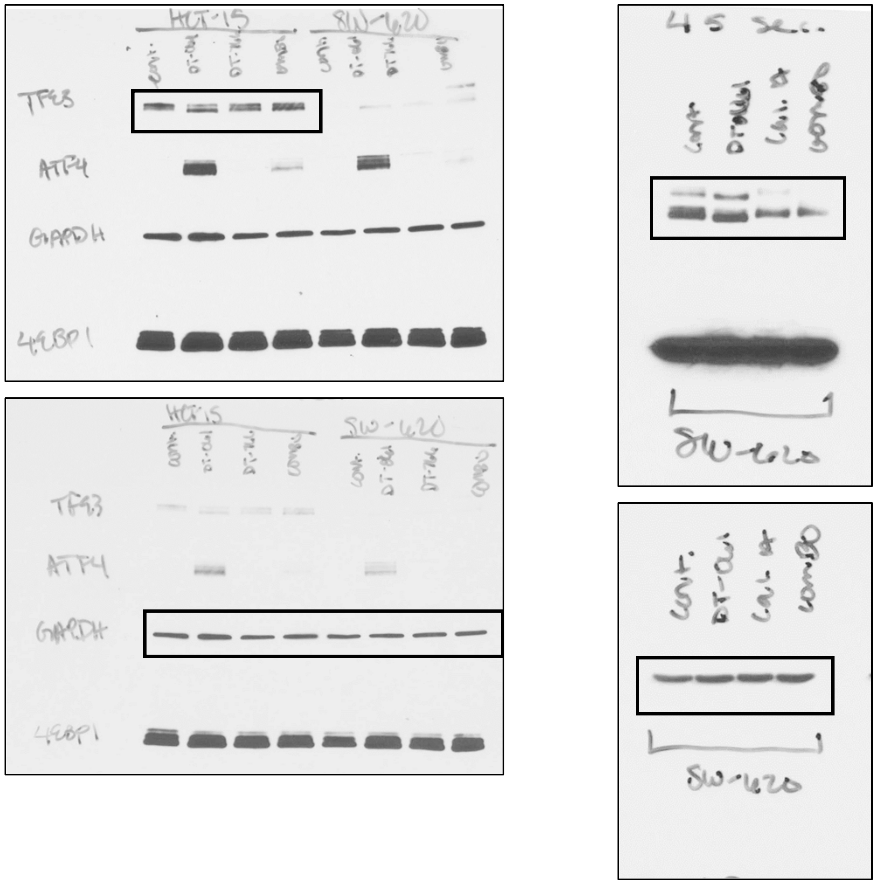

TFE3

GAPDH

Full unedited blots for Figure 9D,G

9D

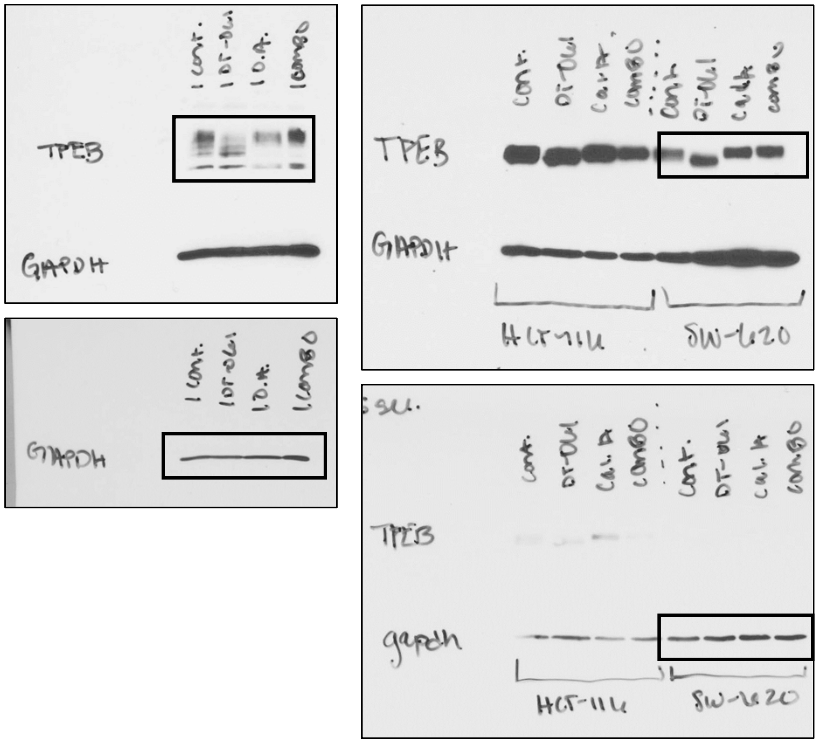

9G

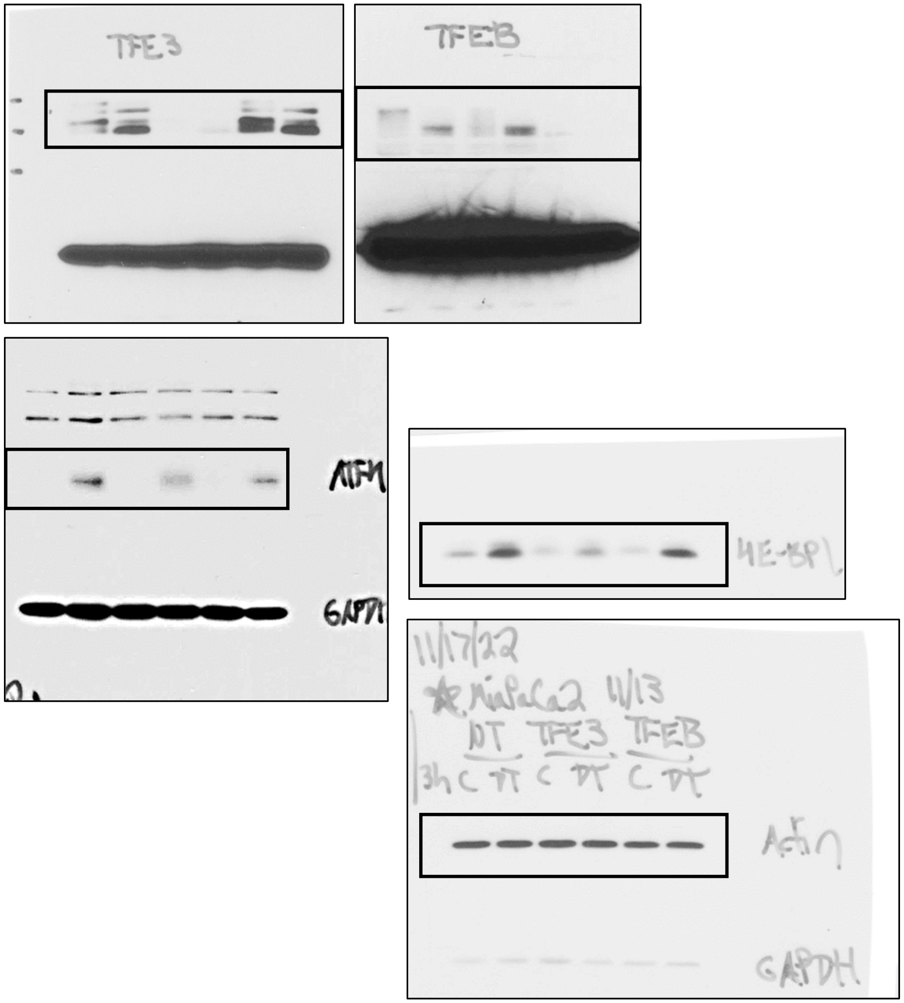

Full unedited blots for Figure 9H

9H

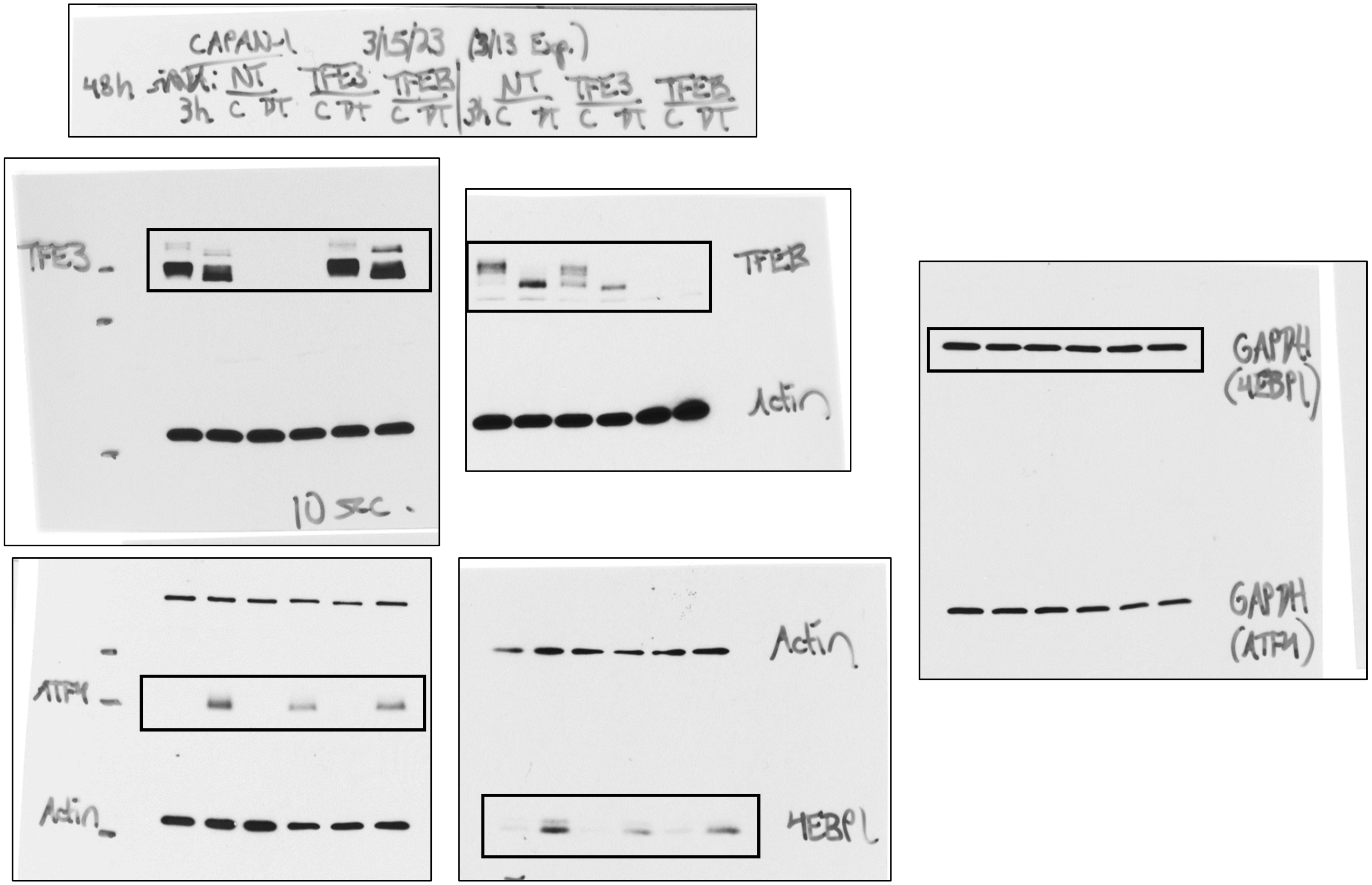

Full unedited blots for Figure 10

10B

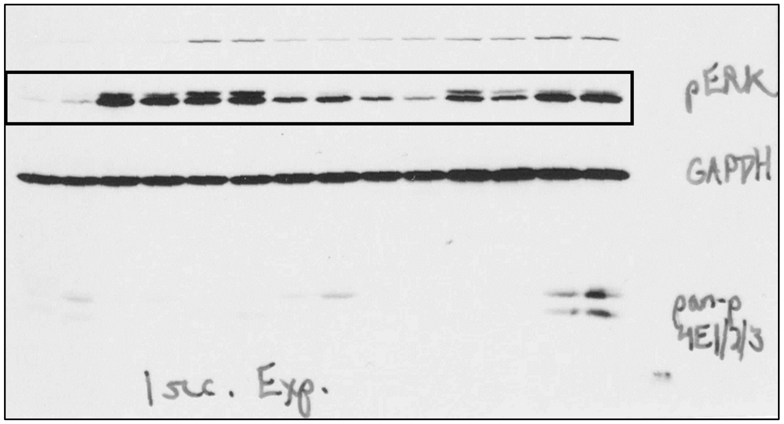

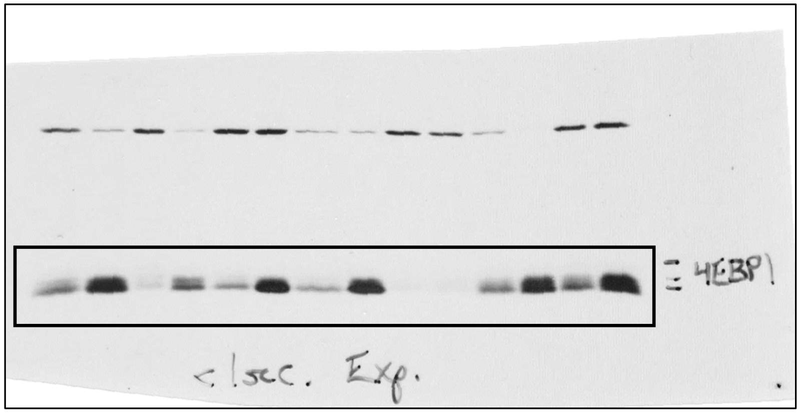

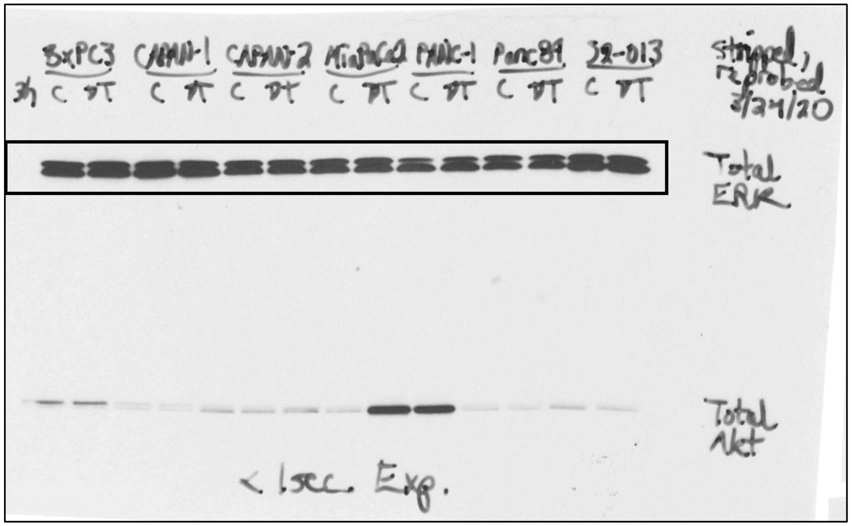

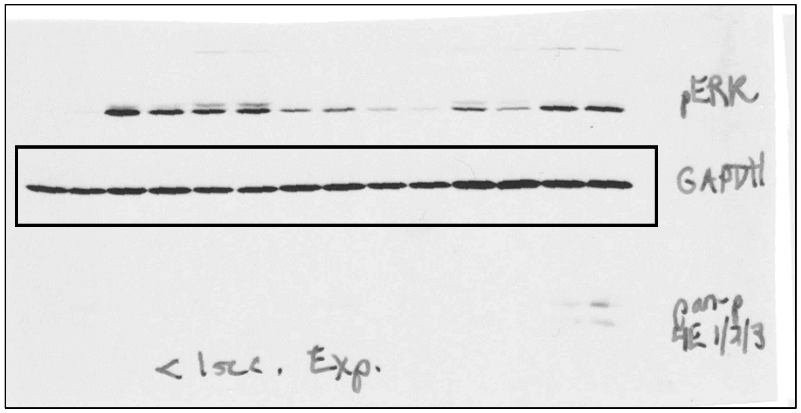

Full unedited blots for Figure 10B

10B

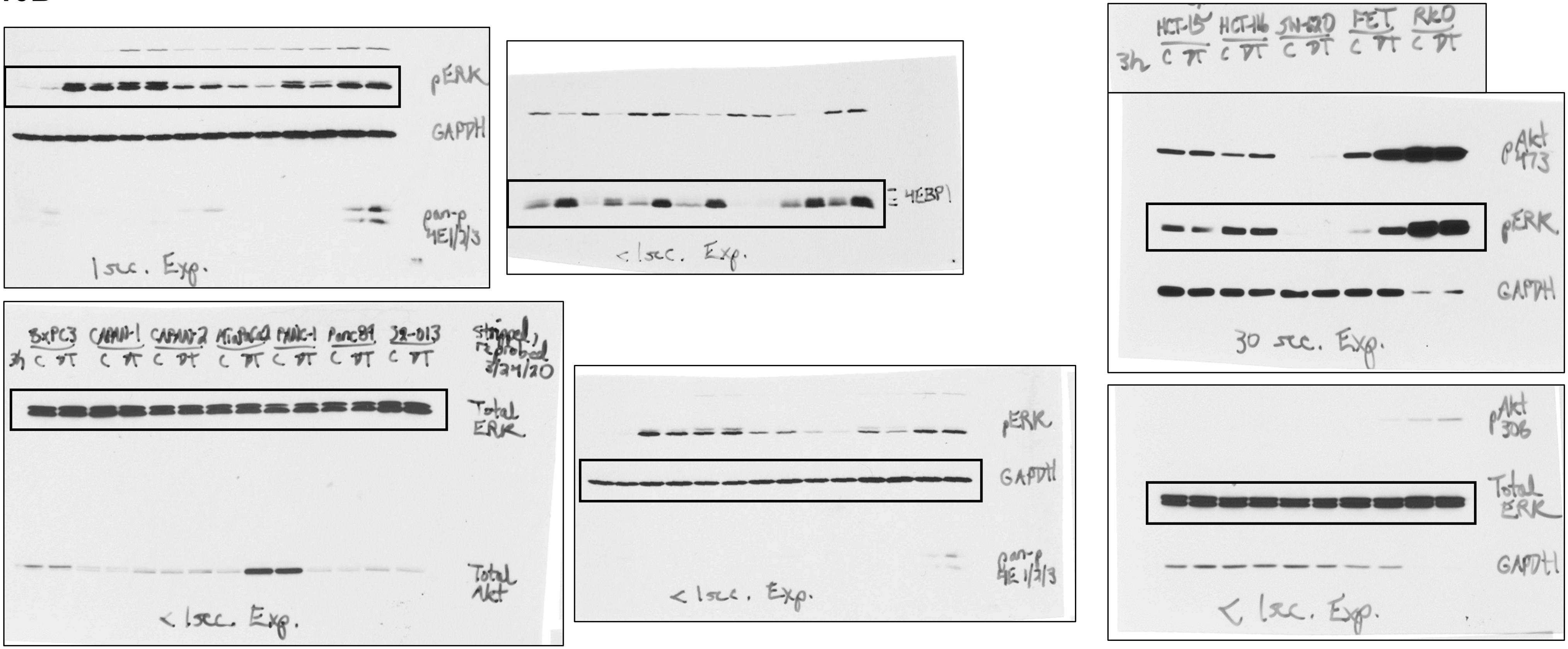

Full unedited blots for Figure 10C

10C

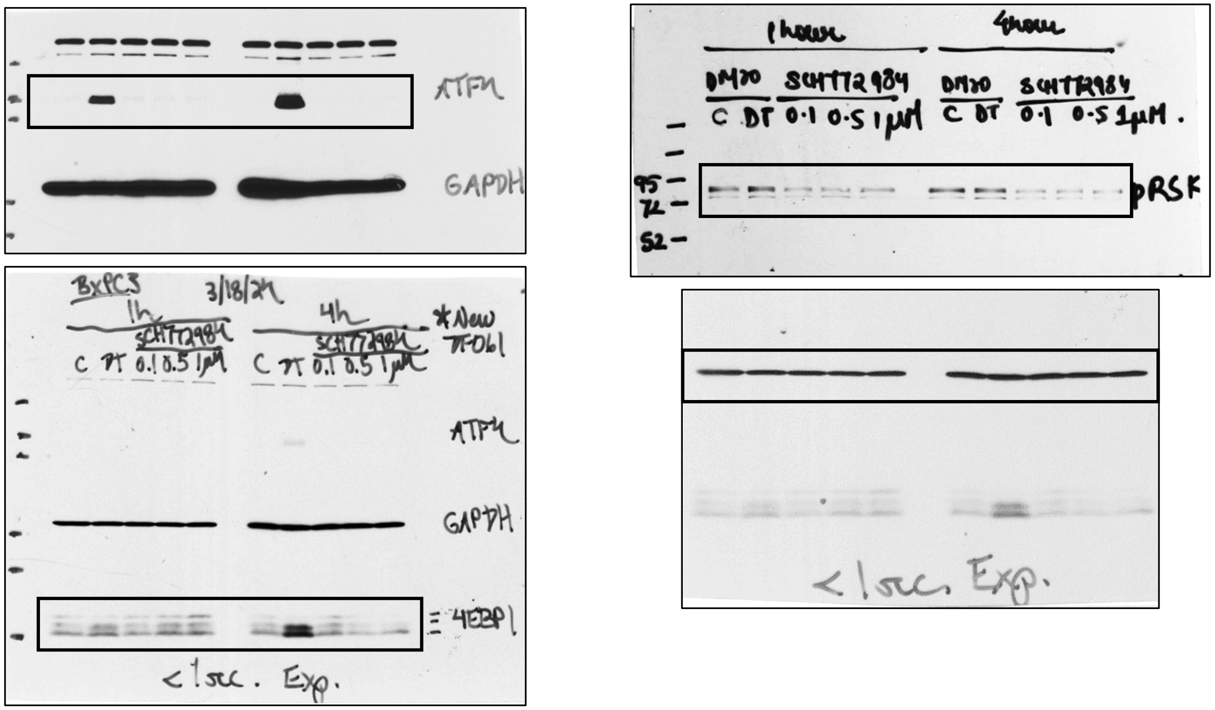

Full unedited blots for Figure 10C (continued)

10C

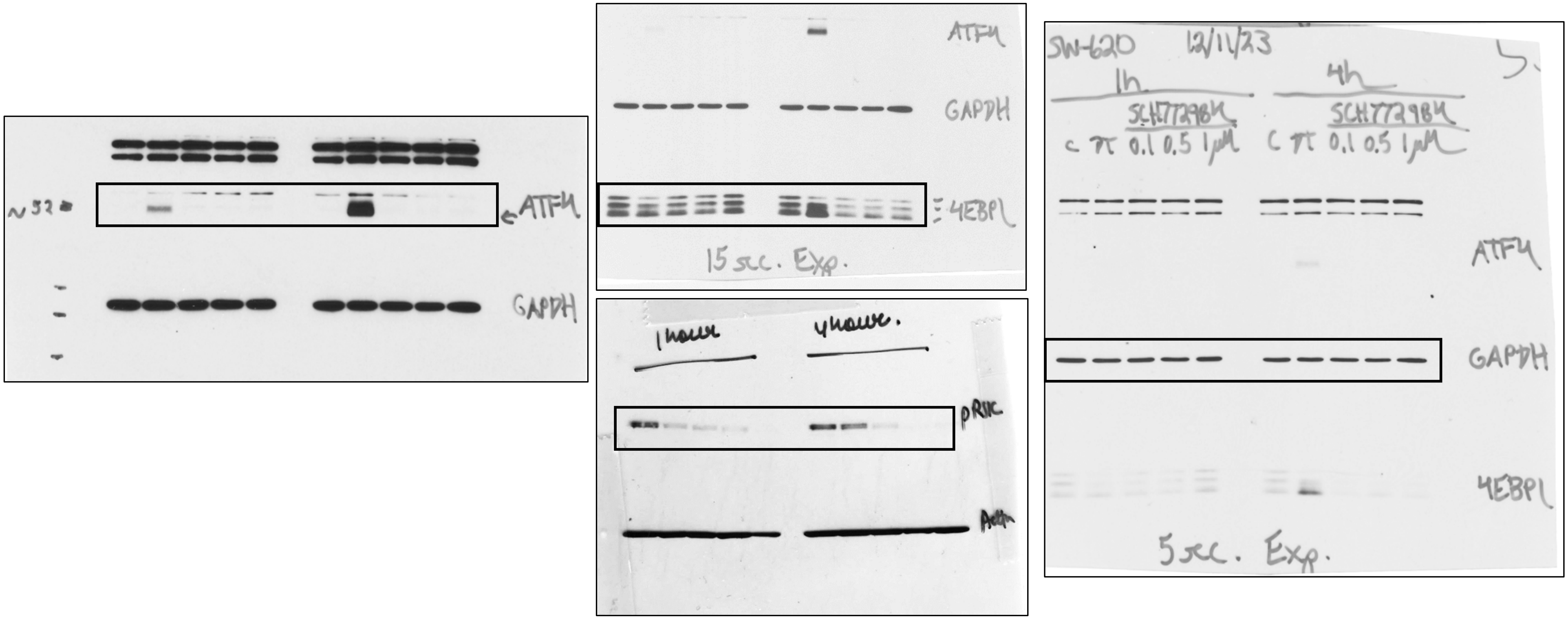

Full unedited blots for Figure 10D

10D

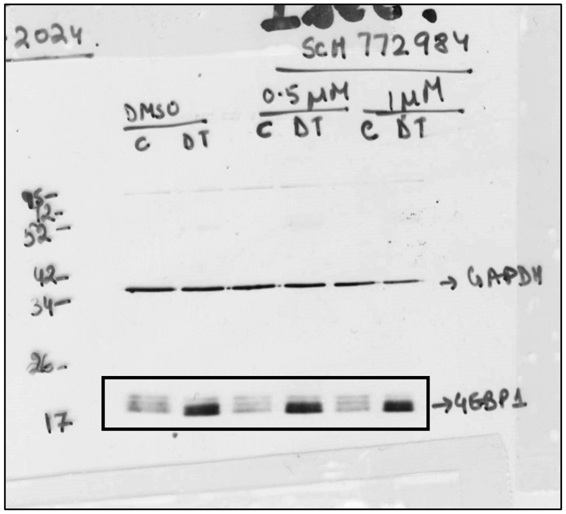

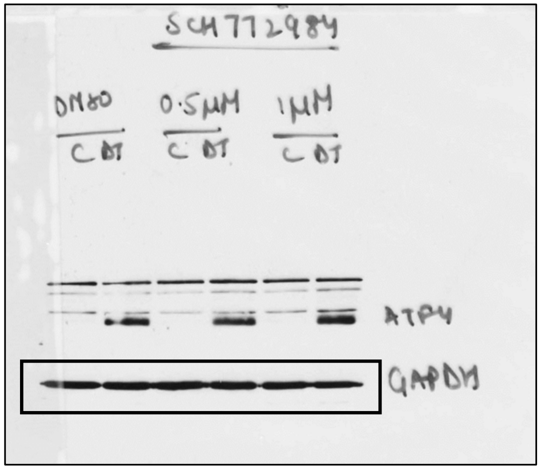

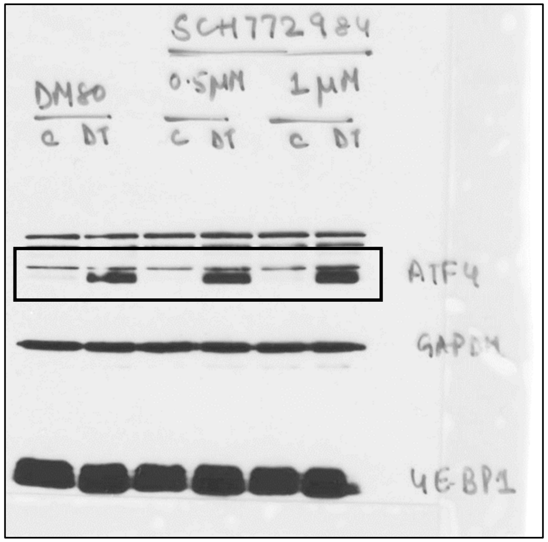

Full unedited blots for Figure 11A

11A

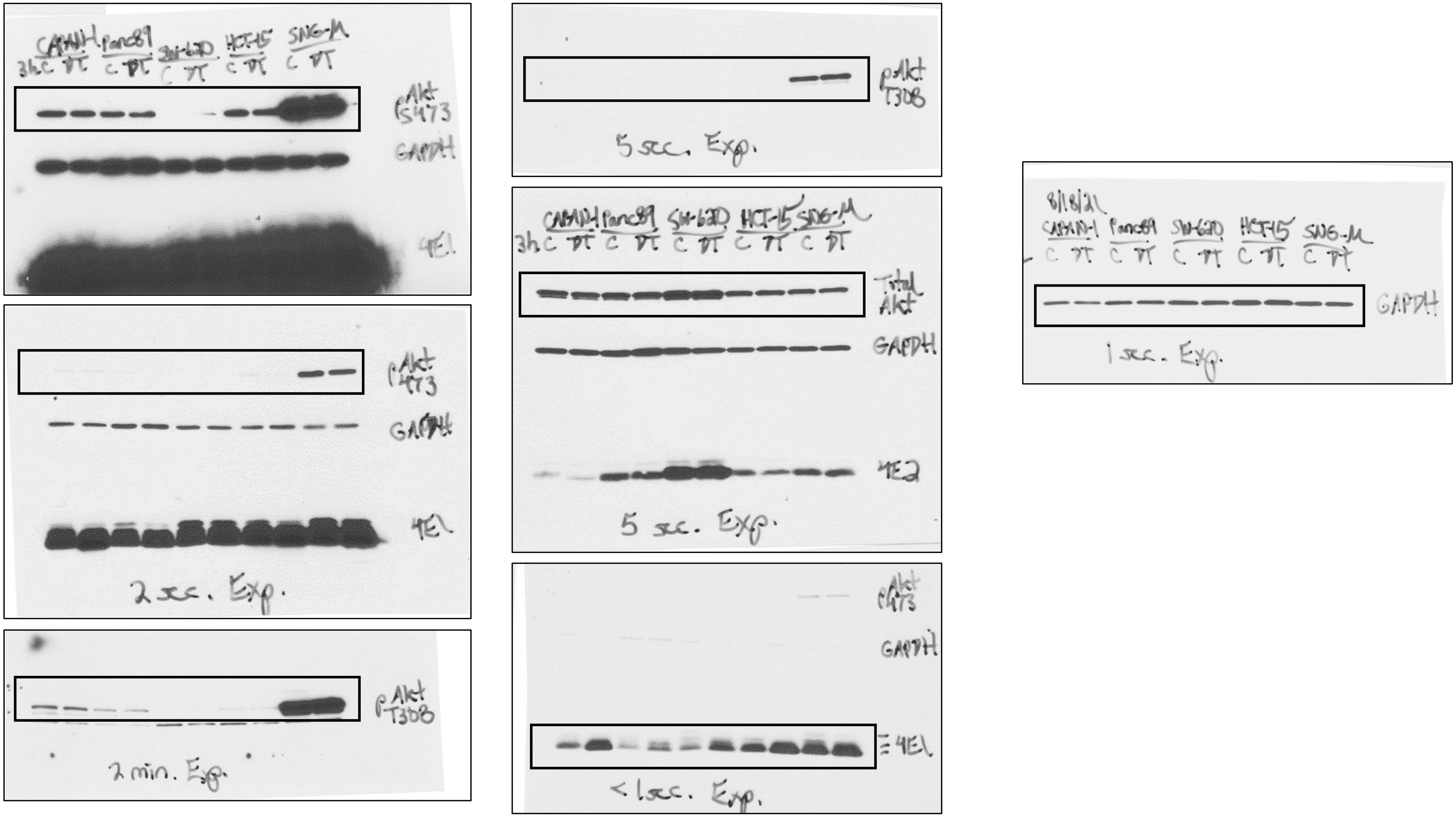

Full unedited blots for Figure 11B

11B

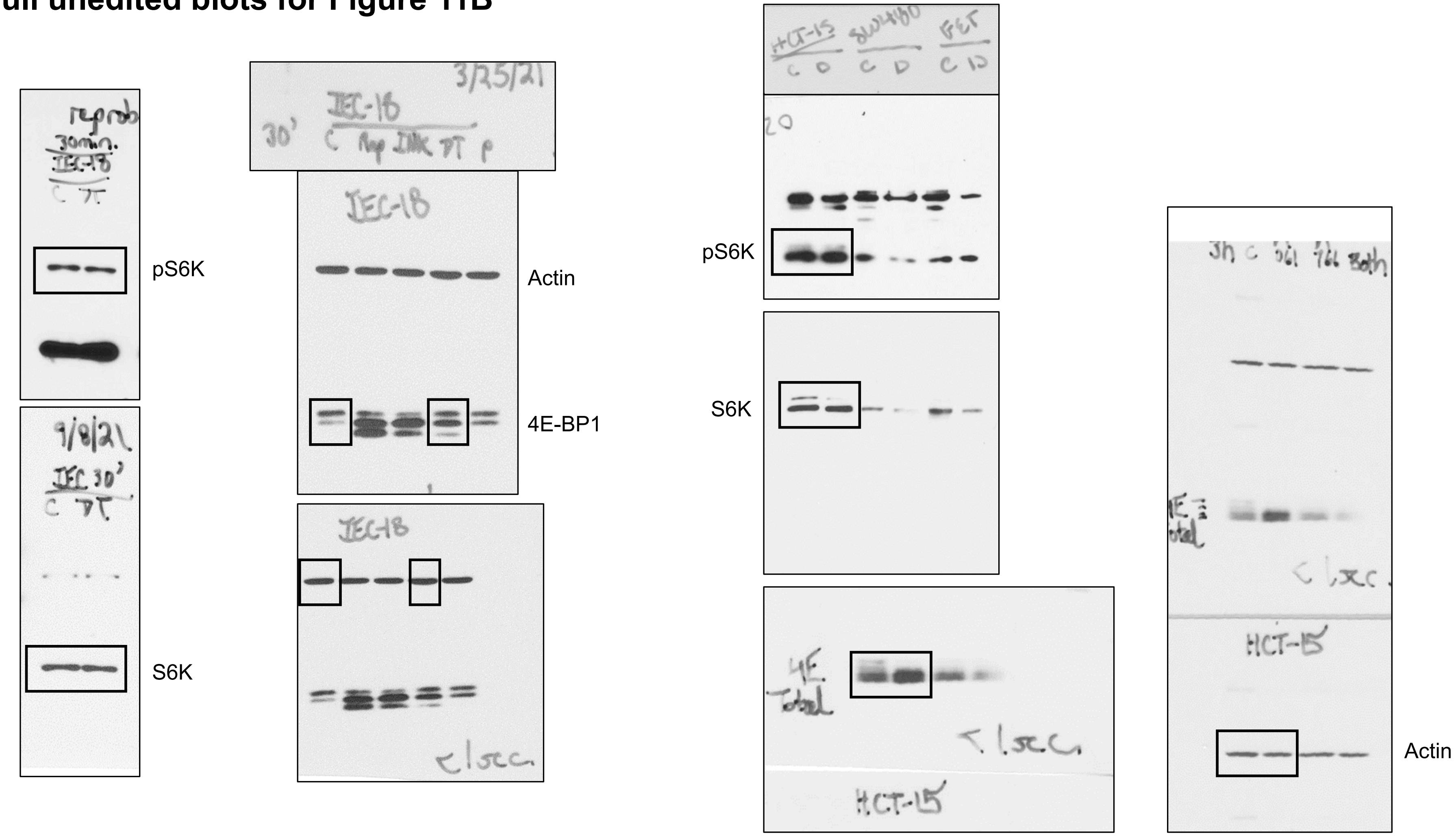

Full unedited blots for Figure 11C

11C

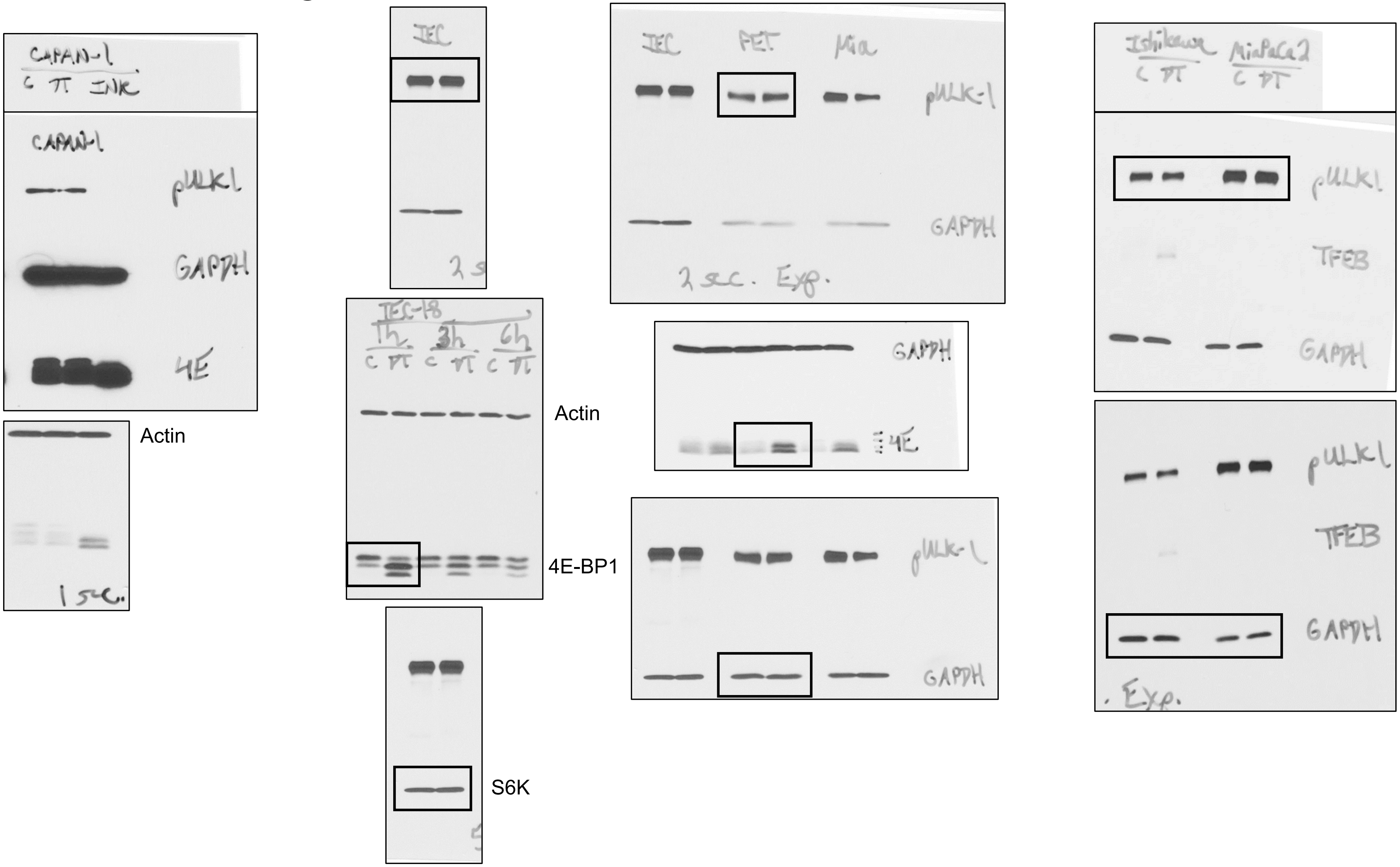

Full unedited blots for Figure 11D

11D

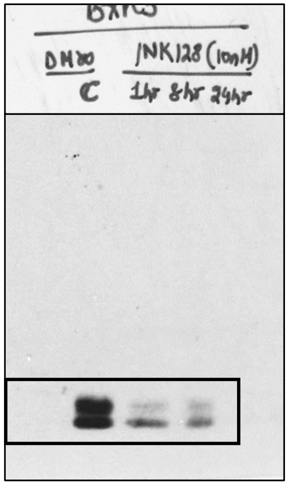

4E-BP1

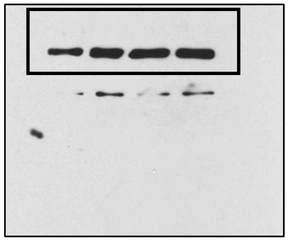

Actin

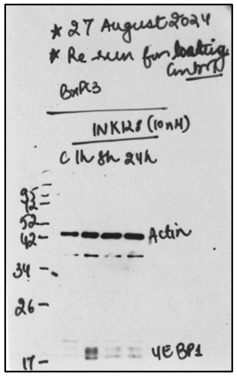

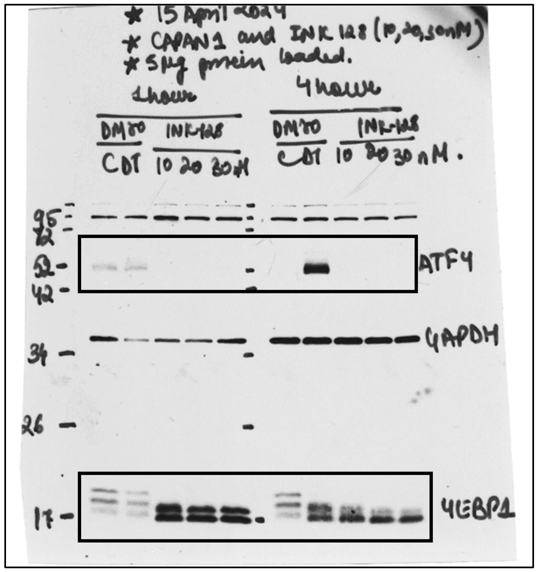

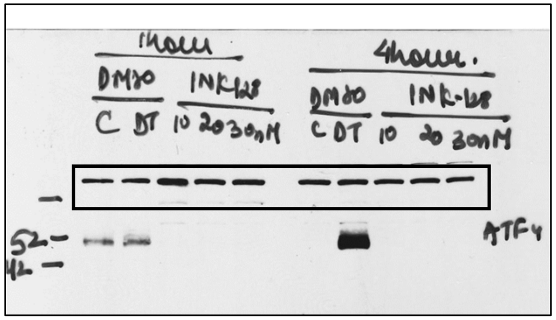

LC

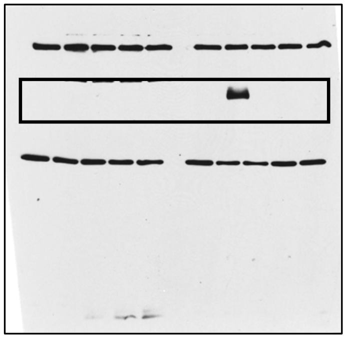

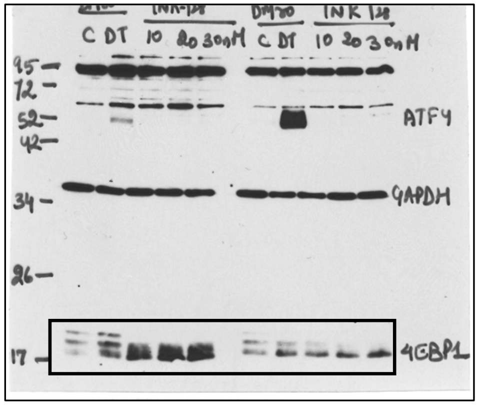

GAPDH

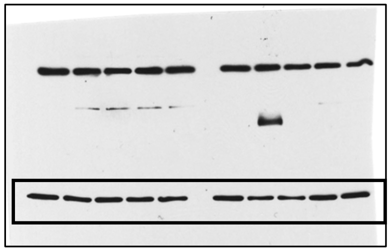

Full unedited blots for Figure 11D (continued)

11D

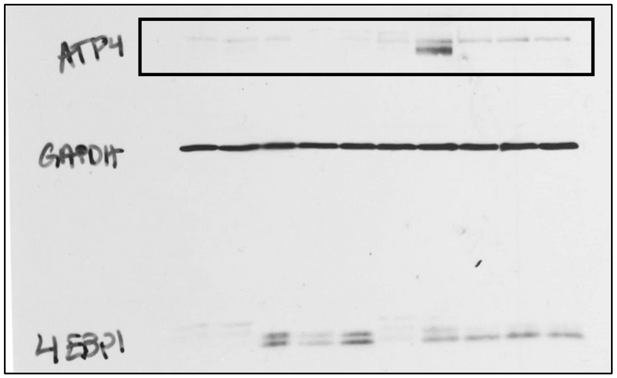

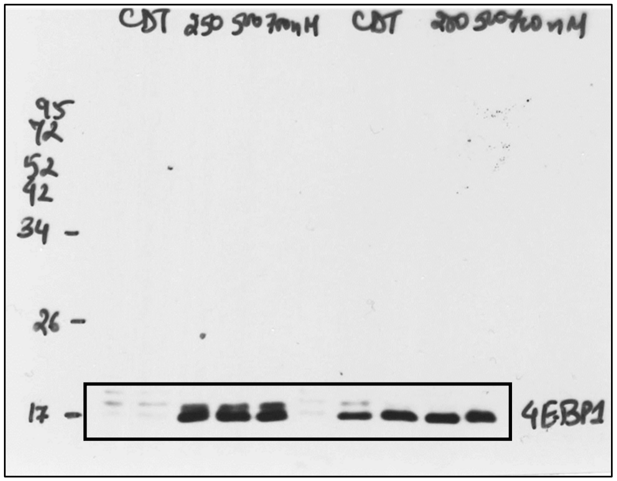

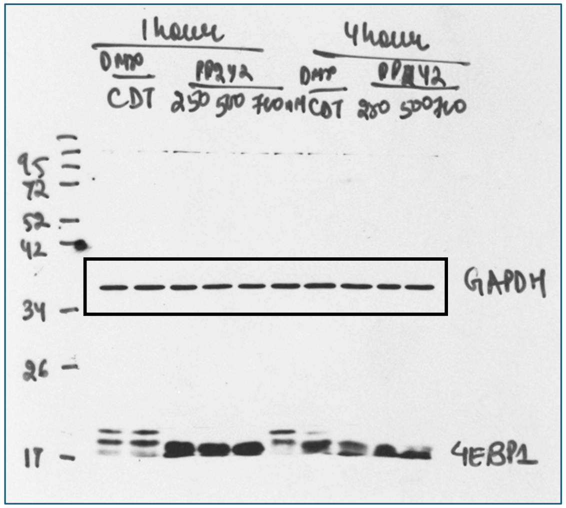

Full unedited blots for Figure 11F

11F

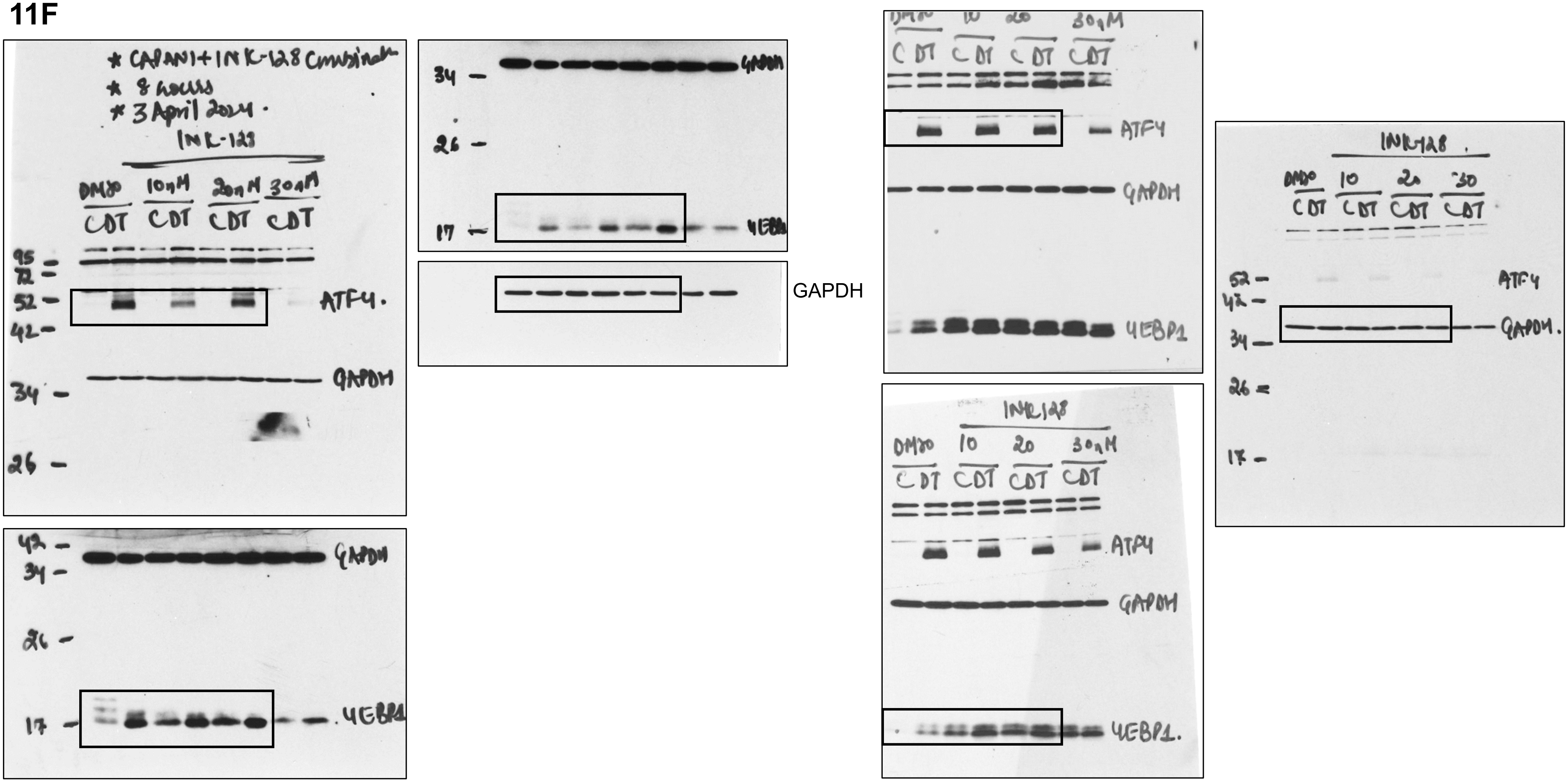

Full unedited blots for Figure 12C

12C

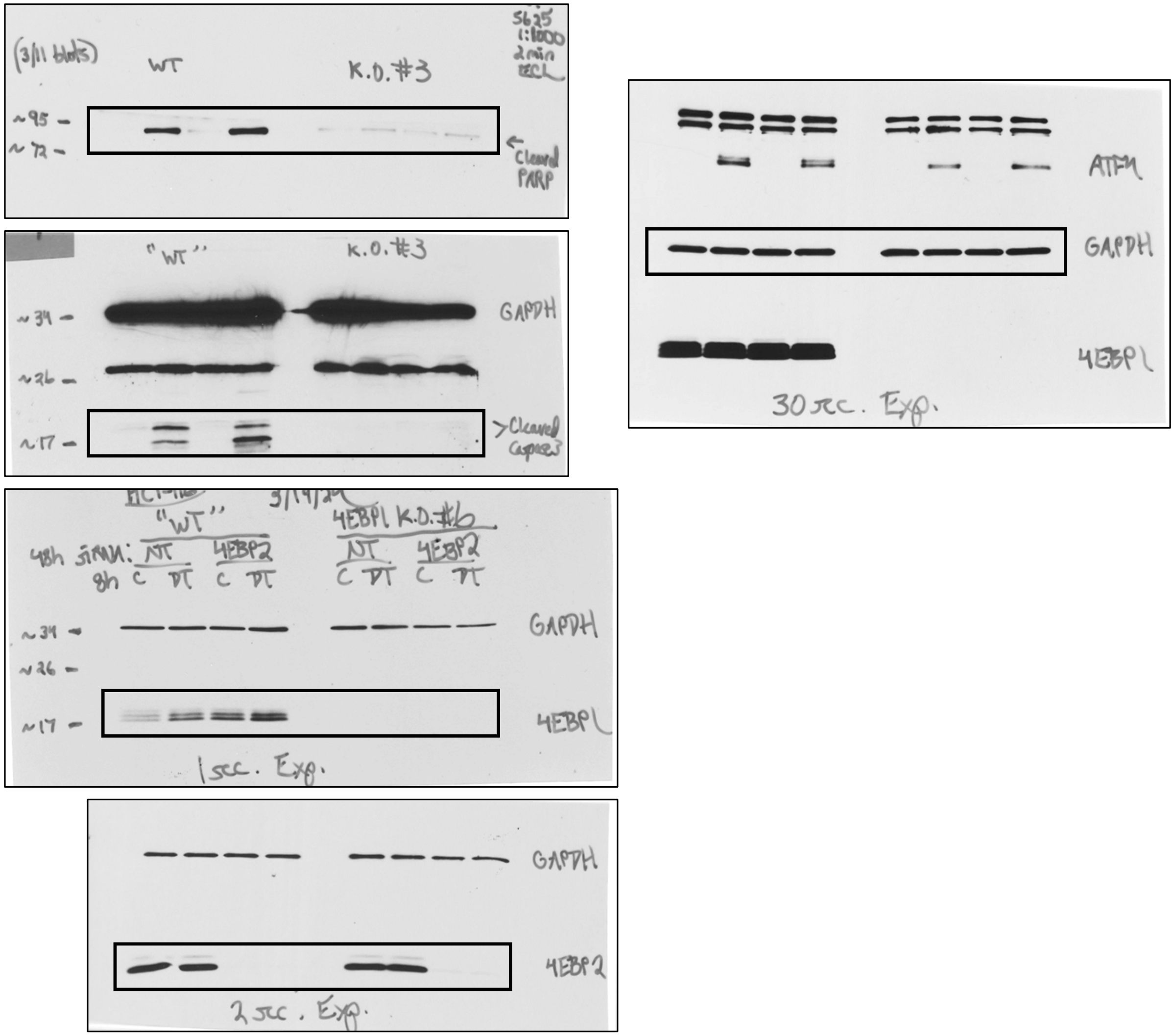

Full unedited blots for Figure 12C (continued)

12C

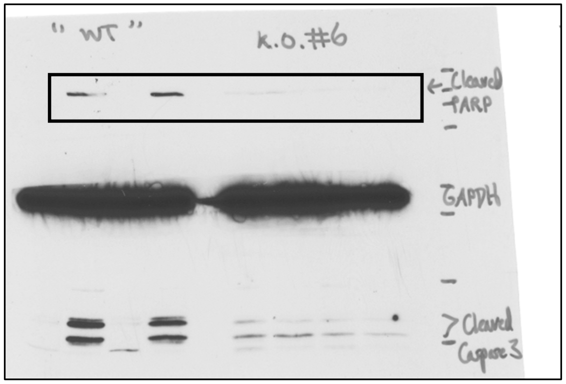

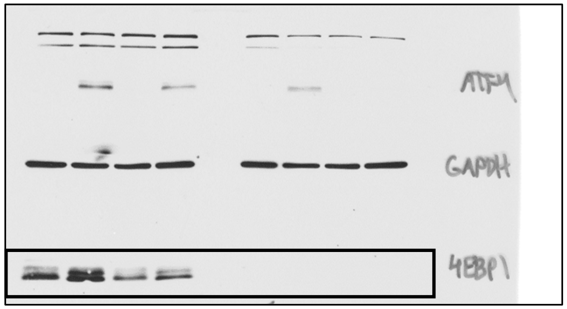

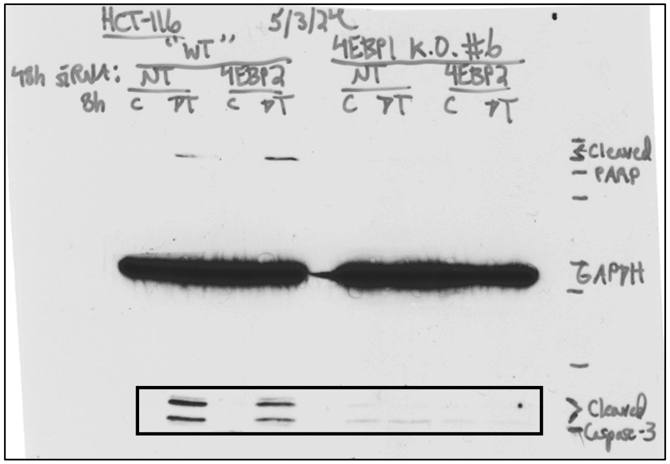

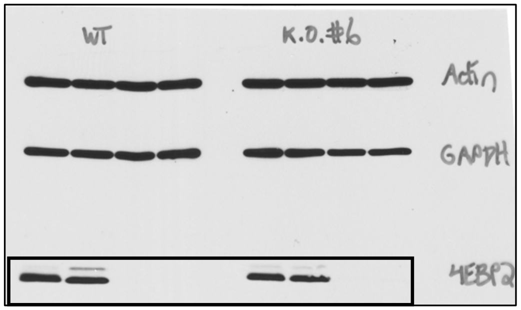

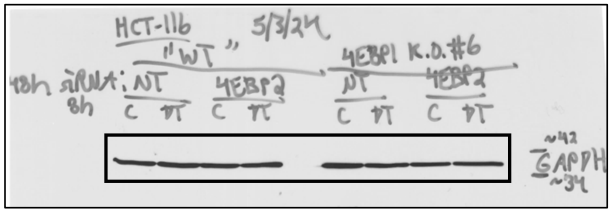

Full unedited blots for Figure 12D

12D

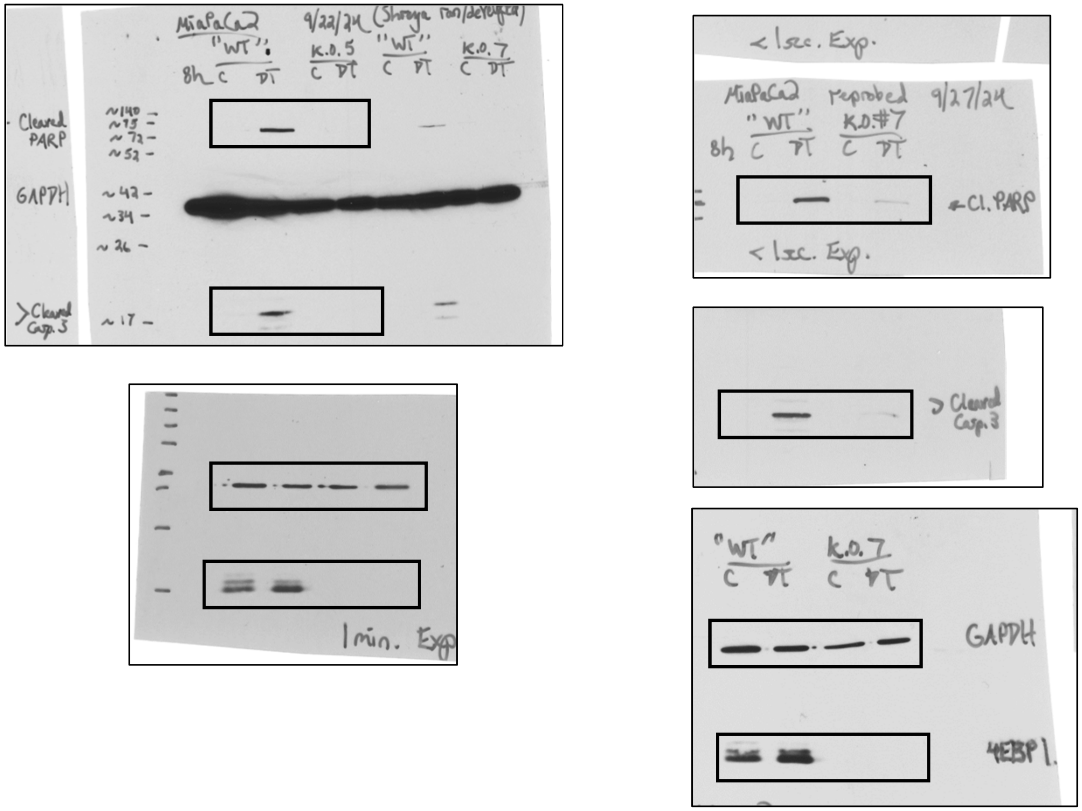

Full unedited blots for Figure 13B,C

13B

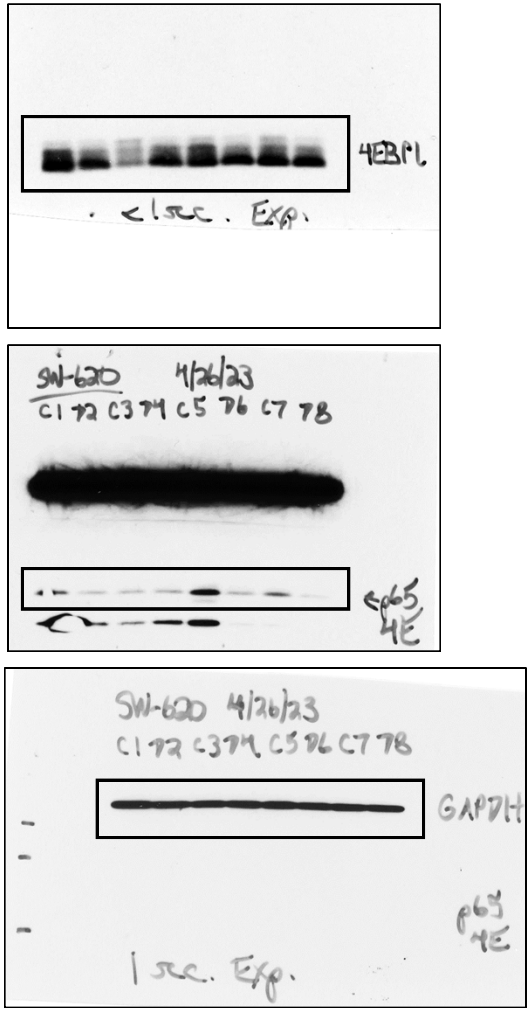

13C

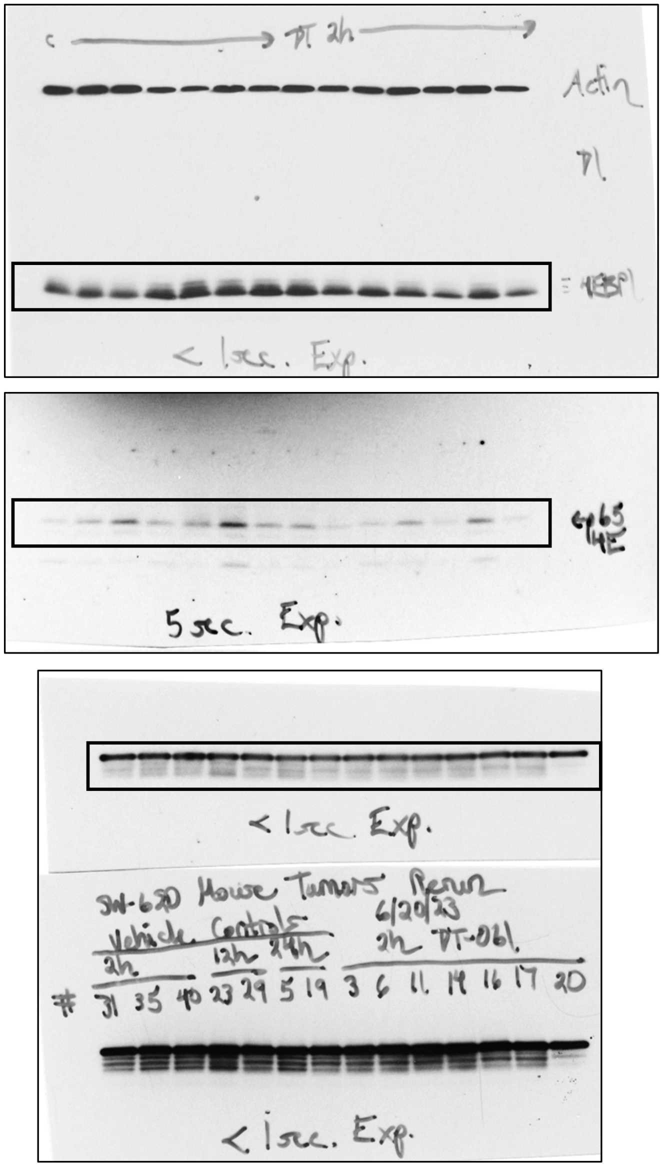

Full unedited blots for Figure 13D

13D

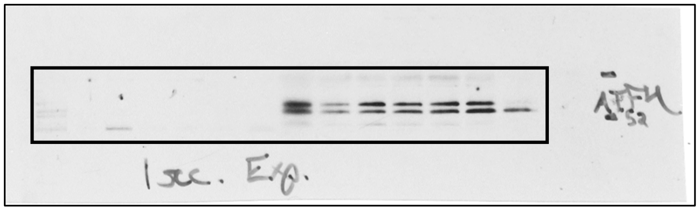

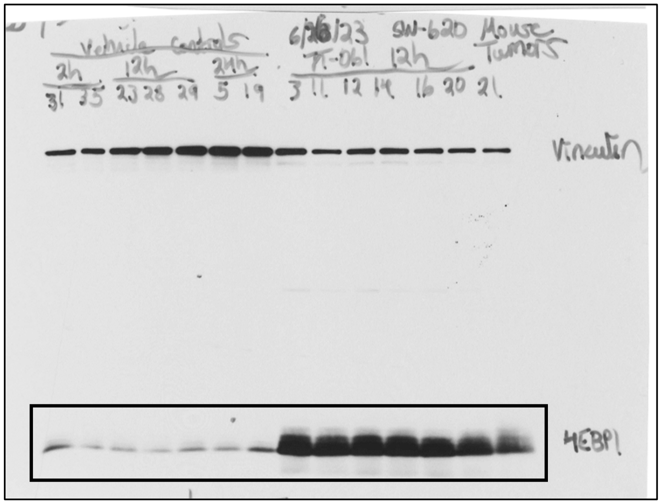

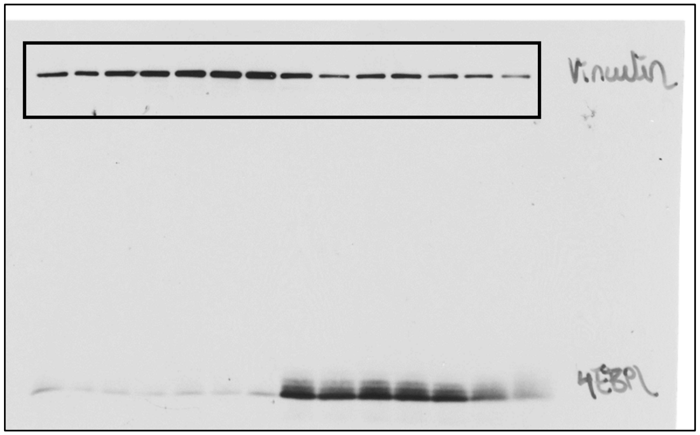

Full unedited blots for Supplemental Figure S3

S3

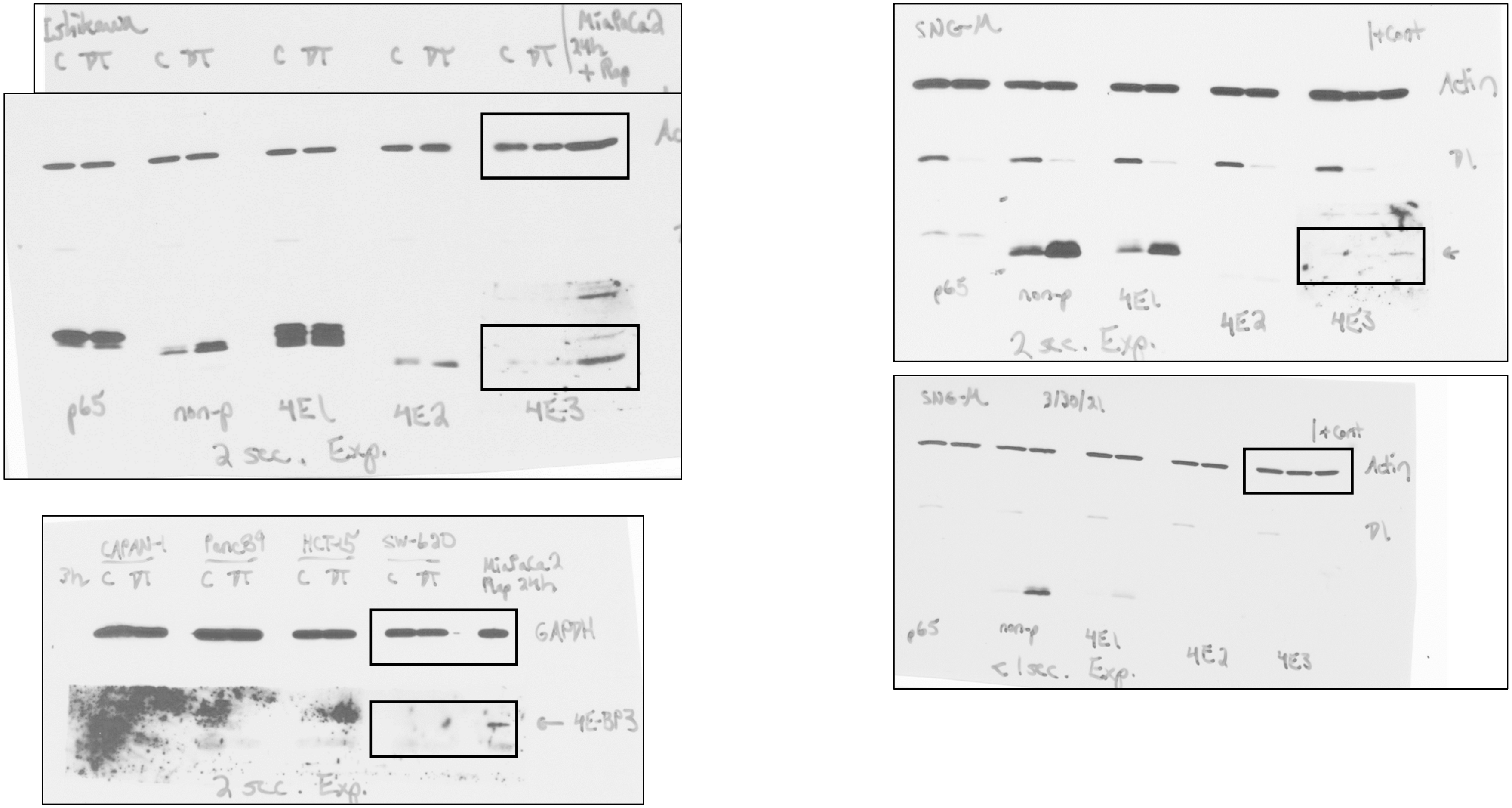

Full unedited blots for Supplemental Figure S5

S5

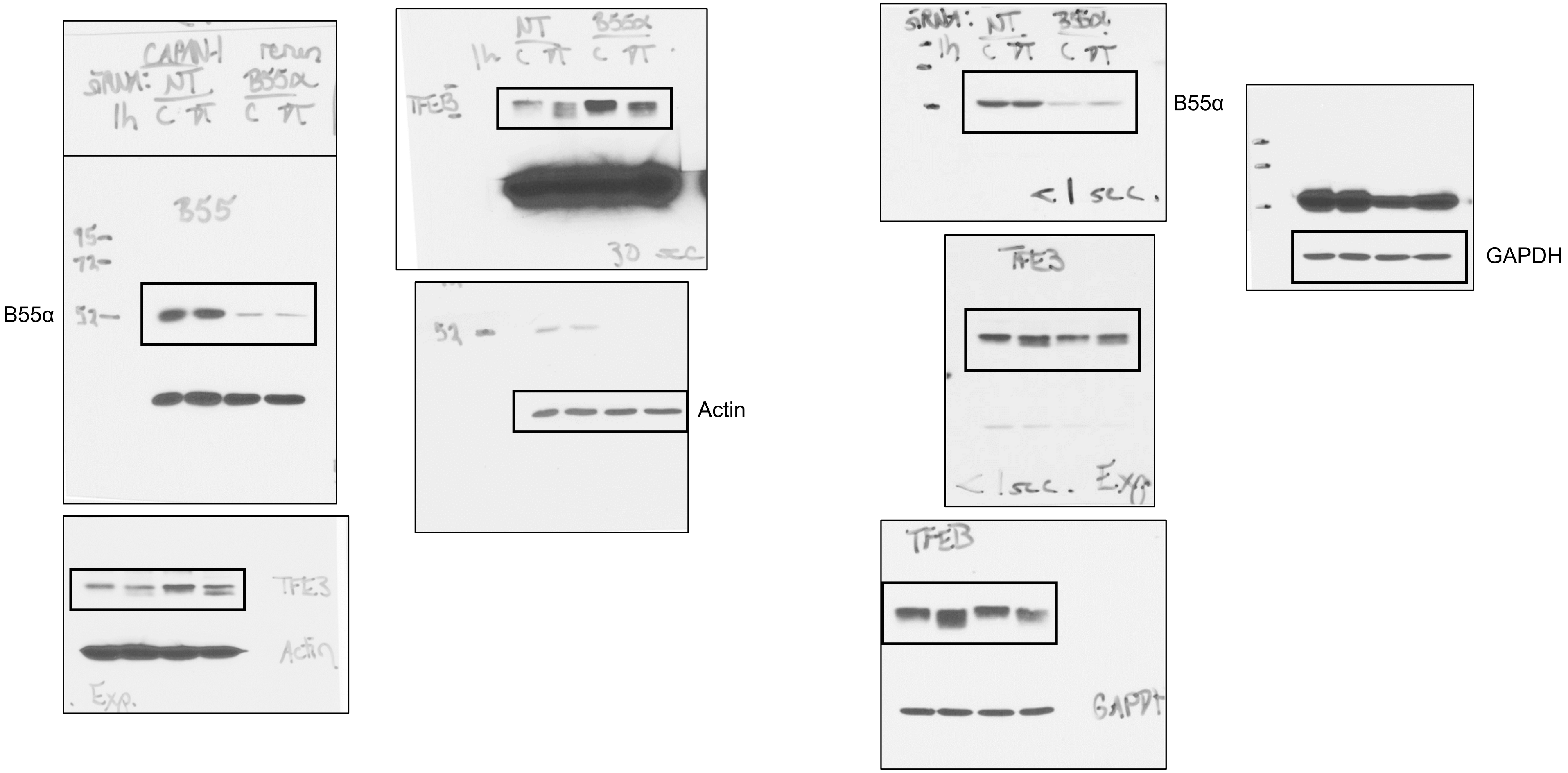

Full unedited blots for Supplemental Figure S6

S6

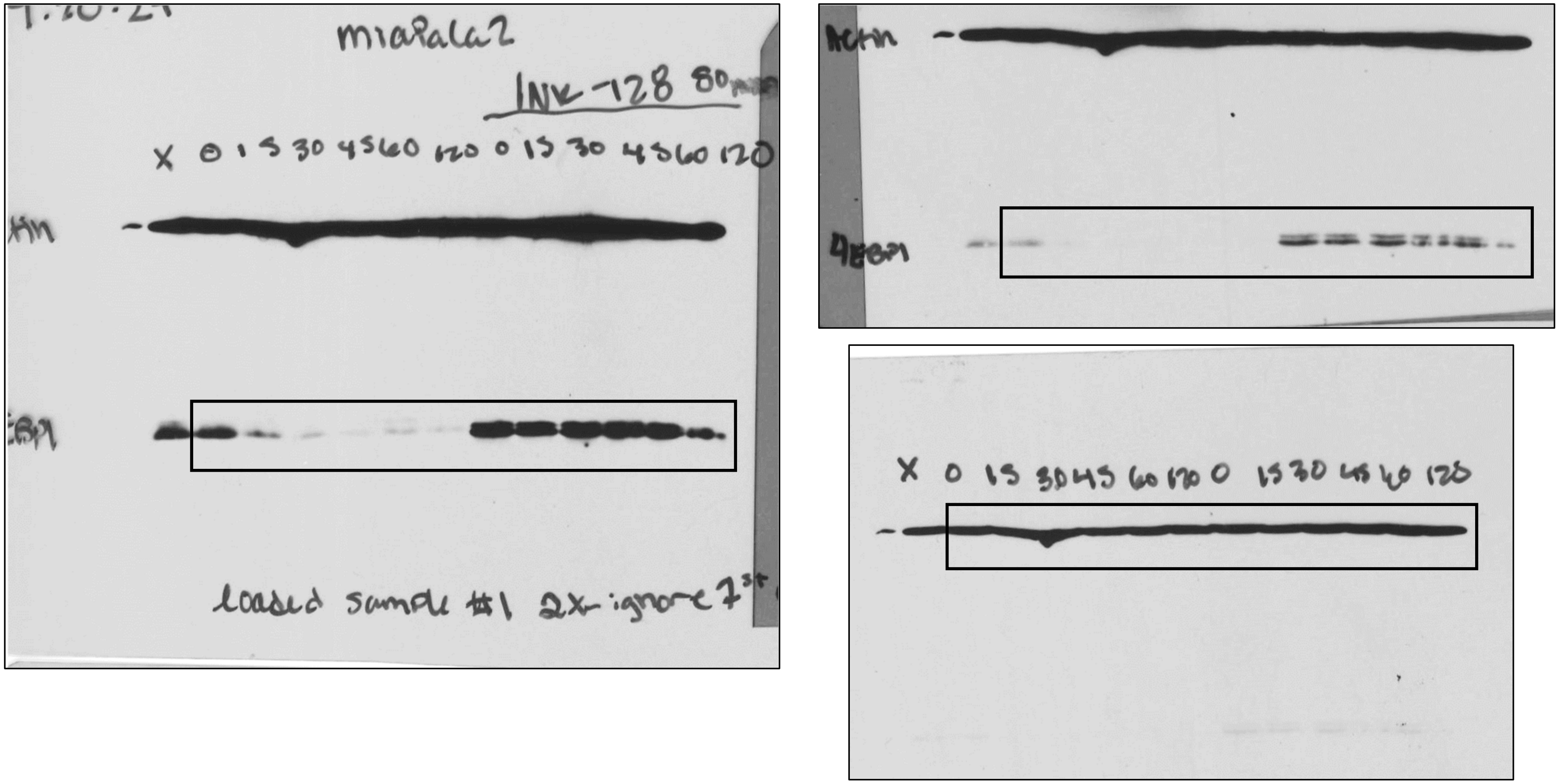

Supplement: Unedited blot and gel images [file jci-135-176093-s264.pdf]
